# Community cohesion and violence against women in Ghana, Pakistan, and South Sudan: A secondary data analysis

**DOI:** 10.1177/17455057221123998

**Published:** 2022-09-23

**Authors:** Sébastien Poix, Nuha Ibrahim, Stacey Scriver, Srinivas Raghavendra, Nata Duvvury, Khalifa Elmusharaf

**Affiliations:** 1 School of Medicine, University of Limerick, Limerick, Ireland; 2 Centre for Global Women’s Studies, School of Political Science and Sociology, University of Galway, Galway, Ireland; 3 Discipline of Economics, J.E. Cairnes School of Business and Economics, University of Galway, Galway, Ireland

**Keywords:** community cohesion, social cohesion, violence against women, intimate partner violence, family member violence, public spaces violence

## Abstract

**Background:**

Much knowledge has been accumulated on individual-level risks and protective factors of violence against women. However, the influence of factors operating at the community level, such as community cohesion, remains unclear, especially in low- and middle-income countries. This study examined whether community cohesion, a combined measure of mutual trust and tolerance, affects women’s likelihood of experiencing intimate partner violence, violence perpetrated by a family member, and violence occurring in public spaces.

**Methods:**

Data on 4785 women aged between 18 and 60 years in Ghana, Pakistan, and South Sudan, initially collected by the ‘What Works to Prevent Violence Against Women and Girls’ Research and Innovation Programme, were used for this study. Binary logistic regression analyses were used to assess the association between a composite measure of community cohesion and recent experience of physical, sexual, and psychological violence while controlling for different individual-, community-, and state-level variables.

**Results:**

Multivariate analyses revealed that community cohesion was associated with lower risks of public spaces violence (adjusted odds ratio = 0.396, 95% confidence interval = 0.312–0.503, P < 0.001) and family member violence (adjusted odds ratio = 0.839, 95% confidence interval = 0.754–0.934, P < 0.001). There was no statistically significant association between intimate partner violence and community cohesion, but adjusted models showed that women with more developed social networks were at higher risks of experiencing intimate partner violence (adjusted odds ratio = 1.104, 95% confidence interval = 1.062–1.148, P < 0.001).

**Conclusion:**

Our findings suggest that community cohesion may have a protective effect against the most visible forms of violence against women. However, this effect may be attenuated or even eliminated by other individual- or household-level mechanisms in the case of violence between intimates or family members.

## Introduction

Violence against women and girls (VAWG) is recognized as a violation of human rights and a significant public health issue.^1^ Globally, nearly a third (31%) of all women aged 15–49 years have already experienced physical or sexual violence during their lifetime.^2^ In most cases, this violence occurs in the home and is perpetrated by an intimate partner. It is estimated that 27% of ever-married women have experienced physical or sexual intimate partner violence, the prevalence remaining higher in low- and middle-income countries. As part of the UN Sustainable Development Goals (SDGs), the global community calls for eliminating all forms of violence against women in public and private spheres by 2030.^3^

Studies have shown that physical and sexual violence is associated with various adverse health outcomes, such as chronic and acute pains, poor sexual and reproductive health outcomes, and mental health issues.^4–7^ Aside from these impacts on health, a few studies demonstrated that violence against women had social and economic costs that not only affect the victims and their family circles but also the society at large.^8–10^ Findings from Ghana, Pakistan, and South Sudan showed that violence occurring in different settings translated into absenteeism and reduced productivity, with businesses incurring losses affecting, in turn, national economic stability and growth.

There is clear evidence that factors such as education level and socioeconomic status are protective against gender-based violence.^10^ However, substance abuse, childhood history of abuse, or history of violence between the parents are generally associated with higher risks of violence perpetration and victimization.^11,12^ While most studies focus on individual- and household-level predictors, there is a growing interest in how social-environmental factors influence violence victimization and perpetration. This interest builds on the recognition of violence against women as a complex and multifaceted phenomenon, as advocated by Heise in 1998.^13^ In her ecological framework, violent behaviours arise from the interplay of several factors existing at four different levels (individual, mesosystem, exosystem, macrosystem).

The social disorganization theory provides one of the most influential models to explain how the community context can shape the distribution of crime or other violent behaviours.^14^ According to this theory, communities with lower levels of social organization are more likely to have higher crime and delinquency rates because of their inability to maintain social control. Expanding on this theory, Sampson, Raudenbush, and Earls demonstrated that a higher level of collective efficacy, the combination of social cohesion and informal social control, was associated with lower levels of violence in the neighbourhoods of Chicago under study.^15^ The authors suggest that the mutual trust and cohesion between individuals living in the same neighbourhood play a significant role in the social processes through which social order is maintained.

Over the past two decades, several researchers have explored the influence of community-level factors^16–20^ on violence against women, but only a few have attempted to directly measure how community cohesion might influence these forms of violence. Although there is no consensus on the definition of social or community cohesion, most theories and frameworks share a joint theoretical base. For example, after identifying overlapping dimensions in most academic and policy-oriented studies published since the 1990s, Schiefer and van Der Noll defined social cohesion as ‘a descriptive attribute of a collective, indicating the quality of collective togetherness’.^21^

In a study of low-income African American women, Obasaju et al.^22^ found that participants living in neighbourhoods with higher levels of social cohesion were less likely to report intimate partner revictimization. Another study conducted in the United States showed little evidence of an association between community cohesion and intimate partner violence, suggesting that the strongest predictors of intimate partner violence occurred at the individual level.^23^ However, to the best of our knowledge, studies examining the potential role of community cohesion, or related constructs, on violence against women have been mainly conducted in high-income countries, and their conclusions may not be generalized to low- and middle-income countries where different dynamics may be at work in the perpetration and regulation of violence against women. Furthermore, previous studies focus on intimate partner violence, excluding other forms of violence against women, such as those occurring in public spaces.

This study consists of secondary data analysis of a large, representative sample of women from Ghana, Pakistan, and South Sudan. Its aim is to (1) assess the association between community cohesion and the past 12 months experience of physical, sexual, and/or psychological violence against women and to (2) examine whether the influence of community cohesion varies depending on where the violence occurs and who is the perpetrator (intimate partner violence, family member violence, public spaces violence). A secondary aim of this study was to (3) investigate the association between the experience of violence and social networking, measured at the individual level. This third objective was added to distinguish personal social bonds from the community’s degree of connectedness and examine potential interactions between these two constructs.

## Methods

### Study context

Pakistan, Ghana and South Sudan are traditional and patriarchal societies characterized by male dominance and control over women. In this type of society, social norms, cultural practices, and traditions contribute to maintaining men in a position of power and authority, while women and girls are often expected to be submissive to their husbands and fathers.^24^ Violence against women remains high in these three countries, with domestic violence being the most common form. Previous studies estimated that around 43%, 24%, and 31% of women aged 18–60 years had experienced intimate partner violence in the past 12 months in Ghana, Pakistan, and South Sudan, respectively.^8–10^ Despite these similarities, the three countries face different economic, social, and political realities. Ghana has one of the most stable political environments in Africa.^8^ Although gender-based discrimination remains common, the country has made significant progress in promoting women’s rights in recent years. Comparatively, Pakistan’s political context is more fragile. Also, Pakistan has more restrictive norms on women’s mobility and autonomy, which translates into restricted access to education and the health system, as well as limited opportunities to participate in the labour market.^9^ In South Sudan, several decades of wars and ongoing inter-communal conflicts have profoundly affected the country’s political and economic structures. To date, South Sudan ranks among the five lowest countries in terms of the Human Development Index.^25^ This situation, combined with a lack of formal legal protections and a strong influence of customary laws, has placed women and girls in a position of greater vulnerability.^26^ Even though years of conflicts have contributed to reinforcing social bonds within communities, mainly as a way to respond to external threats, intra-community violence, notably domestic violence, persists.^10^ Furthermore, disclosing violence can be even more difficult in a context where maintaining social cohesion is considered a vital interest.

### Study design

We used a quantitative descriptive cross-sectional study including 4785 women aged 18–60 years who reported having a husband or partner living in Ghana, Pakistan, or South Sudan. The Strengthening the Reporting of Observational Studies in Epidemiology (STROBE) guidelines were used as a reference for preparing and writing this study.^27^

### Data

Data for this study were collected in 2016 as part of the ‘What Works to Prevent Violence Against Women and Girls’ Research and Innovation Programme to understand better the social and economic costs of violence against women in Ghana, Pakistan, and South Sudan. Face-to-face in-home interviews were conducted among a sample of 6996 women aged between 18 and 60 years and living in private, residential households in 2016. The original questionnaire included 295 questions. It was pre-tested through a series of cognitive interviews and then pilot-tested in April and May 2016. The original data set and the questionnaire are available online under Universal Public Domain Dedication.^28^

The participants were randomly selected from 291 Primary Sampling Units (PSUs) across the three countries. The PSUs were determined with the respective National Bureau of Statistics and correspond to each country’s lowest census sampling unit. The original study received ethical approval from the National Bioethics Committee Pakistan (4-87/16/NBC-210/RDC/389) and the University of Ghana’s Ethics Committee for the Humanities (ECH 018/15-16) for Pakistan and Ghana, respectively. For South Sudan, the original study was approved by the National University of Ireland Galway’s Ethics Committee (15/Sept/16), and the research protocol was reviewed and approved by the National Bureau of Statistics of South Sudan. All the participants were assured of confidentiality and asked to sign a consent form. If they could not sign, they were asked to place an ‘X’ as evidence they agreed to participate in the survey. More detailed descriptions of the sampling, data collection methods, and ethical considerations were published in 2019.^8–10^

### Inclusion/exclusion criteria and sample size

We only included women aged between 18 and 60 years who reported having a husband or a partner at the moment of the study (N = 4800). This decision was made to facilitate the comparison between intimate partner violence and violence occurring in the two other settings. In order to avoid a representativeness bias when calculating the community cohesion score at the community level, we excluded women who lived in a PSU with fewer than five individuals. As a result, the final data set included 4785 women from 284 PSUs. The average number of women per PSU was 17, with a minimum of 5 and a maximum of 46 women. For this analysis, the PSU was considered the most appropriate basis to determine and delimit the community level; therefore, individuals from the same PSU were considered pertaining to the same community. Therefore, only the term ‘community’ is used in the following sections to avoid confusion.

We conducted an a priori power analysis using G*Power 3.1. The power analysis was replicated three times, assuming a statistical power of 0.80 and an error probability of 0.05. The analysis, based on the difference in mean values, revealed that sample sizes of 259, 257, and 698 were needed for intimate partner violence, family member violence, and public space violence, respectively. The inclusion of 4785 individuals in the study ensured sufficient statistical power to detect effects of interest, even in the presence of several missing values.

### Measurements

#### Experience of violence

The dependent variables were past 12 months experience of physical, sexual, and psychological violence in three different settings: (1) the home with the perpetrator being the husband or a partner, (2) the home with the perpetrator being a member of the family other than the husband or partner, and (3) public spaces. In coherence with the original study, public spaces violence was defined as violence perpetrated by known or unknown persons in public transport, streets, squares, and markets. The measurement did not include violence at the workplace and within educational institutions in this study. Thirty-one items (13 for intimate partner violence, 13 for family member violence, 5 for public spaces violence) from the original questionnaire were used to measure the experience of three different forms of violence (physical, sexual, or psychological) the participants may have experienced over the 12 months preceding the questionnaire. Responses were aggregated to create a dichotomous variable indicating the presence or absence of at least one act of violence during the past year (0 = none, 1 = one or more acts of violence).

A value was assigned to an individual only when clear evidence of the presence or absence of an act of violence was available. Therefore, missing values were assigned to all the participants who reported no experience of violence and at least one invalid answer (do not know, not stated, prefer not to say). Although this approach resulted in many missing values, this ensures that no invalid answers were misinterpreted. To assess the impact of this assumption on the results, we conducted multiple sensitivity analyses comparing the results obtained when using the median of nearby point imputation method in IBM Statistical Package for Social Sciences (SPSS) version 26. As a result, we observed a difference in odds ratios in the association between community cohesion and family member violence (+8.61%), community cohesion and public spaces violence (+21.89%), and social networking and intimate partner violence (−2.022%). If these results indicate that our treatment of invalid answers may have affected the magnitude of the relationship, it is essential to note that the direction of the association and the degree of significance (P < 0.001) remained unchanged.

#### Community cohesion

**Figure 1.**
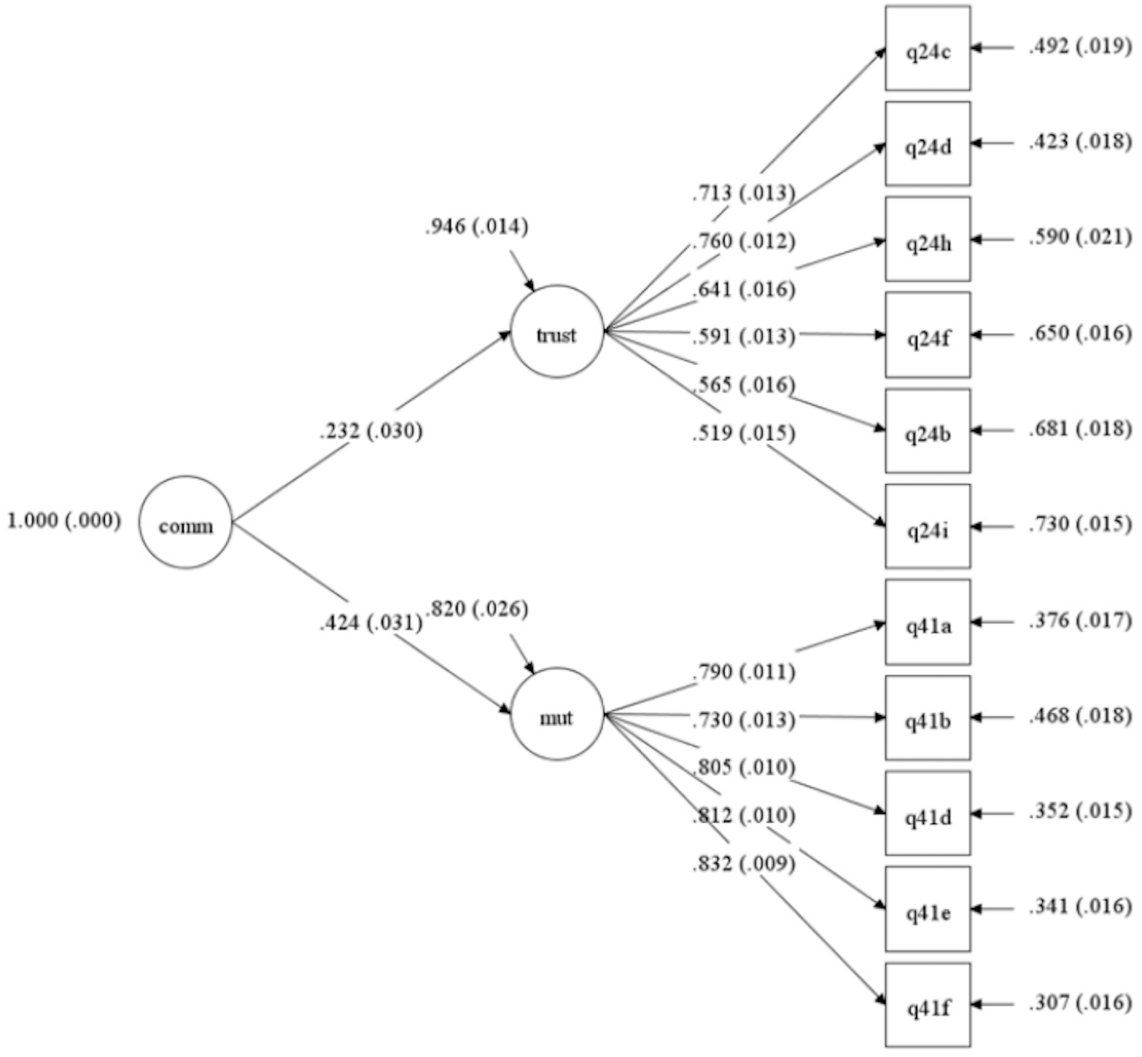
Confirmatory factor analysis (CFA) model for community cohesion. comm: community cohesion score; trust: trust; mut: mutual tolerance; q24b: how much do you trust different groups of people who live and work in your local community: people from another linguistic, caste, tribe, or religious group?; q24c: how much do you trust different groups of people who live and work in your local community: shopkeepers?; q24d: how much do you trust different groups of people who live and work in your local community: local government officials?; q24h: how much do you trust different groups of people who live and work in your local community: politicians?; q24f: how much do you trust different groups of people who live and work in your local community: teachers?; q24i: how much do you trust different groups of people who live and work in your local community: religious leaders?; q4la: please tell me if the differences between people with different social status ever lead to problems in your community?; q41b: please tell me if the differences between men and women ever lead to problems in your community?; q41d: please tell me if the differences between people with different political affiliations ever lead to problems in your community?; q41e: please tell me if the differences between people with different religious beliefs ever lead to problems in your community?; q41f: please tell me if the differences between people with different ethnic backgrounds ever lead to problems in your community?

Community cohesion is generally seen as a characteristic of a society, indicating the degree of connectedness and togetherness among its members, although there is no clear consensus about what community cohesion encompasses and how the construct can be operationalised.^21^ In this study, we took a minimalistic approach and defined community cohesion as the combination of (1) trust and (2) mutual tolerance between community members. These two dimensions are measurable and regularly appear as core components of social cohesion in the literature.^21^ A composite measure of perceived community cohesion was calculated and aggregated at the community level. Several steps were needed to build the community cohesion score. First, 35 items potentially indicative of community cohesion were identified from the original questionnaire. These items reflected different components of the construct (i.e. mutual tolerance, trust, solidarity, social and civic participation) and were selected based on theoretical models or measures of social cohesion.^15,21,29^ Second, exploratory factor analysis was conducted to examine these items and identify underlying factors of the construct. This analysis revealed a two-factor model including 12 items, with the two factors accounting for 57.77% of the variance in the sub-sample (N = 3498). This model’s construct validity and reliability were examined and refined through confirmatory factor analysis (CFA). The final model for community cohesion included 11 items (Figure 1). The first factor (five items) captured the construct of mutual tolerance. The related items indicated if participants felt that differences between different groups ever led to problems in the community. The second factor (six items) captured the degree of trust among the community members. The related items indicated how much the participants trusted different community groups. Additional details regarding the factor analyses are reported as Supplemental Material. Third, the final score was aggregated at the community level and expressed as a single value ranging from 0 to 10, where higher values indicated higher levels of community cohesion. Overall, mean community cohesion scores were assigned to the 284 communities based on 3693 data points out of 4875. The 1092 (23%) scores that were not included in the calculation of the mean community cohesion score relate to individuals who did not answer to at least 1 item among the 11 items entering into the composition of the community cohesion score

#### Social networking

The social networking variable consists of a score reflecting the quantity and quality of an individual’s social interactions. Contrary to community cohesion, which is considered a community characteristic, social networking is measured individually. Four items from the original questionnaire were summed to build the social networking score. The selected items assessed how many friends, neighbours, or family members the participants were in regular contact with and the number of people they could turn to when they had a personal issue. An exploratory factor analysis was conducted and revealed a one-factor model, accounting for 49.21% of the variance in the sub-sample (N = 3498). This model was then tested through CFA. More detailed information about the items and the results of these analyses is reported as Supplemental Material. The final score was expressed as a single value ranging from 0 to 10, where 0 indicated the lowest level of social networking and 10 the highest. Overall, mean social networking scores were assigned to the 284 communities based on 4618 data points out of 4875. The Pearson correlation coefficient between social networking and community cohesion was −0.188 (P < 0.001), confirming that both scores captured distinct constructs, with no or little overlap between them.

#### Covariates

Five individual-level variables, one community-level variable, and one country-level variable were included in the analysis as possible confounders. Individual-level variables included age group (1 = 18–30 years old, 2 = 31–60 years old), level of education (1 = none, 2 = primary, 3 = secondary, 4 = higher), number of years spent in the neighbourhood (1 = less than 2 years, 2 = between 2 and 10 years, 3 = more than 10 years), being involved in paid market work (1 = yes, 2 = no), and personal autonomy. The level of personal autonomy was measured from a single question where the participants were asked: ‘to what extent, if at all, do you feel you have control in making your own decisions that affect everyday activities?’. The 4-point Likert-type scale was recoded to create three distinct categories (1 = lower level of perceived autonomy, 2 = intermediate level of perceived autonomy, 3 = higher level of perceived autonomy). A variable indicating whether the community was in an urban or rural area (1 = rural, 2 = urban) was included for the community level. Finally, the country of residence (1 = Ghana, 2 = Pakistan, 3 = South Sudan) was included as a state-level variable.

#### Data analysis

Exploratory factor analyses and confirmatory factor analyses were performed using IBM Statistical Package for Social Sciences (SPSS) version 26 and MPLUS version 8.6. All other statistical analyses were conducted using SPSS version 26. Data were weighted to reflect the demographic characteristics of the population of Ghana, Pakistan, and South Sudan. The weighting factor was provided with the data set and was already used in the original research.^8–10^ A significance level of 5% was used for all the analyses described below.

Descriptive statistics, including frequencies for the sociodemographic variables and mean values for the scores, were calculated to describe the characteristics of the sample. Pearson’s chi-square test was conducted to assess the potential sociodemographic differences between the group of women who experienced violence and those who did not experience violence. For the score variables, mean differences between these two groups were assessed using an independent *t*-test. Multivariable binary logistic regression analyses were conducted to determine the adjusted odds ratio (AOR) between community cohesion and social networking and individuals’ experience of violence, controlling for individual, community, and state-level covariates. Multivariate analyses were performed in the same way for the three studied types of violence (intimate partner violence, family member violence, public spaces violence) to allow comparisons of the results.

## Results

### Characteristics of the sample

Table 1 shows the characteristics of the sample and the mean scores for community cohesion and social networking. In total, 4785 women were included in the study. Of the total women, 26.8% lived in Ghana, 50.7% in Pakistan, and 22.5% in South Sudan. The most prevalent type of violence reported by the participants was public spaces violence (88.9%), followed by intimate partner violence (37.3%) and family member violence (37.1%). In addition, 55.2% of the women were aged between 31 and 60 years, and 58.9% lived in rural areas. Overall, 47.5% of the participants have no formal education, and approximately two-thirds (66.0%) were not involved in any paid market work. Fifty-eight percent of the participants lived in the same area for more than 10 years (58.0%). In comparison, 10.0% lived in the same area for less than 2 years. The percentage of women with lower, intermediate, and higher levels of perceived autonomy was 25.3%, 43.8%, 30.9%, respectively. Regarding the score variables, the mean community cohesion score was 5.47 (standard deviation (SD) = 1.71), and the mean social networking score was 5.10 (SD = 2.53).

**Table 1.** Characteristics of the sample (weighted).

Variables	% or mean (SD)	Range
Dependent variables		
Intimate partner violence (N = 3894)	37.3%	
Family member violence (N = 2976)	37.1%	
Public spaces violence (N = 1103)	88.9%	
Independent variables		
Community cohesion (N = 4332)	5.47 (1.71)	0–10
Social networking (N = 3763)	5.10 (2.53)	0–10
Covariates		
Age group (N = 4332)		
18–30 years old	44.8%	
31–60 years old	55.2%	
Education level (N = 4279)		
None	47.5%	
Primary	17.7%	
Secondary	27.0%	
Higher	7.8%	
Time spent in the area (N = 3796)		
Less than 2 years	10.0%	
Between 2 and 10 years	32.0%	
More than 10 years	58.0%	
Paid market work (N = 4229)		
Yes	34.0%	
No	66.0%	
Autonomy (N = 4205)		
Lowest	25.3%	
Intermediate	43.8%	
Highest	30.9%	
Location (N = 4332)		
Rural	58.9%	
Urban	41.1%	
Country (N = 4332)		
Ghana	26.8%	
Pakistan	50.7%	
South Sudan	22.5%	

SD: standard deviation.

### Bivariate analysis

Table 2 shows the bivariate associations between individual-, community-, and state-level characteristics and the experience of violence occurring across the three different settings. Compared to women who did not experience intimate partner violence, women who reported intimate partner violence were more likely to have a lower education level, be involved in paid market work, and live in a rural area. The proportion of women who experienced intimate partner violence was also higher among women with lower levels of perceived autonomy. Again, significant differences can be observed between countries, with South Sudan exhibiting a higher proportion of women reporting intimate partner violence. Regarding family member violence, the proportion of women who experienced violence is higher among women involved in paid market work and women with lower levels of perceived autonomy. For violence occurring in public spaces, women who reported this type of violence were more likely to have higher levels of perceived autonomy, live in an urban area, and not be involved in paid market work. Contrary to intimate partner violence and family member violence, the prevalence of public spaces violence is lower in South Sudan than in the two other countries. Overall, only three variables retained a 5% significance level across the three types of studied violence: paid market work, perceived autonomy, and country.

**Table 2.** Experience of intimate partner violence, family member violence, and public spaces violence by socioeconomic characteristics (weighted).

Variables	Intimate partner violence		Family violence		Public spaces violence	
	Yes (%)	P-value	Yes (%)	P-value	Yes (%)	P-value
Age group						
18–30 years old	38.1%	0.311	38.4%	0.219	88.4%	0.602
31–60 old	36.5%		35.1%		89.4%	
Education level						
None	40.8%	<0.001	34.4%	0.004	89.2%	0.841
Primary	42.1%		47.5%		87.2%	
Secondary	32.8%		35.5%		89.5%	
Higher	22.4%		35.6%		89.4%	
Time spent in the neighbourhood						
Less than 2 years	37.9%	0.006	40.2%	0.001	90.8%	0.654
Between 2 and 10 years	41.3%		43.0%		88.2%	
More than 10 years	35.4%		32.0%		87.8%	
Paid market job						
Yes	52.0%	<0.001	45.5%	<0.001	86.2%	0.009
No	30.8%		33.1%		91.2%	
Perceived autonomy						
Lower	58.1%	<0.001	50.9%	<0.001	83.9%	0.002
Intermediate	30.6%		32.1%		91.3%	
Higher	31.2%		34.0%		90.8%	
Location						
Rural	40.0%	<0.001	38.0%	0.391	87.1%	0.016
Urban	33.2%		35.6%		91.7%	
Country						
Ghana	39.0%	<0.001	44.0%	<0.001	95.7%	<0.001
Pakistan	23.9%		28.8%		93.1%	
South Sudan	70.6%		49.1%		83.9%	

Table 3 shows the mean differences in the community cohesion and social networking scores between women who experienced intimate partner violence, family member violence, or public spaces violence and those who did not experience these types of violence. Overall, the mean community cohesion score was lower among women who experienced violence than those who did not. This observation is valid across the three types of violence being studied, although the difference was more pronounced with intimate partner violence and family member violence. When it comes to violence occurring in public spaces, the difference in mean values between the two groups was small but is at the threshold for significance. Regarding social networking, a higher mean score was observed in women who experienced intimate partner violence than those who did not. Conversely, the mean social networking score was lower among women who experienced public spaces violence than those who did not. Finally, there was no statistically significant difference in the mean social networking score between women who experienced family member violence and those who did not.

**Table 3.** Experience of intimate partner violence, family member violence, and public spaces violence by mean community cohesion score and social networking scores (weighted).

Variables	Intimate partner violence			Family member violence			Public spaces violence		
	Yes	No	P-value	Yes	No	P-value	Yes	No	P-value
	Mean (SD)	Mean (SD)		Mean (SD)	Mean (SD)		Mean (SD)	Mean (SD)	
Community cohesion	5.04 (1.64)	5.86 (1.55)	<0.001	5.00 (1.53)	5.64 (1.77)	<0.001	4.52 (1.86)	4.77 (1.24)	0.050
Social networking	5.70 (2.40)	4.77 (2.44)	<0.001	5.62 (2.22)	5.48 (2.28)	0.319	5.57 (2.38)	6.04 (1.92)	0.016

### Multivariate analysis

Table 4 shows the results of three multivariate binary logistic regression analyses assessing the association between women’s likelihood of experiencing violence and the level of community cohesion and social networking. The three analyses were conducted using the same covariates. Nagelkerke’s *R*^2^ for the multivariable models predicting the experience of intimate partner violence, family member violence, and public spaces violence were 0.259, 0.111, and 0.227, respectively.

**Table 4.** Binary logistic regression models assessing the effects of community cohesion and social networks on intimate partner violence, family member violence, and public spaces violence (weighted).

Variables	Intimate partner violence		Family member violence		Public spaces violence	
	AOR (95% CI)	P-value	AOR (95% CI)	P-value	AOR (95% CI)	P-value
Community cohesion	0.966 (0.902–1.034)	0.319	0.839 (0.754–0.934)	<0.001	0.396 (0.312–0.503)	<0.001
Social networking	1.104 (1.062–1.148)	<0.001	1.000 (0.935–1.069)	0.995	1.024 (0.919–1.142)	0.662
Age group						
18–30 years old	REF		REF		REF	
31–60 years old	1.087 (0.901–1.311)	0.385	0.917 (0.671–1.253)	0.586	0.982 (0.588–1.643)	0.946
Education level						
None	REF		REF		REF	
Primary	0.949 (0.745–1.209)	0.673	1.193 (0.821–1.734)	0.354	0.670 (0.385–1.166)	0.156
Secondary	0.913 (0.724–1.152)	0.445	0.854 (0.582–1.252)	0.418	0.406 (0.201–0.825)	0.012
Higher	0.542 (0.375–785)	0.001	1.292 (0.769–2.170)	0.334	0.415 (0.172–1.001)	0.050
Time spent in the neighbourhood						
Less than 2 years	REF		REF		REF	
Between 2 and 10 years	0.954 (0.713–1.278)	0.753	1.014 (0.642–1.601)	0.954	0.913 (0.419–1.988)	0.818
More than 10 years	0.950 (0.678–1.332)	0.767	0.579 (0.355–0.944)	0.028	1.322 (0.585–2.989)	0.502
Paid market work						
Yes	REF		REF		REF	
No	0.871 (0.703–1.079)	0.207	1.052 (0.734–1.509)	0.782	1.352 (0.801–2.281)	0.259
Perceived autonomy						
Lower	REF		REF		REF	
Intermediate	0.499 (0.403–0.616)	<0.001	0.515 (0.375–0.707)	<0.001	1.553 (0.919–2.623)	0.100
Higher	0.412 (0.323–0.525)	<0.001	0.581 (0.397–0.850)	0.005	1.668 (0.891–3.123)	0.110
Location						
Rural	REF		REF		REF	
Urban	0.967 (0.793–1.178)	0.736	0.815 (0.587–1.131)	0.221	1.197 (0.664–2.160)	0.549
Country						
Ghana	REF		REF		REF	
Pakistan	0.375 (0.275–0.510)	<0.001	0.560 (0.335–0.938)	0.027	0.278 (0.077–1.003)	0.051
South Sudan	2.018 (1.418–2.871)	<0.001	0.732 (0.404–1.328)	0.305	0.011 (0.003–0.045)	<0.001

AOR: adjusted odds ratio; CI: Confidence of interval; REF: reference.

Overall, there was no statistically significant association between community cohesion and the risk of intimate partner violence. In contrast, a significant negative association was found between community cohesion and the two other types of violence. Specifically, a one-unit increase in the community cohesion score was associated with a decrease in the risk of family member violence by 16% (AOR = 0.839, 95% confidence interval (CI) = 0.754–0.934, P < 0.001) and public spaces violence by 60% (AOR = 0.396, 95% CI = 0.312–0.503, P < 0.001). Social networking was not associated with family member violence and public spaces violence in our models, but a slight positive association was found with intimate partner violence. Each one-unit increase in the social networking score was associated with a 10% increase in the risk of experiencing intimate partner violence (AOR = 1.104, 95% CI = 1.062–1.148, P < 0.001).

## Discussion

This study aimed to investigate the influence of community cohesion on women’s likelihood of experiencing intimate partner violence, family member violence, and public spaces violence in Ghana, Pakistan, and South Sudan.

Our results showed that intimate partner violence was not associated with community cohesion but that women living in more cohesive communities had a lower risk of experiencing public spaces violence and, to a lesser extent, family member violence. These findings suggest that community cohesion plays an essential part in regulating public forms of violence against women. According to the social disorganization theory, the ability of a community to perform social control largely depends on the degree of cohesiveness among its members.^15^ Individuals living in more cohesive communities share a common understanding of what constitutes a safe environment and, thus, may be more willing to regulate proscribed behaviours and attitudes, especially if they know that other community members would support them.^30^ Overall, these results show that the regulation of violence against women in public spaces relies on the same social processes involved in regulating crime and delinquency in general. If this assumption is correct, higher levels of community cohesion should reflect on higher levels of informal social control, which we did not measure here.

On the contrary, these mechanisms do not apply to intimate partner violence since no statistically significant association was found between community cohesion and this form of violence. These findings are consistent with previous research that found no or little evidence about the protective effect of social cohesion on violence between intimates.^23,31^ A possible explanation is that even in communities with higher social cohesion and informal social control, neighbours might choose not to intervene in private matters.^23^ Another explanation might lie in the greater susceptibility of intimate partner violence to social and cultural norms.^30^ Contrary to violence occurring in public spaces, the attitudes of individuals towards intimate partner violence remain primarily dependent on how this form of violence is perceived and socially embedded in the community. Thus, in communities where intimate partner violence is tolerated, the protective effect of community cohesion may be counterbalanced or even eliminated. The relationship between family member violence and community cohesion is not clear. If this study showed that the risk of experiencing an act of violence perpetrated by a family member is higher in less cohesive communities, the relatively weak magnitude of this association suggests that the influence of community cohesion is only minor. It is also difficult to draw clear conclusions since the structure, and family functioning can vary significantly among the three studied countries. However, the influence of community cohesion on family violence, although weak, suggests that such violence may spill into public view more easily than violence occurring in intimate relationships, which is perceived as private. Overall, the findings revealed that community cohesion does not relate to all forms of violence against women in the same way. To better understand these differences, further studies could examine the potential role of gender norms and individuals’ attitudes in mediating the association between community cohesion and each of these forms of violence.

In addition, this study showed that women with strong social networks had slightly higher risks of experiencing intimate partner violence. A possible explanation has been advanced in qualitative research conducted in Ghana.^32^ In this study, researchers found that women in positions of leadership, who tend to have greater social networks, would hide violence they experienced to avoid being seen as unworthy of their roles. If it were known, their qualities as leaders would be contested, and they would have to give up their responsibilities within the community. In this way, social networking may keep a woman in an abusive relationship rather than create opportunities to leave it. This finding may also indicate the role of social networking in reinforcing patriarchal norms and maintaining a form of tolerance towards intimate partner violence. For example, a study conducted in Tanzania demonstrated that gender norms within the peer network influenced men’s perpetration of intimate partner violence.^33^ The influence of gender norms on men’s behaviours and attitudes was found to rely on different processes, including the internalization of prevailing norms or the fact that men felt a form of pressure to conform to these norms. Similar mechanisms may explain the link between social networking and intimate partner violence in Ghana, Pakistan, and South Sudan, three countries with different sociocultural settings, but characterized by solid patriarchal norms and history of male dominance over women.^34–36^

Aside from these main findings, the bivariate analysis results showed that working women were at higher risk of experiencing intimate partner violence and family member violence, confirming the results of previous research among ever-married women in India.^37^ This evidence is worth mentioning as it adds to the current debate about economic empowerment and violence against women and informs on the need for future interventions targeting this population.

## Strengths and limitations

This study has some strengths, including a large number of participants, the representativeness of the sample, and the fact that three forms of violence were analysed and compared. However, some limitations should be mentioned. First, the community cohesion score may not fully capture the complexity of the construct since only two sub-dimensions (trust, mutual tolerance) were included in our model. Although minimalistic, this measure was built based on existing literature and tested through exploratory and confirmatory factor analyses, ensuring a high construct validity. Second, measures of community cohesion were derived from women’s responses only. Including the perception of men also might have led to different, perhaps more representative aggregated scores. These two limitations are inherent in secondary data analysis, where data were not collected to address the specific research question.^38^ Finally, advanced comparisons between Ghana, Pakistan, and South Sudan might have highlighted different internal dynamics. However, the high number of missing values regarding women’s experience of violence did not allow us to conduct relevant country-specific analyses.

## Conclusion

In conclusion, we found that women living in communities with high levels of community cohesion had lower risks of experiencing public spaces violence in Ghana, Pakistan, and South Sudan. At the same time, there is little or no evidence of a protective effect of community cohesion against family member violence and intimate partner violence. While violence against women remains exceptionally high in low- and middle-income countries, interventions fostering social cohesion within the community may reduce violence in its most visible forms. However, such strategies should be complemented by interventions focusing on individual- and household-level predictors to increase efficacy and fully encompass all the forms of violence targeting women. Notably, future programmes in low- and middle-income countries should incorporate strategies that focus on gender norms transformation and target individuals at higher risks of violence, such as working women. Another notable conclusion is that addressing violence in intimate partner relationships requires making the issue visible as one of collective responsibility.

## Supplemental Material

sj-docx-1-whe-10.1177_17455057221123998 – Supplemental material for Community cohesion and violence against women in Ghana, Pakistan, and South Sudan: A secondary data analysis Click here for additional data file. Supplemental material, sj-docx-1-whe-10.1177_17455057221123998 for Community cohesion and violence against women in Ghana, Pakistan, and South Sudan: A secondary data analysis by Sébastien Poix, Nuha Ibrahim, Stacey Scriver, Srinivas Raghavendra, Nata Duvvury and Khalifa Elmusharaf in Women’s Health

sj-png-2-whe-10.1177_17455057221123998 – Supplemental material for Community cohesion and violence against women in Ghana, Pakistan, and South Sudan: A secondary data analysis Click here for additional data file. Supplemental material, sj-png-2-whe-10.1177_17455057221123998 for Community cohesion and violence against women in Ghana, Pakistan, and South Sudan: A secondary data analysis by Sébastien Poix, Nuha Ibrahim, Stacey Scriver, Srinivas Raghavendra, Nata Duvvury and Khalifa Elmusharaf in Women’s Health

sj-png-3-whe-10.1177_17455057221123998 – Supplemental material for Community cohesion and violence against women in Ghana, Pakistan, and South Sudan: A secondary data analysis Click here for additional data file. Supplemental material, sj-png-3-whe-10.1177_17455057221123998 for Community cohesion and violence against women in Ghana, Pakistan, and South Sudan: A secondary data analysis by Sébastien Poix, Nuha Ibrahim, Stacey Scriver, Srinivas Raghavendra, Nata Duvvury and Khalifa Elmusharaf in Women’s Health

sj-png-4-whe-10.1177_17455057221123998 – Supplemental material for Community cohesion and violence against women in Ghana, Pakistan, and South Sudan: A secondary data analysis Click here for additional data file. Supplemental material, sj-png-4-whe-10.1177_17455057221123998 for Community cohesion and violence against women in Ghana, Pakistan, and South Sudan: A secondary data analysis by Sébastien Poix, Nuha Ibrahim, Stacey Scriver, Srinivas Raghavendra, Nata Duvvury and Khalifa Elmusharaf in Women’s Health

sj-png-5-whe-10.1177_17455057221123998 – Supplemental material for Community cohesion and violence against women in Ghana, Pakistan, and South Sudan: A secondary data analysis Click here for additional data file. Supplemental material, sj-png-5-whe-10.1177_17455057221123998 for Community cohesion and violence against women in Ghana, Pakistan, and South Sudan: A secondary data analysis by Sébastien Poix, Nuha Ibrahim, Stacey Scriver, Srinivas Raghavendra, Nata Duvvury and Khalifa Elmusharaf in Women’s Health

sj-png-6-whe-10.1177_17455057221123998 – Supplemental material for Community cohesion and violence against women in Ghana, Pakistan, and South Sudan: A secondary data analysis Click here for additional data file. Supplemental material, sj-png-6-whe-10.1177_17455057221123998 for Community cohesion and violence against women in Ghana, Pakistan, and South Sudan: A secondary data analysis by Sébastien Poix, Nuha Ibrahim, Stacey Scriver, Srinivas Raghavendra, Nata Duvvury and Khalifa Elmusharaf in Women’s Health

sj-png-7-whe-10.1177_17455057221123998 – Supplemental material for Community cohesion and violence against women in Ghana, Pakistan, and South Sudan: A secondary data analysis Click here for additional data file. Supplemental material, sj-png-7-whe-10.1177_17455057221123998 for Community cohesion and violence against women in Ghana, Pakistan, and South Sudan: A secondary data analysis by Sébastien Poix, Nuha Ibrahim, Stacey Scriver, Srinivas Raghavendra, Nata Duvvury and Khalifa Elmusharaf in Women’s Health

## Declarations

**Ethics approval and consent to participate:** None.

**Consent for publication:** None.

**Availability of data and materials:** The dataset analysed for the current study is available in the Harvard Dataverse repository, https://doi.org/10.7910/DVN/RU1X7W.
