# Supplementary material for: Community cohesion and violence against women in Ghana, Pakistan, and South Sudan: A secondary data analysis
Source: Womens Health (Lond). 2022 Sep 23;18:17455057221123998. doi: 10.1177/17455057221123998 (PMC9511548; doi:10.1177/17455057221123998)
Supplement: sj-docx-1-whe-10.1177_17455057221123998 – Supplemental material for Community cohesion and violence against women in Ghana, Pakistan, and South Sudan: A secondary data analysis [file sj-docx-1-whe-10.1177_17455057221123998.docx]

**Supplemental Materials for Community cohesion and violence against women and girls in Ghana, Pakistan, and South Sudan: a secondary data analysis.**

1. File: Supplemental_Material_1.png

Title: Confirmatory factor analysis (CFA) model for social networking

1. File: Supplemental_Material_2.png

Title: Exploratory factor analysis factor loadings for the community cohesion score (Structure Matrix)

1. File: Supplemental_Material_3.png

Title: Indices of fit model (Community Cohesion Score)

1. File: Supplemental_Material_4.png

Title: Exploratory factor analysis factor loadings for the social networking score (Structure Matrix)

1. File: Supplemental_Material_5

Title: Indices of fit model (Social Networking Score)

1. File: Supplemental_Material_6

Title: Women and Girl’s Questionnaire. Final Version: 29/01/16
