# Supplementary figures and images for: Community cohesion and violence against women in Ghana, Pakistan, and South Sudan: A secondary data analysis
Source: Womens Health (Lond). 2022 Sep 23;18:17455057221123998. doi: 10.1177/17455057221123998 (PMC9511548; doi:10.1177/17455057221123998)

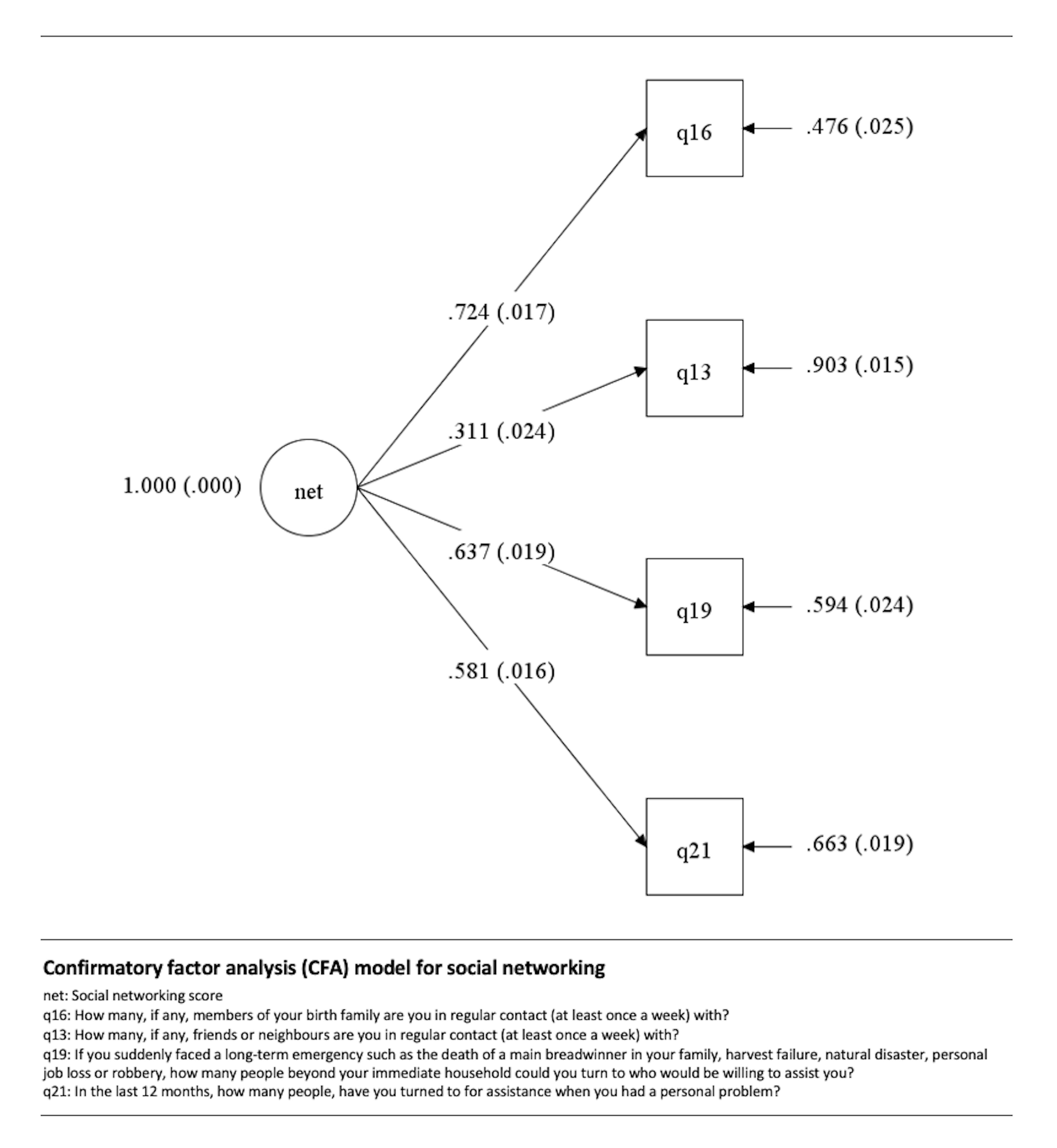

Supplement: sj-png-2-whe-10.1177_17455057221123998 – Supplemental material for Community cohesion and violence against women in Ghana, Pakistan, and South Sudan: A secondary data analysis [file sj-png-2-whe-10.1177_17455057221123998.png]

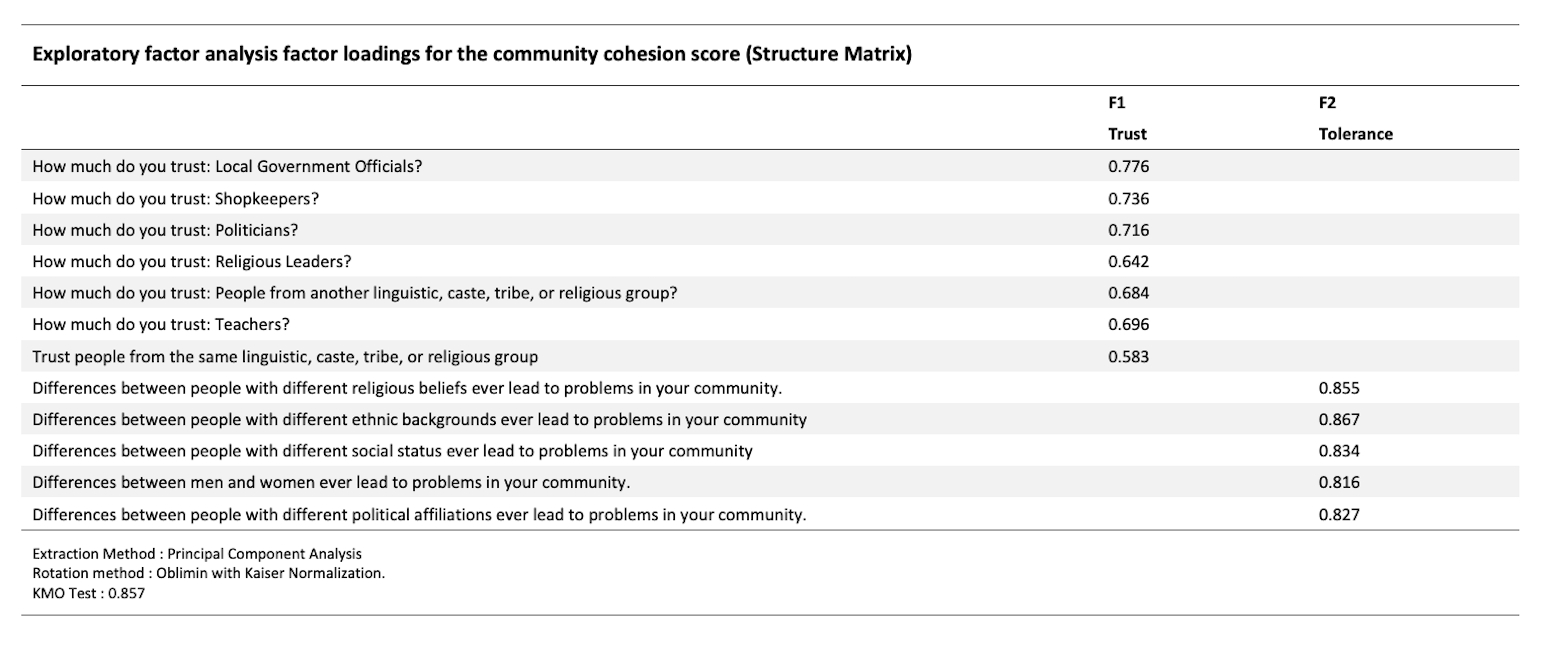

Supplement: sj-png-3-whe-10.1177_17455057221123998 – Supplemental material for Community cohesion and violence against women in Ghana, Pakistan, and South Sudan: A secondary data analysis [file sj-png-3-whe-10.1177_17455057221123998.png]

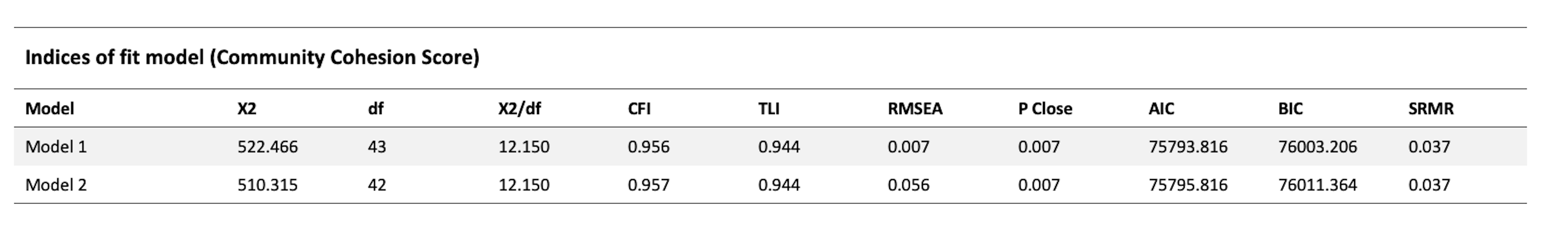

Supplement: sj-png-4-whe-10.1177_17455057221123998 – Supplemental material for Community cohesion and violence against women in Ghana, Pakistan, and South Sudan: A secondary data analysis [file sj-png-4-whe-10.1177_17455057221123998.png]

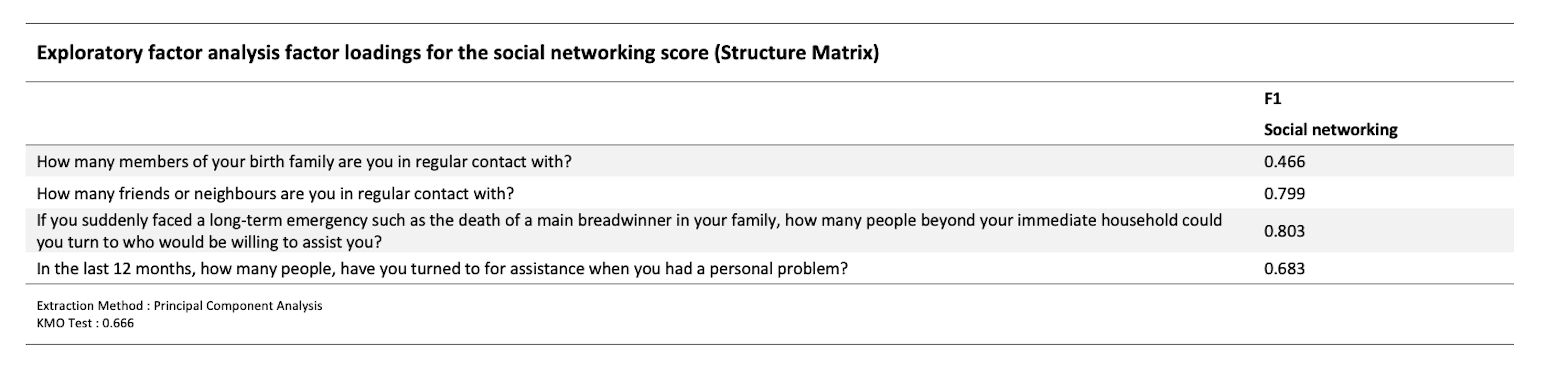

Supplement: sj-png-5-whe-10.1177_17455057221123998 – Supplemental material for Community cohesion and violence against women in Ghana, Pakistan, and South Sudan: A secondary data analysis [file sj-png-5-whe-10.1177_17455057221123998.png]

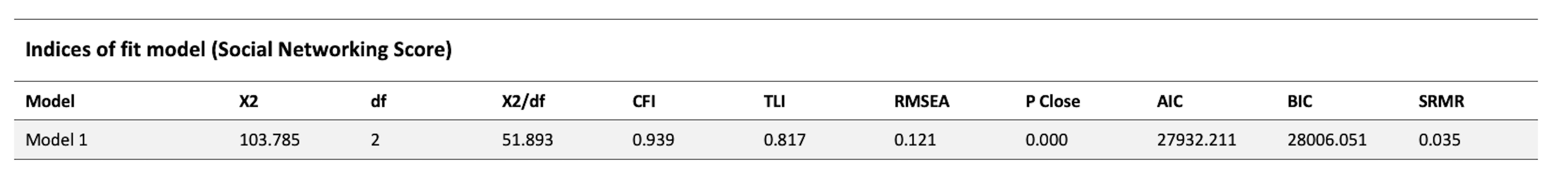

Supplement: sj-png-6-whe-10.1177_17455057221123998 – Supplemental material for Community cohesion and violence against women in Ghana, Pakistan, and South Sudan: A secondary data analysis [file sj-png-6-whe-10.1177_17455057221123998.png]
