# Supplementary material for: Community cohesion and violence against women in Ghana, Pakistan, and South Sudan: A secondary data analysis
Source: Womens Health (Lond). 2022 Sep 23;18:17455057221123998. doi: 10.1177/17455057221123998 (PMC9511548; doi:10.1177/17455057221123998)
Supplement: sj-png-7-whe-10.1177_17455057221123998 – Supplemental material for Community cohesion and violence against women in Ghana, Pakistan, and South Sudan: A secondary data analysis [file sj-png-7-whe-10.1177_17455057221123998.docx]

| IPSOS/ NUIG/ ICRW/ DFID 14-029624-01 |  |  |  |
| --- | --- | --- | --- |


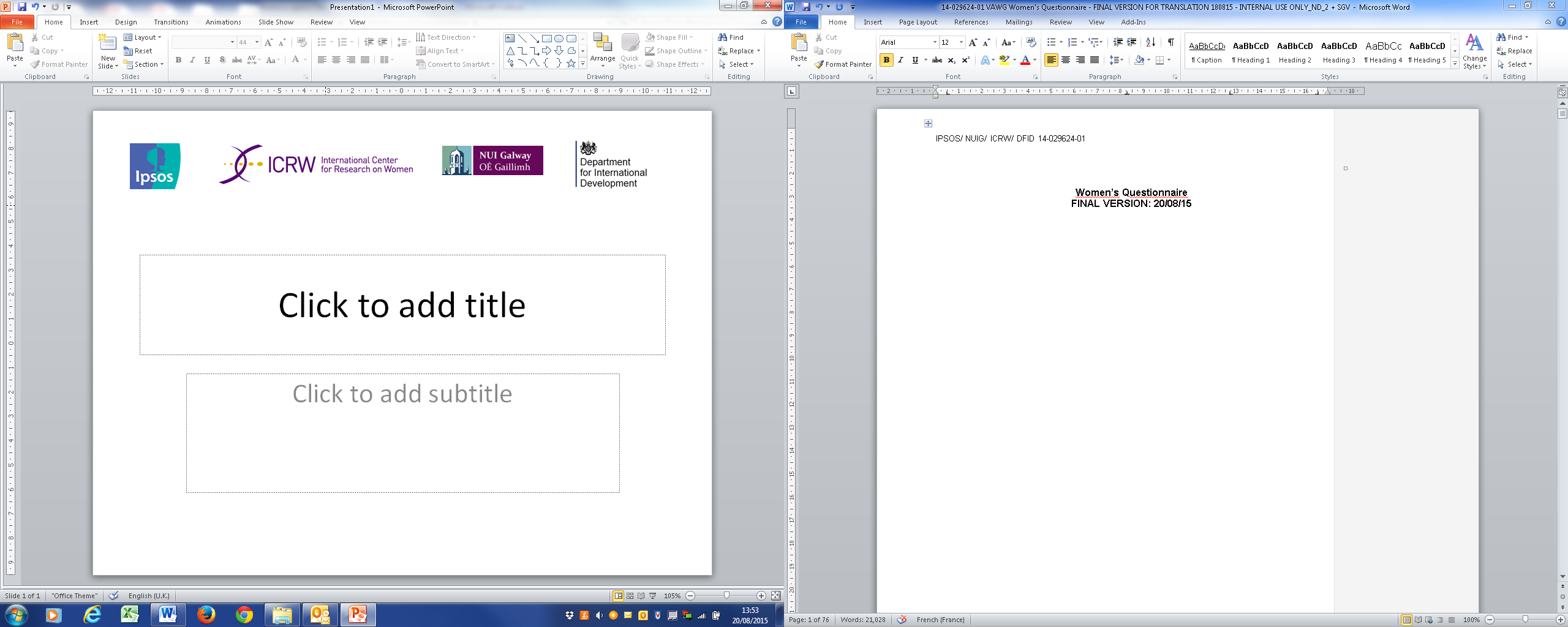


**(LETTER TO BE SEPARATE FROM QUESTIONNAIRE)**

#### **Important survey about women and girl’s health and life experiences**

<<Date>> Reference number:___________

Dear Resident,

**Re: Important survey about issues affecting woman and girls in <<<ADD COUNTRY>>>**

My name is …… from <<<ADD ORGANISATION>>> and we are carrying out a survey to learn about women and girl’s health and life experiences in <<ADD COUNTRY>>.

**The aim of the survey** is to better understand the issues that affect women and girls with respect to their wellbeing in <<ADD COUNTRY>>. The survey interview will take around **45 minutes** (60 minutes in South Sudan – DELETE AS APPROPRIATE) to complete. The results will be used to inform policies in your country that can improve the lives of women and girls. A number of organisations will be analysing the survey findings, including the National University of Ireland at Galway, Ipsos MORI in the UK, and the International Center for Research on Women (ICRW) based in Washington DC.

**The survey questions** are adapted from previous surveys on women and girl’s health and life experiences undertaken by the following well known institutions: World Health Organisation, Macro International, International Center for Research on Women, and National University of Ireland, Galway. The questions in this survey have been tested in different countries such as Bangladesh, Brazil, Cambodia, Cameroon, Egypt, India, Morocco, South Africa, Tanzania, Thailand, Uganda, and Vietnam.

**Why did we choose you?** Your home has been selected at random for inclusion in the survey. To ensure our results are accurate, we rely on the voluntary co-operation of people in selected homes – such as yours. We would like to interview one woman or girl in your household who is aged between 18 and 60 years of age. If there is more than one woman or girl living at this address who is aged between 18 and 60, the enumerator will select one person at random from the household to be interviewed.

**Please be assured that any contributions to this survey will be kept completely anonymous and treated in the strictest confidence.** You are *not* obliged to take part in this survey. However, we do hope you will be willing to help us by participating in this important research. If you would like any more information about the survey please contact [NAME] at <<<Ipsos Ghana/ Ipsos Pakistan/ Tango Consult>>> on [TELNO]. You can also email [EMAIL], quoting your name and the reference number that appears above.

Thank you very much for your help. The topic of this survey is vitally important to women and girls lives and we hope that you can help us to explore it further.

Yours faithfully

<<Signature>>

------------------------

Name

Research Director

<<<Ipsos Ghana/ Ipsos Pakistan/ Tango Consult>>>

| IPSOS/ NUIG/ ICRW/ DFID 14-029624-01 | SERIAL  NUMBER |  | |  |  |  | |  |  | |
| --- | --- | --- | --- | --- | --- | --- | --- | --- | --- | --- |
|  | | |  | | | |  | |  |  |

**Women and Girl’s Questionnaire**

#### **FINAL VERSION: 29/01/16**

| INTERVIEWER RECORD START TIME |  |  |  |  |  |  |  |  |
| --- | --- | --- | --- | --- | --- | --- | --- | --- |
|  | Hours | | | | Mins | | | |

| ABOUT YOU |
| --- |

**I would first like to ask a few questions about you.**

| Q | | **How old are you?**  SINGLE CODE ONLY | | | | |  | |
| --- | --- | --- | --- | --- | --- | --- | --- | --- |
|  |  | |  |  |  |  | |  |
|  |  | | CODE ‘1’ AND WRITE IN AGE | 1  ☐☐ |  |  | |  |
|  |  | | Don’t know | 98 |  |  | |  |

| Q | | | **Have you ever attended school?** IF YES ASK **And can I just check.** **Are you attending school or another educational institution at the moment or did you attend in the past?**  SINGLE CODE ONLY | | | |  | |
| --- | --- | --- | --- | --- | --- | --- | --- | --- |
|  |  |  | |  |  |  | |  |
|  |  | Yes – I am attending school / educational institution at the moment | | 1 | go to q5 |  | |  |
|  |  | Yes – I have attended school / educational institution in the past, but not now | | 2 | go to q4 |  | |  |
|  |  | No – I have never attended school / educational institution | | 3 | ASK Q3 |  | |  |
|  |  | Don’t know | | 98 | go to q6 |  | |  |

ASK Q3 IF THE WOMAN OR GIRL HAS NEVER ATTENDED SCHOOL. THIS IS CODE 3 AT Q2. OTHERS GO TO Q4 OR Q5 OR Q6 DEPENDING ON ANSWER TO Q2.

| Q | | SHOWCARD 1 **Please tell me why you have never attended school or an educational institution? Read out the statement or statements from the card that apply.**  MULTICODE OKAY  INTERVIEWER NOTE: IF THE WOMEN CANNOT READ, PLEASE READ OUT THE OPTIONS. THIS WILL APPLY TO ALL QUESTIONS WITH SHOWCARDS. | | | | |  | |
| --- | --- | --- | --- | --- | --- | --- | --- | --- |
|  |  | |  |  |  |  | |  |
|  |  | | My parents could not afford to send me to school | 1 |  |  | |  |
|  |  | | My parents did not want to send me to school | 2 |  |  | |  |
|  |  | | There was no education available | 3 |  |  | |  |
|  |  | | Other reason – Interviewer find out & write in: | 4 |  |  | |  |
|  |  | | . None of these | 5 |  |  | |  |
|  |  | | Don’t know | 98 |  |  | |  |

ASK Q4 IF THE WOMAN OR GIRL HAS ATTENDED SCHOOL IN THE PAST. THIS IS CODE 2 AT Q2. OTHERS GO TO Q5.

| Q | | SHOWCARD 2 **Why do you no longer attend school? Read out the statement or statements from this card that apply.**  MULTICODE OKAY | | | | |  | |
| --- | --- | --- | --- | --- | --- | --- | --- | --- |
|  |  | |  |  |  |  | |  |
|  |  | | I had accomplished my educational goals | 1 |  |  | |  |
|  |  | | I decided to find work | 2 |  |  | |  |
|  |  | | I left because of the financial cost | 3 |  |  | |  |
|  |  | | I had to look after family or other relatives | 4 |  |  | |  |
|  |  | | My parents forced me to leave school | 5 |  |  | |  |
|  |  | | I was forced to leave school by my husband or partner | 6 |  |  | |  |
|  |  | | Someone else forced me to leave school - INTERVIEWER: FIND OUT WHO & WRITE IN | 7 |  |  | |  |
|  |  | | It was too dangerous to attend | 8 |  |  | |  |
|  |  | | I had to leave because of marriage | 9 |  |  | |  |
|  |  | | Other reason - INTERVIEWER FIND OUT & WRITE IN: | 10 |  |  | |  |
|  |  | | None of these reasons | 11 |  |  | |  |
|  |  | | Don’t know | 98 |  |  | |  |

ASK Q5 IF THE WOMAN OR GIRL IS ATTENDING SCHOOL NOW OR HAS EVER ATTENDED SCHOOL. THIS IS CODE 1 OR CODE 2 AT Q2. OTHERS GO TO Q6.

| Q | | SHOWCARD 3 **Please look at this card and tell me the highest level of education you have obtained?**  SINGLE CODE ONLY | | | | |  | |
| --- | --- | --- | --- | --- | --- | --- | --- | --- |
|  |  | |  |  |  |  | |  |
|  |  | | Pre-school | 1 |  |  | |  |
|  |  | | Primary | 2 |  |  | |  |
|  |  | | Middle/Junior Secondary School/Junior High School | 3 |  |  | |  |
|  |  | | Secondary/Senior Secondary School /Senior High School | 4 |  |  | |  |
|  |  | | Technical/Vocational | 5 |  |  | |  |
|  |  | | Higher/University | 6 |  |  | |  |
|  |  | | Don’t know | 98 |  |  | |  |

ASK ALL

| Q | | **How long have you lived in this house?**  SINGLE CODE ONLY | | | | |  | |
| --- | --- | --- | --- | --- | --- | --- | --- | --- |
|  |  | |  |  |  |  | |  |
|  |  | | Less than 1 year | 1 |  |  | |  |
|  |  | | Between 1 and 2 years | 2 |  |  | |  |
|  |  | | Over 2 and up to 5 years | 3 |  |  | |  |
|  |  | | Over 5 and up to 10 years | 4 |  |  | |  |
|  |  | | More than 10 years | 5 |  |  | |  |
|  |  | | Don’t know | 98 |  |  | |  |

| Q | | **And how long have you lived in this area?**  SINGLE CODE ONLY | | | | |  | |
| --- | --- | --- | --- | --- | --- | --- | --- | --- |
|  |  | |  |  |  |  | |  |
|  |  | | Less than 1 year | 1 |  |  | |  |
|  |  | | Between 1 and 2 years | 2 |  |  | |  |
|  |  | | Over 2 and up to 5 years | 3 |  |  | |  |
|  |  | | Over 5 and up to 10 years | 4 |  |  | |  |
|  |  | | More than 10 years | 5 |  |  | |  |
|  |  | | Don’t know | 98 |  |  | |  |

| Q8. | | **Do you live with your birth parents or any of your birth relatives?**  SINGLE CODE ONLY | | | | |
| --- | --- | --- | --- | --- | --- | --- |
|  |  | |  |  |  |  |
|  |  | | Yes | 1 |  |  |
|  |  | | No | 2 | ASK Q9 |  |
|  |  | | Prefer not to say | 97 |  |  |
|  |  | | Not applicable as have no known family | 99 | GO TO Q16 |  |

ASK Q9 IF THE WOMAN OOR GIRL HAS FAMILY - CODES 1, 2 OR 97 AT Q8.

| Q9. | | **Do any of your family of birth (that is your mother, father, brother or sister) live in this area?**  SINGLE CODE ONLY | | | | |  | |
| --- | --- | --- | --- | --- | --- | --- | --- | --- |
|  |  | |  |  |  |  | |  |
|  |  | | Yes | 1 | GO TO Q11 |  | |  |
|  |  | | No | 2 | ASK Q10 |  | |  |
|  |  | | Don’t know | 98 | GO TO Q11 |  | |  |

ASK Q10 IF CODE 2 AT Q9. OTHERS GO TO Q11.

| Q10. | | SHOWCARD 4 **As they don’t live in this area, how often, if at all, do you talk, either in person or by telephone, to a member of your family of birth?**  SINGLE CODE ONLY | | | | |  | |
| --- | --- | --- | --- | --- | --- | --- | --- | --- |
|  |  | |  |  |  |  | |  |
|  |  | | At least once a week | 1 | go to q12 |  | |  |
|  |  | | A few times a month | 2 |  |  | |  |
|  |  | | A few times a year | 3 | ASK Q11 |  | |  |
|  |  | | Once a year or less often | 4 |  |  | |  |
|  |  | | Never | 5 |  |  | |  |
|  |  | | Don’t know | 98 | GO TO Q12 |  | |  |
|  |  | |  |  |  |  | |  |

ASK Q11 IF THE WOMAN OR GIRL TALKS TO MEMBERS OF HER BIRTH FAMILY A FEW TIMES A YEAR OR LESS. THAT IS CODE ‘3’ OR ‘4’ OR ‘5’ AT Q10. OTHERS GO TO Q12.

| Q11 | | SHOWCARD 5 **Please tell me why you do not talk to your family of birth more often? Read out the statement from this card that best applies.**  SINGLE CODE ONLY | | | | |  | |
| --- | --- | --- | --- | --- | --- | --- | --- | --- |
|  |  | |  |  |  |  | |  |
|  |  | | They live too far away for me to visit them | 1 |  |  | |  |
|  |  | | I choose not to talk to them more often | 2 |  |  | |  |
|  |  | | My husband or partner does not like me to talk to them | 3 |  |  | |  |
|  |  | | My husband or partner does not let me talk to them | 4 |  |  | |  |
|  |  | | I have fallen out with them | 5 |  |  | |  |
|  |  | | I do not have any birth family members still alive | 6 |  |  | |  |
|  |  | | I do not have access to a phone | 7 |  |  | |  |
|  |  | | Other reason - INTERVIEWER FIND OUT & WRITE IN | 9 |  |  | |  |
|  |  | | None of these | 10 |  |  | |  |
|  |  | | Prefer not to say | 97 |  |  | |  |
|  |  | | Don’t know | 98 |  |  | |  |

ASK ALL WITH KNOWN FAMILY (CODE ‘1’ OR ‘2’ OR ‘97’ AT Q8). OTHERS GO TO Q16

| Q12 | | **If you needed help or had a problem, could you count on members of your birth family for support?**  SINGLE CODE ONLY | | | | |  | |
| --- | --- | --- | --- | --- | --- | --- | --- | --- |
|  |  | |  |  |  |  | |  |
|  |  | | Yes | 1 |  |  | |  |
|  |  | | No | 2 |  |  | |  |
|  |  | | Don’t know | 98 |  |  | |  |
|  |  | | Not applicable | 99 |  |  | |  |

| SOCIAL NETWORKS |
| --- |

| Q13. | | **How many, if any, members of your birth family are you in regular contact with? By regular contact I mean at least once a week.** SINGLE CODE ONLY. | | | | |  | |
| --- | --- | --- | --- | --- | --- | --- | --- | --- |
|  |  | |  |  |  |  | |  |
|  |  | | CODE “1” AND WRITE IN THE NUMBER | 1  ☐☐ |  |  | |  |
|  |  | | None | 2 |  |  | |  |
|  |  | | Don’t know | 98 |  |  | |  |

| Q14. | | **How many, if any, of your birth family would you share your personal problems with?**  SINGLE CODE ONLY. | | | | |  | |
| --- | --- | --- | --- | --- | --- | --- | --- | --- |
|  |  | |  |  |  |  | |  |
|  |  | | CODE “1” AND WRITE IN THE NUMBER | 1  ☐☐ |  |  | |  |
|  |  | | None | 2 |  |  | |  |
|  |  | | Don’t know | 98 |  |  | |  |

| Q15. | | **And, how many, if any, of your birth family would you ask a favour of? For instance, to look after your children or lend you items or small amounts of money?**  SINGLE CODE ONLY. | | | | |  | |
| --- | --- | --- | --- | --- | --- | --- | --- | --- |
|  |  | |  |  |  |  | |  |
|  |  | | CODE “1” AND WRITE IN THE NUMBER | 1  ☐☐ |  |  | |  |
|  |  | | None | 2 |  |  | |  |
|  |  | | Don’t know | 98 |  |  | |  |

ASK ALL

| Q16. | | **How many friends or neighbours are you in regular contact with? By regular contact I mean at least once a week.**  SINGLE CODE ONLY. | | | | |  | |
| --- | --- | --- | --- | --- | --- | --- | --- | --- |
|  |  | |  |  |  |  | |  |
|  |  | | CODE “1” AND WRITE IN THE NUMBER | 1  ☐☐ | ASK Q17 |  | |  |
|  |  | | None | 2 | GO TO Q19 |  | |  |
|  |  | | Don’t know | 98 |  |  | |  |

ASK Q17 IF THE WOMAN OR GIRL HAS 1 OR MORE FRIENDS OR NEIGHBOURS (CODE ‘1’ AT Q16).

OTHERS GO TO Q19.

| Q17. | | **How many of these friends or neighbours, if any, would you share your personal problems with?** SINGLE CODE ONLY. | | | | |  | |
| --- | --- | --- | --- | --- | --- | --- | --- | --- |
|  |  | |  |  |  |  | |  |
|  |  | | CODE “1” AND WRITE IN THE NUMBER | 1  ☐☐ |  |  | |  |
|  |  | | None | 2 |  |  | |  |
|  |  | | Don’t know | 98 |  |  | |  |

| Q18. | | **And, how many, if any close friends or neighbours would you ask a favour of? For instance, to look after your children or lend you items or small amounts of money?** SINGLE CODE ONLY. | | | | |  | |
| --- | --- | --- | --- | --- | --- | --- | --- | --- |
|  |  | |  |  |  |  | |  |
|  |  | | CODE “1” AND WRITE IN THE NUMBER | 1  ☐☐ |  |  | |  |
|  |  | | None | 2 |  |  | |  |
|  |  | | Don’t know | 98 |  |  | |  |

ASK ALL

| Q19. | | **If you suddenly faced a long-term emergency such as the death of a main breadwinner in your family or** [RURAL: **harvest failure, or natural disaster, or robbery** URBAN: **personal** **job loss**, **or natural disaster, or robbery] how many people beyond your immediate household could you turn to who would be willing to assist you?** SINGLE CODE ONLY. | | | | |  | |
| --- | --- | --- | --- | --- | --- | --- | --- | --- |
|  |  | |  |  |  |  | |  |
|  |  | | CODE “1” AND WRITE IN THE NUMBER | 1  ☐☐ | ask q20 |  | |  |
|  |  | | None | 2 | go to q21 |  | |  |
|  |  | | Don’t know | 98 |  |  | |  |

ASK Q20 IF THE WOMAN OR GIRL HAS 1 OR MORE PEOPLE TO ASSIST THEM (CODE 1 AT Q19).

OTHERS GO TO Q21.

| Q20. | | **And of those people, how many, if any, do you think would be able to help you?**  SINGLE CODE ONLY. | | | | |  | |
| --- | --- | --- | --- | --- | --- | --- | --- | --- |
|  |  | |  |  |  |  | |  |
|  |  | | CODE “1” AND WRITE IN THE NUMBER | 1  ☐☐ |  |  | |  |
|  |  | | None | 2 |  |  | |  |
|  |  | | Don’t know | 98 |  |  | |  |

ASK ALL

| Q21. | | **In the last 12 months, how many people, have you turned to for assistance when you had a personal problem?** **Assistance can be financial or in-kind support such as looking after your children, intervening with family if needed, help with home production or in the market selling your goods.**  SINGLE CODE ONLY. | | | | |  | |
| --- | --- | --- | --- | --- | --- | --- | --- | --- |
|  |  | |  |  |  |  | |  |
|  |  | | CODE “1” AND WRITE IN THE NUMBER | 1  ☐☐ | ASK Q22 |  | |  |
|  |  | | None | 2 | GO TO Q23 |  | |  |
|  |  | | Don’t know | 98 |  |  | |  |

ASK Q22 IF CODE 1 AT Q21 (HAS HAD ASSISTANCE WITH A PERSONAL PROBLEM).

OTHERS GO TO Q23 IN THE NEXT SECTION.

| Q22. | | **Of the people you have turned to in the last 12 months for assistance with a personal problem, would you say these people have more money, less money or the same money as your family?**  SINGLE CODE ONLY | | | | |  | |
| --- | --- | --- | --- | --- | --- | --- | --- | --- |
|  |  | |  |  |  |  | |  |
|  |  | | More money than my family | 1 |  |  | |  |
|  |  | | Less money than my family | 2 |  |  | |  |
|  |  | | The same money as my family | 3 |  |  | |  |
|  |  | | Don’t know | 98 |  |  | |  |

ASK ALL

| THE LOCAL COMMUNITY |
| --- |

| Q23. | | SHOWCARD 6 **To what extent, do you agree or disagree with each of the following statements about your local community? Think about your local community as being your immediate neighbourhood.** READ OUT A TO F. SINGLE CODE FOR EACH | | | | | | |  | |
| --- | --- | --- | --- | --- | --- | --- | --- | --- | --- | --- |
|  |  | |  | Strongly agree | Tend to agree | Neither agree nor disagree | Tend to disagree | Strongly disagree | | Don’t know |
|  | a) | | People in my local community get along well together | 1 | 2 | 3 | 4 | 5 | | 98 |
|  | b) | | If there was a street fight in my community, people would do something to stop it | 1 | 2 | 3 | 4 | 5 | | 98 |
|  | c) | | Neighbours here know each other well | 1 | 2 | 3 | 4 | 5 | | 98 |
|  | d) | | People here generally trust one another in matters of lending and borrowing things | 1 | 2 | 3 | 4 | 5 | | 98 |
|  | e) | | If a member of my family fell ill or had an accident, neighbours here would help me | 1 | 2 | 3 | 4 | 5 | | 98 |
|  | f) | | I like living in my local community | 1 | 2 | 3 | 4 | 5 | | 98 |

| Q24. | | SHOWCARD 7 **I would now like to ask you about how much you trust different groups of people who live and work in your local community. By trust I mean that they are reliable and dependable.**  **Firstly, tell me how much you trust…**  READ OUT A TO J. SINGLE CODE FOR EACH | | | | | | |  | |
| --- | --- | --- | --- | --- | --- | --- | --- | --- | --- | --- |
|  |  | |  | A great deal | A fair amount | Not very much | Not at all | Prefer not to say | | Don’t know |
|  | a) | | …people from the same linguistic, caste, tribe or religious group as you? | 1 | 2 | 3 | 4 | 97 | | 98 |
|  | b) | | …people from another linguistic, caste, tribe or religious group? | 1 | 2 | 3 | 4 | 97 | | 98 |
|  | c) | | …shopkeepers? | 1 | 2 | 3 | 4 | 97 | | 98 |
|  | d) | | …local government officials? | 1 | 2 | 3 | 4 | 97 | | 98 |
|  | e) | | …central government officials? | 1 | 2 | 3 | 4 | 97 | | 98 |
|  | f) | | …teachers? | 1 | 2 | 3 | 4 | 97 | | 98 |
|  | g) | | …nurses and doctors? | 1 | 2 | 3 | 4 | 97 | | 98 |
|  | h) | | …politicians? | 1 | 2 | 3 | 4 | 97 | | 98 |
|  | i) | | …religious leaders? | 1 | 2 | 3 | 4 | 97 | | 98 |
|  | j) | | …strangers? | 1 | 2 | 3 | 4 | 97 | | 98 |

‘CASTE’ SHOULD ONLY BE INCLUDED IN PAKISTAN.

| ACCESS TO SERVICES |
| --- |

| Q25a. | | **Have you or any member of your family ever had difficulty in accessing any of the following services…?**  READ OUT A TO K. SINGLE CODE FOR EACH | | | | | | |  | |
| --- | --- | --- | --- | --- | --- | --- | --- | --- | --- | --- |
|  |  | |  | Yes | No | Don’t know | Not applicable |  | |  |
|  | a) | | Education services and schools | 1 | 2 | 98 | 99 |  | |  |
|  | b) | | Health services and clinics | 1 | 2 | 98 | 99 |  | |  |
|  | c) | | Job training or employment services | 1 | 2 | 98 | 99 |  | |  |
|  | d) | | Credit and finance services | 1 | 2 | 98 | 99 |  | |  |
|  | e) | | Transportation services | 1 | 2 | 98 | 99 |  | |  |
|  | f) | | Water services | 1 | 2 | 98 | 99 |  | |  |
|  | g) | | Sanitation services |  |  |  |  |  | |  |
|  | h) | | Electricity and gas services | 1 | 2 | 98 | 99 |  | |  |
|  | i) | | Security services | 1 | 2 | 98 | 99 |  | |  |
|  | j) | | Police services |  |  |  |  |  | |  |
|  | k) | | Agricultural extension (this is a service providing information on crops, cooking and food etc) | 1 | 2 | 98 | 99 |  | |  |
|  |  | |  |  |  |  |  |  | |  |

FOR GHANA – INCLUDE THE TEXT IN BRACKETS AT Q25A (K) AND AT Q26K.

Q25b. Are there any other services that you or a member of your family has ever had difficulty accessing?

INTERVIEWER: IF YES, WRITE IN:____________________________________________________

ASK Q26 FOR EACH SERVICE THE WOMAN OR GIRL OR A MEMBER OF HER FAMILY SAID YES TO AT Q25a. IF ALL SERVICES ARE CODED ‘2’ (N0) AT Q25a GO TO Q28 IN THE NEXT SECTION.

| Q26. | | SHOWCARD 8 **And thinking about the service or services that you or a member of your family had difficulty accessing. Do you think that other families within your community are more likely than you, less likely than you OR as likely as you to experience difficulties accessing the same services?**  READ OUT A TO K. SINGLE CODE FOR EACH | | | | | | |  | |
| --- | --- | --- | --- | --- | --- | --- | --- | --- | --- | --- |
|  |  | |  | More likely than me | Less likely than me | As likely as me | Don’t know |  | |  |
|  | a) | | Education services and schools | 1 | 2 | 3 | 98 |  | |  |
|  | b) | | Health services and clinics | 1 | 2 | 3 | 98 |  | |  |
|  | c) | | Job training or employment services | 1 | 2 | 3 | 98 |  | |  |
|  | d) | | Credit and finance services | 1 | 2 | 3 | 98 |  | |  |
|  | e) | | Transportation services | 1 | 2 | 3 | 98 |  | |  |
|  | f) | | Water services | 1 | 2 | 3 | 98 |  | |  |
|  | g) | | Sanitation services |  |  |  |  |  | |  |
|  | h) | | Electricity and gas | 1 | 2 | 3 | 98 |  | |  |
|  | i) | | Security |  |  |  |  |  | |  |
|  | j) | | Police services | 1 | 2 | 3 | 98 |  | |  |
|  | k) | | Agricultural extension (this is a service providing information on crops, cooking and food etc | 1 | 2 | 3 | 98 |  | |  |
|  | l) | | Other (SPECIFY ACCORDING TO RESPONDENT’S ANSWER AT Q25b) |  |  |  |  |  | |  |

ASK Q27 IF ONE OR MORE SERVICE CODED AS ‘2’ AT Q26. IF NO SERVICE CODED ‘2’ AT Q26, GO TO Q28 IN THE NEXT SECTION

| Q27. | | SHOWCARD 9 **Among households that have difficulty accessing services in your community, which, if any, of the following reasons on this card would explain these difficulties?**  MULTICODE OKAY | | | | | | |  | |
| --- | --- | --- | --- | --- | --- | --- | --- | --- | --- | --- |
|  |  | |  |  |  |  |  |  | |  |
|  | a) | | Their social status | 1 |  |  |  |  | |  |
|  | b) | | Sex of head of household (male or female) | 2 |  |  |  |  | |  |
|  | c) | | Their age (young or old) | 3 |  |  |  |  | |  |
|  | d) | | Their political party affiliation | 4 |  |  |  |  | |  |
|  | e) | | Their religion | 5 |  |  |  |  | |  |
|  | f) | | Their education | 6 |  |  |  |  | |  |
|  | g) | | Their financial or economic status | 7 |  |  |  |  | |  |
|  | h) | | Other (SPECIFY) | 8 |  |  |  |  | |  |
|  |  | | None of these | 9 |  |  |  |  | |  |
|  |  | | Don’t know | 98 |  |  |  |  | |  |

ASK ALL

| GOVERNANCE |
| --- |

| Q28. | | SHOWCARD 10 **To what extent do you agree or disagree that each of the following are managed well in your local community?**  READ OUT A TO F. SINGLE CODE FOR EACH | | | | | | |  | |
| --- | --- | --- | --- | --- | --- | --- | --- | --- | --- | --- |
|  |  | |  | Strongly agree | Tend to agree | Neither agree nor disagree | Tend to disagree | Strongly disagree | | Don’t know |
|  | a) | | Sanitation | 1 | 2 | 3 | 4 | 5 | | 98 |
|  | b) | | Maintenance of roads | 1 | 2 | 3 | 4 | 5 | | 98 |
|  | c) | | Access to communal lands | 1 | 2 | 3 | 4 | 5 | | 98 |
|  | d) | | Water | 1 | 2 | 3 | 4 | 4 | | 98 |
|  | e) | | Electricity | 1 | 2 | 3 | 4 | 5 | | 98 |
|  | f) | | Other (PROBE FULLY AND WRITE IN) | 1 | 2 | 3 | 4 | 5 | | 98 |

ASK Q29 IF THE WOMAN OR GIRL DISAGREES THAT CORRESPONDING SERVICE AT Q28 A-F IS MANAGED WELL (CODES 4 OR 5). OTHERS GO TO Q30.

| Q29 | | **What is the main reason you disagree that** (SERVICE FROM Q29A-Q29F) **is managed well in your local community?**  READ OUT EACH REASON. SINGLE CODE ONLY FOR EACH | | | | | | |  | |
| --- | --- | --- | --- | --- | --- | --- | --- | --- | --- | --- |
|  |  | |  | Q28A SANITA-TION | Q28B ROAD MAINTEN-ANCE | Q28C ACCESS TO COMM-UNAL LANDS | Q28D WATER | Q28E ELEC-RICITY | | Q28F OTHER |
|  | a) | | Corruption | 1 | 1 | 1 | 1 | 1 | | 1 |
|  | b) | | Lack of interest by decision-makers | 1 | 1 | 1 | 1 | 1 | | 1 |
|  | c) | | Lack of community influence in decision-making | 2 | 2 | 2 | 2 | 2 | | 2 |
|  | d) | | Lack of money | 3 | 3 | 3 | 3 | 3 | | 3 |
|  | e) | | Unequal access to public goods | 4 | 4 | 4 | 4 | 4 | | 4 |
|  | f) | | No local representative government | 5 | 5 | 5 | 5 | 5 | | 5 |
|  | g) | | Other (SPECIFY) | 6 | 6 | 6 | 6 | 6 | | 6 |
|  |  | | None of these | 7 | 7 | 7 | 7 | 7 | | 7 |
|  |  | | Don’t know | 98 | 98 | 98 | 98 | 98 | | 98 |

ASK ALL

| Q30a.  Q30b.  Q30c. | | SHOWCARD 11 **To what extent, if at all, do local government and local leaders take into account the concerns of local women and men when they make decisions that affect them?** SINGLE CODE FOR EACH  SHOWCARD 11 AGAIN **To what extent, if at all do local government and local leaders take into account the concerns of local men when they make decisions that affect them?**  SHOWCARD 11 AGAIN **And to what extent, if at all do local government and local leaders take into account the concerns of local women when they make decisions that affect them?** | | | | | | |  | |
| --- | --- | --- | --- | --- | --- | --- | --- | --- | --- | --- |
|  |  | |  | Q30a  Men and women | Q30b  Men | Q30c  Women |  |  | |  |
|  |  | | A great deal | 1 | 2 | 3 |  |  | |  |
|  |  | | A fair amount | 1 | 2 | 3 |  |  | |  |
|  |  | | Not very much | 1 | 2 | 3 |  |  | |  |
|  |  | | Not at all | 1 | 2 | 3 |  |  | |  |
|  |  | | Don’t know | 1 | 2 | 3 |  |  | |  |

| PARTICIPATION AND VOLUNTEERING |
| --- |

| Q31. | | SHOWCARD 12 **Which, if any, of the things on this card have you done in the last three years? Read out the statement or statements that apply.**  MULTICODE OK. | | | | |  | |
| --- | --- | --- | --- | --- | --- | --- | --- | --- |
|  |  | |  |  |  |  | |  |
|  |  | | Voted in an election or a referendum | 1 | ask q32 |  | |  |
|  |  | | Took part in a protest or march | 2 |  |  | |  |
|  |  | | Talked to neighbours about a problem or issue you had | 3 |  |  | |  |
|  |  | | Notified the police or local authorities in your community about a problem you had | 4 |  |  | |  |
|  |  | | Made a monetary or in-kind donation to a charitable organisation. In kind - something other than giving money such as clothes, food, medicines, etc. | 5 |  |  | |  |
|  |  | | Volunteered for a charitable organisation | 6 |  |  | |  |
|  |  | | Participated in a community group, club or society | 7 |  |  | |  |
|  |  | | None of these | 8 | go to q33 |  | |  |
|  |  | | Don’t know | 98 |  |  | |  |

ASK Q32 FOR EACH ACTIVITY DONE AT Q31. IF NO ACTIVITY DONE AT Q31 [CODE 98 OR 99] GO TO Q33.

| Q32. | | SHOWCARD 13 **Please tell me how often you** (ACTIVITY FROM Q31) **in the last three years?**  READ OUT FOR EACH ACTIVITY CODED AT Q31. SINGLE CODE FOR EACH | | | | | | |  | |
| --- | --- | --- | --- | --- | --- | --- | --- | --- | --- | --- |
|  |  | |  | Once or twice | Between 3 and 5 times | Between 6 and 10 times | More than 10 times | Prefer not to say | | Don’t know |
|  | a) | | Voted in an election or a referendum | 1 | 2 | 3 | 4 | 97 | | 98 |
|  | b) | | Took part in a protest or march | 1 | 2 | 3 | 4 | 97 | | 98 |
|  | c) | | Talked to neighbours about a problem or issue you had | 1 | 2 | 3 | 4 | 97 | | 98 |
|  | d) | | Notified the police or local authorities in your community about a problem you had | 1 | 2 | 3 | 4 | 97 | | 98 |
|  | e) | | Made a monetary or in-kind donation to a charitable organisation In kind - something other than giving money such as clothes, food, medicines, etc. | 1 | 2 | 3 | 4 | 97 | | 98 |
|  | f) | | Volunteered for a charitable organisation | 1 | 2 | 3 | 4 | 97 | | 98 |
|  | g) | | Participated in a community group, club or society | 1 | 2 | 3 | 4 | 97 | | 98 |

ASK Q33 IF CODE 98 OR 99 USED AT Q31. OTHERS GO TO Q35.

| Q33. | | SHOWCARD 14 **Please tell me why you have not participated in a community group, club or society in the last three years? Read out the statement or statements from this card that apply.** MULTICODE OKAY. | | | | |  | |
| --- | --- | --- | --- | --- | --- | --- | --- | --- |
|  |  | |  |  |  |  | |  |
|  |  | | I was prevented from participating | 1 | ASK Q34 |  | |  |
|  |  | | I had no interest | 2 |  |  | |  |
|  |  | | I did not think I had anything to contribute | 3 |  |  | |  |
|  |  | | I did not think I had anything to gain | 4 | GO TO Q35 |  | |  |
|  |  | | I did not have the right clothes | 5 |  |  | |  |
|  |  | | I did not feel I belonged | 6 |  |  | |  |
|  |  | | I did not have money for transport or fees | 7 |  |  | |  |
|  |  | | I did not know how to participate | 8 |  |  | |  |
|  |  | | None of these | 9 |  |  | |  |
|  |  | | Don’t know | 98 |  |  | |  |

ASK Q34 IF THE WOMAN OR GIRL WAS PREVENTED FROM PARTICIPATING IN A COMMUNITY GROUP,

CLUB OR SOCIETY (CODE 1 AT Q33). OTHERS GO TO Q35.

| Q34. | | SHOWCARD 15 **Who prevented you from taking part in a community group, club, or society? Read out the statement or statements from this card that apply.**  MULTICODE OKAY. | | | | |  | |
| --- | --- | --- | --- | --- | --- | --- | --- | --- |
|  |  | |  |  |  |  | |  |
|  |  | | My partner or husband | 1 |  |  | |  |
|  |  | | Birth parents | 2 |  |  | |  |
|  |  | | Parents-in-law/parents of partner | 3 |  |  | |  |
|  |  | | Male members of the community | 4 |  |  | |  |
|  |  | | Female members of the community | 5 |  |  | |  |
|  |  | | Other - INTERVIEWER FIND OUT & WRITE IN | 6 |  |  | |  |
|  |  | | Prefer not to say | 97 |  |  | |  |
|  |  | | Don’t know | 98 |  |  | |  |

ASK ALL

| Q35. | | **How often, if at all, have you helped a neighbour from your community in the past four weeks? By neighbour, this can include your relatives and friends who live nearby.** SINGLE CODE ONLY. | | | | |  | |
| --- | --- | --- | --- | --- | --- | --- | --- | --- |
|  |  | |  |  |  |  | |  |
|  |  | | CODE “1” AND WRITE IN THE NUMBER OF TIMES | 1  ☐☐ |  |  | |  |
|  |  | | I have not helped a neighbour from my community in the last four weeks | 2 |  |  | |  |
|  |  | | Don’t know | 98 |  |  | |  |

| Q36. | | SHOWCARD 16 **Approximately, what proportion of people, if any, in your local community contribute time or money towards community development? This could be, for example, by building a levy, repairing a road, or visiting someone who is ill.**  **Read out the statement from this card that best applies.** SINGLE CODE ONLY | | | | |  | |
| --- | --- | --- | --- | --- | --- | --- | --- | --- |
|  |  | |  |  |  |  | |  |
|  |  | | Everyone | 1 |  |  | |  |
|  |  | | More than half | 2 |  |  | |  |
|  |  | | About half | 3 |  |  | |  |
|  |  | | Less than half | 4 |  |  | |  |
|  |  | | No one | 5 |  |  | |  |
|  |  | | Don’t know | 98 |  |  | |  |

| Q37. | | SHOWCARD 17 **How likely is it that people in this local community would co-operate to solve a problem such as a water supply issue? Read out the statement from this card that best applies.**  SINGLE CODE ONLY | | | | |  | |
| --- | --- | --- | --- | --- | --- | --- | --- | --- |
|  |  | |  |  |  |  | |  |
|  |  | | Very likely | 1 |  |  | |  |
|  |  | | Fairly likely | 2 |  |  | |  |
|  |  | | Not very likely | 3 |  |  | |  |
|  |  | | Not at all likely | 4 |  |  | |  |
|  |  | | Don’t know | 98 |  |  | |  |

| CRIME AND SAFETY |
| --- |

| Q38. | | SHOWCARD 18 **I would now like to ask you some questions about crime and safety.**  READ OUT A-F. SINGLE CODE FOR EACH | | | | | | | |  |
| --- | --- | --- | --- | --- | --- | --- | --- | --- | --- | --- |
|  |  | |  | Very safe | Fairly safe | Neither safe nor unsafe | Fairly unsafe | Very unsafe | Don’t know | |
|  | a) | | How safe do you feel when walking alone in the area near your home during the daytime? | 1 | 2 | 3 | 4 | 5 | 98 | |
|  | b) | | How safe do you feel when walking with a woman in the area near your home during the daytime? | 1 | 2 | 3 | 4 | 5 | 98 | |
|  | c) | | How safe do you feel when walking with a man in the area near your home during the daytime? | 1 | 2 | 3 | 4 | 5 | 98 | |
|  | d) | | How safe do you feel when walking alone in the area near your home after dark? | 1 | 2 | 3 | 4 | 5 | 98 | |
|  | e) | | How safe do you feel when walking with a woman in the area near your home after dark? | 1 | 2 | 3 | 4 | 5 | 98 | |
|  | f) | | Finally, how safe do you feel when walking with a man in the area near your home after dark? | 1 | 2 | 3 | 4 | 5 | 98 | |

| Q39. | | SHOWCARD 19 **Thinking about the next 12 months, how worried are you about being a…**  READ OUT A and B. SINGLE CODE FOR EACH | | | | | | | |  |
| --- | --- | --- | --- | --- | --- | --- | --- | --- | --- | --- |
|  |  | |  | Very worried | Fairly worried | Not very worried | Not at all worried | Don’t know |  | |
|  | a) | | …victim of a violent assault at home? | 1 | 2 | 3 | 4 | 98 |  | |
|  | b) | | …victim of a violent assault outside of your home? | 1 | 2 | 3 | 4 | 98 |  | |

ASK Q40 IF Q39a OR b IS CODED 1 OR 2. IF Q39a OR b ARE NOT CODED 1 OR 2, GO TO Q41.

| Q40. | | SHOWCARD 20 **As a result of these worries, will you reduce or stop doing any of these things? Read out the statement or statements that apply.** | | | | |  | |
| --- | --- | --- | --- | --- | --- | --- | --- | --- |
|  |  | |  |  |  |  | |  |
|  |  | | Attending community groups, clubs or societies | 1 |  |  | |  |
|  |  | | Visiting family | 2 |  |  | |  |
|  |  | | Visiting friends | 3 |  |  | |  |
|  |  | | Attending social events (e.g. cinema, dances, etc) | 4 |  |  | |  |
|  |  | | Attending Religious Services | 5 |  |  | |  |
|  |  | | Walking after dark by yourself | 6 |  |  | |  |
|  |  | | Walking in the day time by yourself | 7 |  |  | |  |
|  |  | | Helping friends or neighbours | 8 |  |  | |  |
|  |  | | Playing with your children | 9 |  |  | |  |
|  |  | | Other - INTERVIEWER FIND OUT & WRITE IN | 10 |  |  | |  |
|  |  | | None of these | 11 |  |  | |  |
|  |  | | Prefer not to say | 97 |  |  | |  |
|  |  | | Don’t know | 98 |  |  | |  |

ASK ALL

| Q41. | | **Differences can sometimes exist between people living in the same community. I will read out some differences between people in local communities. Please tell me if the differences I read out ever lead to problems in your community.**  READ OUT A TO G. SINGLE CODE FOR EACH | | | | | | |  | |
| --- | --- | --- | --- | --- | --- | --- | --- | --- | --- | --- |
|  |  | | **Differences between...** | Yes | No | Don’t know |  |  | |  |
|  | a) | | …people with different social status’? | 1 | 2 | 98 |  |  | |  |
|  | b) | | …men and women? | 1 | 2 | 98 |  |  | |  |
|  | c) | | …older and younger people? | 1 | 2 | 98 |  |  | |  |
|  | d) | | …people with different political affiliations? | 1 | 2 | 98 |  |  | |  |
|  | e) | | …people with different religious beliefs? | 1 | 2 | 98 |  |  | |  |
|  | f) | | …people with different ethnic backgrounds? | 1 | 2 | 98 |  |  | |  |
|  | g) | | Other (INTERVIEWER FIND OUT & WRITE IN) | 1 | 2 | 98 |  |  | |  |

ASK Q42 FOR EACH GROUP CODED ‘1’ AT Q41. IF Q41 A-G IS ALL CODED ‘2’ OR ‘98’, GO TO Q43.

| Q42. | | **Do these differences ever lead to public quarrels, street fights or confrontations…?**  READ OUT THOSE GROUPS CODED 1 AT Q41. SINGLE CODE FOR EACH | | | | | | |  | |
| --- | --- | --- | --- | --- | --- | --- | --- | --- | --- | --- |
|  |  | | **Differences between...** | Yes | No | Don’t know |  |  | |  |
|  | a) | | People with different social status | 1 | 2 | 98 |  |  | |  |
|  | b) | | Men and women | 1 | 2 | 98 |  |  | |  |
|  | c) | | Older and younger people | 1 | 2 | 98 |  |  | |  |
|  | d) | | People with different political affiliations | 1 | 2 | 98 |  |  | |  |
|  | e) | | People with different religious beliefs | 1 | 2 | 98 |  |  | |  |
|  | f) | | People with different ethnic backgrounds | 1 | 2 | 98 |  |  | |  |
|  | g) | | Other - INTERVIEWER FIND OUT & WRITE IN | 1 | 2 | 98 |  |  | |  |

ASK ALL

| Q43. | | **Have you ever witnessed a public quarrel, street fight or confrontation in your local community?**  SINGLE CODE ONLY | | | | |  | |
| --- | --- | --- | --- | --- | --- | --- | --- | --- |
|  |  | |  |  |  |  | |  |
|  |  | | Yes | 1 | ask q44 |  | |  |
|  |  | | No | 2 | go to q45 |  | |  |
|  |  | | Don’t know | 98 |  |  | |  |

ASK Q44 IF YES AT Q43. OTHERS GO TO Q45.

| Q44. | | **How often has this happened in the last 12 months?** | | | | |  | |
| --- | --- | --- | --- | --- | --- | --- | --- | --- |
|  |  | |  |  |  |  | |  |
|  |  | | CODE “1” AND WRITE IN THE NUMBER | 1  ☐☐ |  |  | |  |
|  |  | | Don’t know | 98 |  |  | |  |

ASK ALL

| Q45. | | SHOWCARD 21 **How confident are you that the local authorities such as the police can protect you and members of your household from crime and violence? Read out the response from this card that best applies.**  SINGLE CODE ONLY | | | | |  | |
| --- | --- | --- | --- | --- | --- | --- | --- | --- |
|  |  | |  |  |  |  | |  |
|  |  | | Very confident | 1 |  |  | |  |
|  |  | | Fairly confident | 2 |  |  | |  |
|  |  | | Not very confident | 3 |  |  | |  |
|  |  | | Not at all confident | 4 |  |  | |  |
|  |  | | Don’t know | 98 |  |  | |  |

ASK ALL

| MARITAL RELATIONSHIPS |
| --- |

| Q46a. SHOWCARD 22 **Which one of these best describes your marital status?**  SINGLE CODE ONLY  CODE 3 = PAKISTAN ONLY  CODE 4 = GHANA AND SOUTH SUDAN ONLY | | | | |  |
| --- | --- | --- | --- | --- | --- |
|  |  |  | |  | |
|  | Never married | 1 | GO TO Q57A | | |
|  | Married INCLUDE THE TEXT IN BRACKETS FOR PAKISTAN ONLY (court, church, nikahmah or community) | 2 | ASK Q47 in ghana and pakistan.  ask q46b in south sudan | | |
|  | Married with no official document | 3 |  |  |  |
|  | Unmarried – living with a boyfriend or fiancé | 4 | GO TO q47 | | |
|  | Have a boyfriend or fiancé but not living with him | 5 |  |  |  |
|  | Divorced or separated | 6 | GO TO Q51 | | |
|  | Widowed | 7 | GO TO Q57A | | |
|  | Prefer not to say | 97 |  |  |  |

ASK Q46B IN SOUTH SUDAN IF THE WOMAN IS MARRIED (THIS IS CODES 2 AT Q46A). OTHERS GO TO Q47.

| Q46b. | | **Do you live with your husband?**  SINGLE CODE ONLY | | | | |  | |
| --- | --- | --- | --- | --- | --- | --- | --- | --- |
|  |  | |  |  |  |  | |  |
|  |  | | Yes | 1 |  |  | |  |
|  |  | | No | 2 |  |  | |  |
|  |  | | Prefer not to say | 97 |  |  | |  |
|  |  | | Don’t know | 98 |  |  | |  |

ASK Q46C. IF THE WOMAN DOES NOT LIVE WITH HER HUSBAND (THIS IS CODE 2 AT Q46B). OTHERS GO TO Q47.

| Q46c. | | **Why do you not live with your husband?**  MULTICODE OKAY | | | | |  | |
| --- | --- | --- | --- | --- | --- | --- | --- | --- |
|  |  | |  |  |  |  | |  |
|  |  | | He lives or works elsewhere | 1 |  |  | |  |
|  |  | | Not living with him because of the war | 2 |  |  | |  |
|  |  | | He is missing due to war | 3 |  |  | |  |
|  |  | | HE IS IN PRISON | 4 |  |  | |  |
|  |  | | We have separated | 5 |  |  | |  |
|  |  | | I have moved in with my parents | 6 |  |  | |  |
|  |  | | Other – INTERVIEWER FIND OUT & WRITE IN | 7 |  |  | |  |
|  |  | | Prefer not to say | 97 |  |  | |  |

ASK Q47 TO Q50 IF THE WOMAN IS MARRIED. THAT IS EITHER CODE 2 OR 3 AT Q46A. IF DIVORCED OR SEPARATED (CODE 7) GO TO Q51; IF WIDOWED (CODE 8) OR CODE 97 GO TO Q57A.

| Q47. | | **Does or did your husband or fiancé have any other wives?**  SINGLE CODE ONLY | | | | |  | |
| --- | --- | --- | --- | --- | --- | --- | --- | --- |
|  |  | |  |  |  |  | |  |
|  |  | | Yes | 1 |  |  | |  |
|  |  | | No | 2 |  |  | |  |
|  |  | | Prefer not to say | 97 |  |  | |  |
|  |  | | Don’t know | 98 |  |  | |  |

| Q48. | | **How old were you when you first got married or engaged?**  SINGLE CODE ONLY | | | | |  | |
| --- | --- | --- | --- | --- | --- | --- | --- | --- |
|  |  | |  |  |  |  | |  |
|  |  | | WRITE IN AGE AND CODE “1” | 1  ☐☐ |  |  | |  |
|  |  | | Don’t know | 98 |  |  | |  |

| Q49. | | SHOWCARD 23 **Who decided that you should get married or engaged? Read out the statement from this card that best applies.**  SINGLE CODE ONLY | | | | |  | |
| --- | --- | --- | --- | --- | --- | --- | --- | --- |
|  |  | |  |  |  |  | |  |
|  |  | | I chose to get married | 1 | GO TO Q53 |  | |  |
|  |  | | My partner asked me to marry him | 2 |  |  | |  |
|  |  | | My parents decided for me | 3 |  |  | |  |
|  |  | | My partner’s parents decided for us | 4 | ASK Q50 |  | |  |
|  |  | | Other - INTERVIEWER FIND OUT & WRITE IN | 5 |  |  | |  |
|  |  | | Prefer not to say | 97 | GO TO Q53 |  | |  |

ASK Q50 IF CODES 3, 4 OR 5 AT Q49. OTHERS GO TO Q53.

| Q50. | | **Was your consent sought for the marriage?**  IF YES ASK **And did you agree to the marriage?**  SINGLE CODE ONLY | | | | |
| --- | --- | --- | --- | --- | --- | --- |
|  |  | |  |  |  |  |
|  |  | | Yes and I agreed | 1 |  |  |
|  |  | | Yes but I did not agree | 2 |  |  |
|  |  | | No, my consent was not sought | 3 |  |  |
|  |  | | Prefer not to say | 97 |  |  |
|  |  | | Don’t know | 98 |  |  |

ASK Q51 AND Q52 IF DIVORCED OR SEPARATED (CODE 6 AT Q46a). OTHERS GO TO Q53.

| Q51. | | SHOWCARD 24 **Who decided that you should divorce or separate? Read out the statement or statements from this card that best applies.**  MULTICODE OKAY | | | | |  | |
| --- | --- | --- | --- | --- | --- | --- | --- | --- |
|  |  | |  |  |  |  | |  |
|  |  | | I did | 1 |  |  | |  |
|  |  | | My husband did | 2 |  |  | |  |
|  |  | | Both my husband and I decided between us | 3 |  |  | |  |
|  |  | | My parents | 4 |  |  | |  |
|  |  | | My husband’s parents | 5 |  |  | |  |
|  |  | | Other - INTERVIEWER FIND OUT & WRITE IN | 6 |  |  | |  |
|  |  | | Prefer not to say | 97 |  |  | |  |
|  |  | | Don’t know | 98 |  |  | |  |

| Q52. | | SHOWCARD 25 **Can you tell me why you divorced or separated from your husband? Read out the statement or statements from this card that apply.**  MULTICODE OKAY | | | | |  | |
| --- | --- | --- | --- | --- | --- | --- | --- | --- |
|  |  | |  |  |  |  | |  |
|  |  | | Financial reasons | 1 |  |  | |  |
|  |  | | Because of domestic abuse or violence | 2 |  |  | |  |
|  |  | | Because I could not have children | 3 |  |  | |  |
|  |  | | Because we stopped loving each other | 4 |  |  | |  |
|  |  | | Other reason - INTERVIEWER FIND OUT & WRITE IN | 5 |  |  | |  |
|  |  | | Prefer not to say | 97 |  |  | |  |
|  |  | | Don’t know | 98 |  |  | |  |

ASK Q53 IF MARRIED OR HAVE A CURRENT PARTNER. THIS WILL BE CODES 2, 3 4, OR 5 AT Q46a. OTHERS GO TO Q57a.

| Q53. | | **How long have you been married or with your current partner?**  SINGLE CODE ONLY | | | | |  | |
| --- | --- | --- | --- | --- | --- | --- | --- | --- |
|  |  | |  |  |  |  | |  |
|  |  | | WRITE IN NUMBER OF YEARS AND MONTHS | 1  ☐☐/☐☐  YEARS MONTHS |  |  | |  |
|  |  | | Don’t know | 98 |  |  | |  |

ASK Q54 IF MARRIED. THIS IS CODES 2, OR 3 AT Q46a. OTHERS GO TO Q55.

| Q54. | | **Do you live with your husband’s birth parents or his birth relatives?**  SINGLE CODE ONLY | | | | |  | |
| --- | --- | --- | --- | --- | --- | --- | --- | --- |
|  |  | |  |  |  |  | |  |
|  |  | | Yes | 1 |  |  | |  |
|  |  | | No | 2 |  |  | |  |
|  |  | | Prefer not to say | 97 |  |  | |  |

ASK Q55 IF MARRIED OR HAVE A CURRENT PARTNER. THIS WILL BE CODES 2, 3 4, OR 5 AT Q46a. OTHERS GO TO Q57A IN THE NEXT SECTION.

| Q55. | | **How old is your husband or partner?**  SINGLE CODE ONLY | | | | |  | |
| --- | --- | --- | --- | --- | --- | --- | --- | --- |
|  |  | |  |  |  |  | |  |
|  |  | | WRITE IN NUMBER OF YEARS AND CODE “1” | 1  ☐☐ |  |  | |  |
|  |  | | Don’t know | 98 |  |  | |  |

| Q56. | | SHOWCARD 26 **What is the highest level of education your husband or partner has obtained? Read out the category from this card that best applies.**  SINGLE CODE ONLY | | | | |
| --- | --- | --- | --- | --- | --- | --- |
|  |  | |  |  |  |  |
|  |  | | Pre-school | 1 |  |  |
|  |  | | Primary | 2 |  |  |
|  |  | | Middle/Junior Secondary School/Junior High School | 3 |  |  |
|  |  | | Secondary/Senior Secondary School/Senior High School | 4 |  |  |
|  |  | | Technical/Vocational | 5 |  |  |
|  |  | | Higher/University | 6 |  |  |
|  |  | | Don’t know | 98 |  |  |

ASK ALL

| PAID AND UNPAID WORK |
| --- |

**As you might know, some women and girls take up jobs for which they are paid in cash or kind. Others sell things, have a small business or work on the family farm or in the family business. I would now like to ask you some questions about your work. This includes both paid work and unpaid work that you might do. If you have a husband or partner, I will also ask you some questions about the paid or unpaid work (both paid and unpaid) they do.**

| Q57a.  Q57b. | | **Are you currently involved in any work?**  ASK Q57B IF THE WOMAN OR GIRL HAS A HUSBAND OR PARTNER. THIS WILL BE CODES 2, 3 4, OR 5 AT Q46a. OTHERS GO TO Q58a.  **And is your husband or partner also involved in any work?**  SINGLE CODE ONLY | | | | | | |  | |
| --- | --- | --- | --- | --- | --- | --- | --- | --- | --- | --- |
|  |  | |  | Q57a  Woman or girl | Q57b  Partner |  |  |  | |  |
|  |  | | Yes | 1 | 1 |  |  |  | |  |
|  |  | | No | 2 | 2 |  |  |  | |  |
|  |  | | Don’t know | 3 | 3 |  |  |  | |  |

| Q58a.  Q58b. | | ASK Q58A IF THE WOMAN OR GIRL WORKS (CODE 1 AT Q57A). SHOWCARD 26 **Which one of these describes the work that you do?**  **Read out the category that applies.**  PROBE ALL ACTIVITIES.  SINGLE CODE ONLY  ASK Q58B IF THE WOMAN OR GIRL HAS A HUSBAND OR PARTNER WHO WORKS (CODE 1 AT Q57B).    SHOWCARD 27 AGAIN **And which one of these describes the work that your husband or partner does?** PROBE ALL ACTIVITIES. SINGLE CODE ONLY | | | | | | |  | |
| --- | --- | --- | --- | --- | --- | --- | --- | --- | --- | --- |
|  |  | |  | Q58a  Woman or girl | Q58b  Husband or Partner |  |  |  | |  |
|  |  | | Regular salaried | 1 | 1 |  |  |  | |  |
|  |  | | Contract | 2 | 2 |  |  |  | |  |
|  |  | | Self-employed - non-agriculture | 3 | 3 |  |  |  | |  |
|  |  | | Unpaid family worker – non-agricultural | 4 | 4 |  |  |  | |  |
|  |  | | Self-employed – agriculture | 5 | 5 |  |  |  | |  |
|  |  | | Unpaid family worker – agriculture | 6 | 6 |  |  |  | |  |
|  |  | | Q58a: Other reason - INTERVIEWER FIND OUT & WRITE IN | 7 |  |  |  |  | |  |
|  |  | | Q58b: Other reason - INTERVIEWER FIND OUT & WRITE IN |  | 7 |  |  |  | |  |
|  |  | | Prefer not to say | 97 | 97 |  |  |  | |  |
|  |  | | Don’t know | 98 | 98 |  |  |  | |  |

| Q59a.  Q59b. | | ASK Q59A IF THE WOMAN OR GIRL WORKS (CODE 1 AT Q57A).  SHOWCARD 28 **Now** **look at this card and tell me** **which of these best describes the sector in which you work?**  ASK Q59B IF THE WOMAN OR GIRL’S HUSBAND OR PARTNER WORKS(CODE 1 AT Q57B).  SHOWCARD 28 AGAIN **And which one of these best describes the sector in which your husband or partner works?** SINGLE CODE ONLY | | | | | | |  | |
| --- | --- | --- | --- | --- | --- | --- | --- | --- | --- | --- |
|  |  | |  | Q59a  Woman or girl | Q59b  Husband or Partner |  |  |  | |  |
|  | A | | Agriculture | 1 | 1 |  |  |  | |  |
|  | B | | Fishing and forestry | 2 | 2 |  |  |  | |  |
|  | C | | Mining and quarrying | 3 | 3 |  |  |  | |  |
|  | D | | Manufacturing | 4 | 4 |  |  |  | |  |
|  | E | | Construction | 5 | 5 |  |  |  | |  |
|  | F | | Wholesale and retail | 6 | 6 |  |  |  | |  |
|  | G | | Transportation and storage | 7 | 7 |  |  |  | |  |
|  | H | | Accommodation and food | 8 | 8 |  |  |  | |  |
|  | I | | Education | 9 | 9 |  |  |  | |  |
|  | J | | Human health and social work | 10 | 10 |  |  |  | |  |
|  | K | | Q59a. Other industry - INTERVIEWER FIND OUT & WRITE IN | 11 |  |  |  |  | |  |
|  |  | | Q59b. Other industry - INTERVIEWER FIND OUT & WRITE IN |  | 11 |  |  |  | |  |
|  |  | | Prefer not to say | 97 | 97 |  |  |  | |  |
|  |  | | Don’t know | 98 | 98 |  |  |  | |  |

ASK Q60 IF THE WOMAN OR GIRL WORKS (CODE 1 AT Q57A).

**I will now ask you more questions about the work you do.**

| Q60. | | **Please describe the work that you do. This can be either paid or unpaid work, for example work in rice cultivation, factory worker, typist, shop assistant and so on.**  PROBE FULLY AND WRITE IN BELOW | | | | |  | |
| --- | --- | --- | --- | --- | --- | --- | --- | --- |
|  |  | |  |  |  |  | |  |
|  |  | |  | |  |  | |  |
|  |  | |  |  |  |  | |  |
|  |  | |  |  |  |  | |  |

ASK Q61 IF THE WOMAN OR GIRL’S HUSBAND OR PARTNER WORKS (CODE 1 AT Q57B). IF THE WOMEN DOES NOT HAVE A HUSBAND OR PARTNER GO TO Q62A.

| Q61. | | **Please describe the work that your husband or partner does. Again, this can be either paid or unpaid work, for example work in rice cultivation, factory worker, typist, shop assistant and so on.**  PROBE FULLY AND WRITE IN BELOW | | | | |  | |
| --- | --- | --- | --- | --- | --- | --- | --- | --- |
|  |  | |  |  |  |  | |  |
|  |  | |  | |  |  | |  |
|  |  | |  |  |  |  | |  |
|  |  | |  |  |  |  | |  |

| Q62a.  Q62b. | ASK Q62A IF THE WOMAN OR GIRL WORKS (CODE 1 AT Q57A).  SHOWCARD 29 **What is your current job or occupation?**  SINGLE CODE ONLY  ASK Q62B IF THE WOMAN OR GIRL’S HUSBAND OR PARTNER WORKS (CODE 1 AT Q57B)  SHOWCARD 29 AGAIN **And what is the current job or occupation of your husband or partner?** SINGLE CODE ONLY |
| --- | --- |

| INTERVIEWER: SHOW SHOWCARD 29 WITH LIST OF OCCUPATIONS. IF RESPONDENT UNSURE PROBE FOR TYPE OF JOB AND ASK WHICH TYPES OF JOBS COME CLOSEST | | |
| --- | --- | --- |
|  | **Q62a** | **Q62b** |
| **Elementary occupations**  Such as domestic, hotel and office cleaners, building construction labourers, garbage and recycling collectors, street vendors (excluding food), domestic helpers, window cleaners, shelf fillers, hand packers, unskilled factory workers, kitchen/catering assistants, food preparation assistants, postal workers, road sweepers, refuse sorters, traffic wardens. Agricultural, forestry and fishery labourers, fruit and vegetable pickers. Labourers, packers, goods handling and storage staff. Labourers in mining, manufacturing and transport. Odd job persons. | 1 | 1 |
| **Plant and Machine Operator and Assembler**  Such as transport and mobile machine drivers, plant and machine operators, routine operatives (sorters, assemblers), HGV, van, fork lift, train, bus and taxi drivers | 2 | 2 |
| **Building, Crafts or a Related Trade Person**  Such as electricians, motor mechanics, machine repairers, metal workers, blacksmiths, welders, TV engineers, plumbers, builders, bricklayers, carpenters, painters, printers, butchers, bakers, furniture makers, foremen, tailors, seamstresses. Producers of handicrafts. | 3 | 3 |
| **A Skilled Agricultural, Forestry and Fishery Worker**  Such as dairy producers, landscape gardeners and horticultural workers, subsistence farmers and fishermen/women. | 4 | 4 |
| **A Sales, Customer or Personal Service Worker**  Such as shopkeepers, street and market stall sales people, street food sales people, sales assistants and retail cashiers, telesales persons, call centre agents, waiters/waitresses and bartenders, customer care occupations, travel attendants and travel guides.  Personal care workers such as those providing care to children, elderly and disabled people; ambulance workers, healthcare assistants, teaching assistants.  Personal service workers such as hairdressers, beauticians, cooks, driving instructors, undertakers and housekeepers.  Protective service workers such as security guards, and junior police officers, fire-fighters, prison officers, building caretakers. | 5 | 5 |
| **Clerical Support**  Such as secretaries, receptionists, telephonists, book-keepers, travel agents, accounting clerks, credit controllers/wage clerks, assistants/clerks, communication operators, market research interviewers, pension and insurance clerks, office assistants, database assistants, data entry clerks, postmen/women. | 6 | 6 |
| **A Technician or Associate Professional**  Such as science, engineering and IT technicians, accounting technicians, manufacturing/ construction supervisors, draughtspersons, insurance brokers/agents, finance and investment analysts and advisers, buyers, estate agents, specialised secretaries (legal or medical), air traffic controllers, pilots, graphic designers, , chefs, junior nurses, dental assistants, opticians, therapists,  community workers, careers advisors, health and safety officers, housing officers, police inspectors and detectives, photographers, interior designers, sports players. | 7 | 7 |
| **A Professional**  Such as professional engineers, software and IT professionals, accountants, chemists, scientific researchers, solicitors and lawyers, economists, architects, actuaries, doctors, senior nurses, midwives, psychologists, teachers, social workers, librarians, actors, artists, authors, writers/journalists, musicians. | 8 | 8 |
| **A Manager**  Such as chief executives, senior government officials, legislators, managing directors, senior business managers, senior production managers, senior service managers, sales and marketing managers, human resource managers, bank managers, hotel managers, restaurant managers, factory owners. | 9 | 9 |
| **Employed in a military capacity by the Armed Forces** | 10 | 10 |
| Other (please specify): | 11 | 11 |
| Prefer not to say | 97 | 97 |
| Don’t know | 98 | 98 |
| Doesn’t understand the question | 99 | 99 |

| Q63a.  Q63b. | | ASK Q63A IF THE WOMAN OR GIRL WORKS (CODE 1 AT Q57A).  SHOWCARD 30 **Look at this card and tell me** **which one of these best describes how often you work during the year. Read out the category that best applies.**  SINGLE CODE ONLY  ASK Q63B IF THE WOMAN OR GIRL’S HUSBAND OR PARTNER WORKS. (CODE 1 AT Q57B).  SHOWCARD 30 AGAIN **And still looking at the card. Which one best describes how often your husband or partner works during the year. Read out the category that best applies.**  SINGLE CODE ONLY | | | | | | |  | |
| --- | --- | --- | --- | --- | --- | --- | --- | --- | --- | --- |
|  |  | |  | Q63a  Woman | Q63b  Husband or Partner |  |  |  | |  |
|  |  | | Work throughout the year - full-time | 1 | 1 |  |  |  | |  |
|  |  | | Work throughout the year - part-time | 2 | 2 |  |  |  | |  |
|  |  | | Seasonal worker | 3 | 3 |  |  |  | |  |
|  |  | | Work whenever I can find a job | 4 | 4 |  |  |  | |  |
|  |  | | Q63a: Other reason - INTERVIEWER FIND OUT & WRITE IN | 5 |  |  |  |  | |  |
|  |  | | Q63b: Other reason - INTERVIEWER FIND OUT & WRITE IN |  | 5 |  |  |  | |  |
|  |  | | Prefer not to say | 97 | 97 |  |  |  | |  |
|  |  | | Don’t know | 98 | 98 |  |  |  | |  |

| Q64a.  Q64b. | | ASK Q64A IF THE WOMAN OR GIRL WORKS (CODE 1 AT Q57A).  **In the last 12 months, how many months did you work on your main job? By main job, I mean the job you spend most of your time working in. It does not necessarily have to be on a continuous basis throughout the entire 12 months.**  INTERVIEWER NOTE FOR THESE TWO QUESTIONS: IF THE WOMAN OR GIRL DOES NOT KNOW THE NUMBER OF MONTHS IN THE PAST 12 MONTHS, ASK HER ABOUT HOW MANY WEEKS WORKED WITHIN THE PAST MONTH AND WRITE IN AT PART B.  AND IF SHE DOESN’T KNOW HOW MANY MONTHS ASK HER HOW MANY DAYS IN THE PAST WEEK AT PART C. IF SHE DOESN’T KNOW THE NUMBER OF DAYS, ASK HER HOW MANY HOURS WORKED ON THE LAST DAY WORKED AT PART D.  ASK Q64B IF THE WOMAN OR GIRL’S HUSBAND OR PARTNER WORKS. (CODE 1 AT Q57B)  **And** **in the last 12 months, how many months did your husband or partner work on his main job? By main job, I mean the job he spends most of his time working in. It does not necessarily have to be on a continuous basis throughout the entire 12 months.** | | | | | | |  | |
| --- | --- | --- | --- | --- | --- | --- | --- | --- | --- | --- |
|  |  | |  | Q64a  Woman | Q64b  Husband or Partner |  |  |  | |  |
|  | a) | | How many months in the last 12 months CODE ‘1’ AND WRITE IN | 1  ☐☐ | 1  ☐☐ |  |  |  | |  |
|  | b) | | During these months approximately how many weeks in a month?  CODE ‘2’ AND WRITE IN | 2  ☐☐ | 2  ☐☐ |  |  |  | |  |
|  | c) | | In these weeks, approximately how many days in the week?  CODE ‘3’ AND WRITE IN | 3  ☐☐ | 3  ☐☐ |  |  |  | |  |
|  | d) | | In these days, approximately how many hours in the day?  CODE ‘4’ AND WRITE IN | 4  ☐☐ | 4  ☐☐ |  |  |  | |  |
|  |  | | Prefer not to say | 97 | 97 |  |  |  | |  |

| Q65a.  Q65b. | | ASK Q65A IF THE WOMAN OR GIRL WORKS (CODE 1 AT Q57A).  **How much was the last payment of wage or salary you received for the work you did on your main job?**  SINGLE CODE ONLY  ASK Q65B IF THE WOMAN OR GIRL’S HUSBAND OR PARTNER WORKS (CODE 1 AT Q57B) **And how much was the last payment your husband or partner received for the work they did on their main job?**  SINGLE CODE ONLY | | | |  | |
| --- | --- | --- | --- | --- | --- | --- | --- |
|  |  | | CODE ‘1’ AND WRITE IN AMOUNT IN LOCAL CURRENCY | Q65a  Woman  1  ☐☐☐☐☐ | Q65b  Husband or Partner  1  ☐☐☐☐☐ | |  |
|  |  | | No earnings | 2 | 2 | |  |
|  |  | | Prefer not to say | 97 | 97 | |  |
|  |  | | Don’t know | 98 | 98 | |  |

| Q66a.  Q66b. | | ASK Q66A IF THE WOMAN OR GIRL WORKS AND EARNS A WAGE OR SALARY. THIS IS CODE 1 AT Q65A.  **And thinking about the last payment you received for the work you did on your main job. To what time period do these earnings correspond to?**  SINGLE CODE ONLY  ASK Q66B IF THE WOMAN OR GIRL’S HUSBAND OR PARTNER WORKS AND EARNS A WAGE OR SALARY. THIS IS CODE 1 AT Q65B.  **And thinking about the last payment of wage or salary your husband or partner received for the work they did on their main job, to what time period do these earnings correspond to?**  SINGLE CODE ONLY | | | | |  | | | |
| --- | --- | --- | --- | --- | --- | --- | --- | --- | --- | --- |
|  |  | |  | Q66a  Woman | Q66b  Husband or Partner |  | |  |  |  |
|  |  | | Per hour  CODE “1” AND WRITE IN AMOUNT | 1  ☐☐ | 1  ☐☐ |  | |  |  |  |
|  |  | | Per day  CODE “2” AND WRITE IN AMOUNT | 2  ☐☐ | 2  ☐☐ |  | |  |  |  |
|  |  | | Per week  CODE “3” AND WRITE IN AMOUNT | 3  ☐☐☐ | 3  ☐☐☐ |  | |  |  |  |
|  |  | | Per every two weeks  CODE “4” AND WRITE IN AMOUNT | 4  ☐☐☐ | 4  ☐☐☐ |  | |  |  |  |
|  |  | | Per month  CODE “5” AND WRITE IN AMOUNT | 5  ☐☐☐☐ | 5  ☐☐☐☐ |  | |  |  |  |
|  |  | | Per year  CODE “6” AND WRITE IN AMOUNT | 6  ☐☐☐☐☐ | 6  ☐☐☐☐☐ |  | |  |  |  |
|  |  | | Prefer not to say | 97 | 97 |  | |  |  |  |
|  |  | | Don’t know | 98 | 98 |  | |  |  |  |

| Q67. | | ASK Q67 IF THE WOMAN OR GIRL WORKS AND EARNS A WAGE OR SALARY.  SHOWCARD 31 **Thinking now about the money you earned in the last 12 months. Which, if any, of these things did you do with it? Read out the statement or statements that apply.** MULTICODE OKAY. | | | | |  | |
| --- | --- | --- | --- | --- | --- | --- | --- | --- |
|  |  | |  |  |  |  | |  |
|  |  | | I spent it on myself | 1 |  |  | |  |
|  |  | | I chose to give part of it to my husband or partner | 2 |  |  | |  |
|  |  | | I had to give part of it to my husband or partner against my will | 3 |  |  | |  |
|  |  | | I chose to give it all to my husband or partner | 4 |  |  | |  |
|  |  | | I had to give it all to my husband or partner against my will | 5 |  |  | |  |
|  |  | | I chose to give all or part to my parents, in-laws or my adult children | 6 |  |  | |  |
|  |  | | Prefer not to say | 97 |  |  | |  |
|  |  | | Don’t know | 98 |  |  | |  |

| Q68. | | ASK Q68 IF THE WOMAN OR GIRL’S HUSBAND OR PARTNER WORKS AND EARNS A WAGE OR SALARY  **Did your husband or partner give his earnings to you to use for household expenses?** IF YES ASK **Is that all of his earnings or a part of his earnings?**  SINGLE CODE ONLY | | | | |  | |
| --- | --- | --- | --- | --- | --- | --- | --- | --- |
|  |  | |  |  |  |  | |  |
|  |  | | Yes – all of his earnings | 1 |  |  | |  |
|  |  | | Yes – part of his earnings | 2 |  |  | |  |
|  |  | | No – none of his earnings | 3 |  |  | |  |
|  |  | | Prefer not to say | 97 |  |  | |  |
|  |  | | Don’t know | 98 |  |  | |  |

ASK ALL

| Q69a.  Q69b. | | SHOWCARD 32 **Which, if any, of these things do you have?**  MULTICODE OKAY  ASK Q69B IF THE WOMAN OR GIRL HAS A HUSBAND OR PARTNER (CODES 2, 3 4 OR 5 AT Q46). OTHERS GO TO Q70.  SHOWCARD 32 AGAIN **And which, if any, of these things does your husband or partner have?**  MULTICODE OKAY | | | | | | |  | |
| --- | --- | --- | --- | --- | --- | --- | --- | --- | --- | --- |
|  |  | |  | Q69a  Woman | Q69b  Husband or Partner |  |  |  | |  |
|  |  | | Health insurance | 1 | 1 |  |  |  | |  |
|  |  | | Social Security | 2 | 2 |  |  |  | |  |
|  |  | | Unemployment insurance | 3 | 3 |  |  |  | |  |
|  |  | | A pension that you pay into | 4 | 4 |  |  |  | |  |
|  |  | | Prefer not to say | 97 | 97 |  |  |  | |  |
|  |  | | Don’t know | 98 | 98 |  |  |  | |  |

ASK Q70/Q71/Q72. IF THE WOMAN OR GIRL WORKS (CODE 1 AT Q57A). OTHERS GO TO Q73.

**I would now like to ask you a few more questions about your work. For each question, think about the days you normally work.**

| Q70. | | SHOWCARD 33 **In the last four weeks, how many days of work did you miss because…?** READ OUT. SINGLE CODE FOR EACH | | | | | | |  | |  |
| --- | --- | --- | --- | --- | --- | --- | --- | --- | --- | --- | --- |
|  |  | |  | One day | Two days | Between 3 and 5 days | Between 6 and 10 days | 10 or more days | | No days | Don’t know |
|  | A | | …you were unwell at home | 1 | 2 | 3 | 4 | 5 | | 6 | 8 |
|  | B | | …you had to go to a hospital or a health clinic because you were unwell | 1 | 2 | 3 | 4 | 5 | | 6 | 8 |
|  | C | | …you had to look after a child or other family member because they were unwell | 1 | 2 | 3 | 4 | 5 | | 6 | 8 |
|  | D | | …you had to attend to legal, financial or personal matters | 1 | 2 | 3 | 4 | 5 | | 6 | 8 |
|  | E | | …you did not have enough money for transport to and/or from work | 1 | 2 | 3 | 4 | 5 | | 6 | 8 |

| Q71. | | SHOWCARD 33 AGAIN **And in the last four weeks, how many days were you late for work by at least 1 hour because…?** READ OUT…  SINGLE CODE FOR EACH | | | | | | |  | |  |
| --- | --- | --- | --- | --- | --- | --- | --- | --- | --- | --- | --- |
|  |  | |  | One day | Two days | Between 3 and 5 days | Between 6 and 10 days | 10 or more days | | No days | Don’t know |
|  | A | | …you were unwell at home | 1 | 2 | 3 | 4 | 5 | | 6 | 8 |
|  | B | | …you had to go to a hospital or a health clinic because you were unwell | 1 | 2 | 3 | 4 | 5 | | 6 | 8 |
|  | C | | …you had to look after a child or other family member because they were unwell | 1 | 2 | 3 | 4 | 5 | | 6 | 8 |
|  | D | | …you had to attend to legal, financial or personal matters | 1 | 2 | 3 | 4 | 5 | | 6 | 8 |

| Q72. | | **And for how many days in the last four weeks…?** READ OUT.  SINGLE CODE FOR EACH | | | | | | | |  | |
| --- | --- | --- | --- | --- | --- | --- | --- | --- | --- | --- | --- |
|  | |  | One day | Two days | Between 3 and 5 days | Between 6 and 10 days | 10 or more days | No days | | Don’t know | |
| A | | …did you have difficulties concentrating on your work | 1 | 2 | 3 | 4 | 5 | 6 | | 98 | |
| B | | …did you work much more slowly than you normally would | 1 | 2 | 3 | 4 | 5 | 6 | | 98 | |
| C | | …were you exhausted at work | 1 | 2 | 3 | 4 | 5 | 6 | | 98 | |
| D | | …did you have to stop work because you were worried about something | 1 | 2 | 3 | 4 | 5 | 6 | | 98 | |
| E | | …did you have to stop work because you had an accident at work | 1 | 2 | 3 | 4 | 5 | 6 | | 98 | |

ASK ALL

**In all households, there are additional tasks that must be undertaken. The next questions are about household tasks.**

| Q73a.  Q73b. | | **On average how many hours have you spent on each activity per day in the last seven days?** INTERVIEWER: READ OUT EACH ACTIVITY AND RECORD TIME IN HOURS. IF ACTIVITY NOT UNDERTAKEN PUT ZERO.  ASK Q73B IF THE WOMAN OR GIRL HAS A HUSBAND OR PARTNER (CODES 2, 3, 4 OR 5 AT Q46). IF ACTIVITY NOT UNDERTAKEN PUT ZERO  OTHERS GO TO Q74 IN THE NEXT SECTION.  **And on average how many hours did your husband or partner spend on each activity per day in the last seven days?** INTERVIEWER: READ OUT EACH ACTIVITYAND RECORD TIME IN HOURS. | | | | | | |  | |
| --- | --- | --- | --- | --- | --- | --- | --- | --- | --- | --- |
|  |  | |  | Q73a  Woman or girl | Q73b  Husband or Partner |  |  |  | |  |
|  |  | | Fetching water | 1  ☐☐ | 1  ☐☐ |  |  |  | |  |
|  |  | | Fetching wood | 2  ☐☐ | 2  ☐☐ |  |  |  | |  |
|  |  | | Caring for children | 3  ☐☐ | 3  ☐☐ |  |  |  | |  |
|  |  | | Doing ironing | 4  ☐☐ | 4  ☐☐ |  |  |  | |  |
|  |  | | Washing clothes | 5  ☐☐ | 5  ☐☐ |  |  |  | |  |
|  |  | | Sweeping | 6  ☐☐ | 6  ☐☐ |  |  |  | |  |
|  |  | | Washing dishes | 7  ☐☐ | 7  ☐☐ |  |  |  | |  |
|  |  | | Washing vehicles | 8  ☐☐ | 8  ☐☐ |  |  |  | |  |
|  |  | | Disposing of garbage | 9  ☐☐ | 9  ☐☐ |  |  |  | |  |
|  |  | | Cooking | 10  ☐☐ | 10  ☐☐ |  |  |  | |  |
|  |  | | Shopping for household needs | 11  ☐☐ | 11  ☐☐ |  |  |  | |  |
|  |  | | Running errands | 12  ☐☐ | 12  ☐☐ |  |  |  | |  |
|  |  | | Taking care of livestock and poultry | 13  ☐☐ | 13  ☐☐ |  |  |  | |  |
|  |  | | Making clothes for family members | 14  ☐☐ | 14  ☐☐ |  |  |  | |  |
|  |  | | Prefer not to say | 97 | 97 |  |  |  | |  |
|  |  | | Don’t know | 98 | 98 |  |  |  | |  |

ASK ALL

| GENERAL AND REPRODUCTIVE HEALTH |
| --- |

**I would now like to ask some questions about your health and your usage of healthcare services.**

| Q74. | | SHOWCARD 34 **How would you describe your health?**  SINGLE CODE ONLY | | | | |  | |
| --- | --- | --- | --- | --- | --- | --- | --- | --- |
|  |  | |  |  |  |  | |  |
|  |  | | Excellent | 1 |  |  | |  |
|  |  | | Good | 2 |  |  | |  |
|  |  | | Fair | 3 |  |  | |  |
|  |  | | Poor | 4 |  |  | |  |
|  |  | | Very poor | 5 |  |  | |  |
|  |  | | Prefer not to say | 97 |  |  | |  |
|  |  | | Don’t know | 98 |  |  | |  |

| Q75. | | SHOWCARD 35 **Which, if any, of these health issues do you have?**  MULTICODE OKAY | | | | |  | |
| --- | --- | --- | --- | --- | --- | --- | --- | --- |
|  |  | |  |  |  |  | |  |
|  |  | | Disability (physical) | 1 |  |  | |  |
|  |  | | Medical condition | 2 |  |  | |  |
|  |  | | Other health conditions - INTERVIEWER FIND OUT & WRITE IN) | 3 |  |  | |  |
|  |  | | None of these | 6 |  |  | |  |
|  |  | | Prefer not to say | 97 |  |  | |  |

**The next questions ask about difficulties you may have doing certain activities because of a health problem.**

| Q76 | | **Do you have difficulty seeing, even if wearing glasses?**  IF YES ASK **Is that with some difficulty or a lot of difficulty?** SINGLE CODE ONLY | | | | |  | |
| --- | --- | --- | --- | --- | --- | --- | --- | --- |
|  |  | |  |  |  |  | |  |
|  |  | | No – no difficulty | 1 |  |  | |  |
|  |  | | Yes – some difficulty | 2 |  |  | |  |
|  |  | | Yes – a lot of difficulty | 3 |  |  | |  |
|  |  | | Yes – respondent is blind | 4 |  |  | |  |

| Q77. | | **Do you have difficulty hearing?**  IF YES ASK **Is that with some difficulty or a lot of difficulty?** SINGLE CODE ONLY | | | | |  | |
| --- | --- | --- | --- | --- | --- | --- | --- | --- |
|  |  | |  |  |  |  | |  |
|  |  | | No – no difficulty | 1 |  |  | |  |
|  |  | | Yes – some difficulty | 2 |  |  | |  |
|  |  | | Yes – a lot of difficulty | 3 |  |  | |  |
|  |  | | Yes – respondent is deaf ( DO NOT READ OUT) | 4 |  |  | |  |

| Q78. | | **Do you have difficulty walking or climbing stairs?**  IF YES ASK **Is that with some difficulty, a lot of difficulty or that you cannot do at all?** SINGLE CODE ONLY | | | | |  | |
| --- | --- | --- | --- | --- | --- | --- | --- | --- |
|  |  | |  |  |  |  | |  |
|  |  | | No – no difficulty | 1 |  |  | |  |
|  |  | | Yes – some difficulty | 2 |  |  | |  |
|  |  | | Yes – a lot of difficulty | 3 |  |  | |  |
|  |  | | Yes – cannot do at all | 4 |  |  | |  |

| Q79. | | **Do you have difficulty remembering or concentrating?**  IF YES ASK **Is that with some difficulty or a lot of difficulty?** SINGLE CODE ONLY | | | | |  | |
| --- | --- | --- | --- | --- | --- | --- | --- | --- |
|  |  | |  |  |  |  | |  |
|  |  | | No – no difficulty | 1 |  |  | |  |
|  |  | | Yes – some difficulty | 2 |  |  | |  |
|  |  | | Yes – a lot of difficulty | 3 |  |  | |  |

| Q80. | | **Do you have difficulty speaking?**  IF YES ASK **Is that with some difficulty or a lot of difficulty?**  SINGLE CODE ONLY | | | | |  | |
| --- | --- | --- | --- | --- | --- | --- | --- | --- |
|  |  | |  |  |  |  | |  |
|  |  | | No – no difficulty | 1 |  |  | |  |
|  |  | | Yes – some difficulty | 2 |  |  | |  |
|  |  | | Yes – a lot of difficulty | 3 |  |  | |  |
|  |  | | Yes – respondent cannot speak at all (DO NOT READ OUT) | 4 |  |  | |  |

| Q81. | | **Have you had any health problems in the last four weeks?**  SINGLE CODE ONLY | | | | |  | |
| --- | --- | --- | --- | --- | --- | --- | --- | --- |
|  |  | |  |  |  |  | |  |
|  |  | | Yes | 1 | ask q82 |  | |  |
|  |  | | No | 2 | go to q83 |  | |  |
|  |  | | Don’t know | 3 |  |  | |  |
|  |  | | Prefer not to say | 97 |  |  | |  |

ASK Q82 IF YES AT Q81. OTHERS GO TO Q83

| Q82 | | SHOWCARD 36 **Thinking about the health problems you have experienced in the last four weeks. To what extent, if at all, have these health problems caused issues when carrying out your usual day-to-day activities?**  SINGLE CODE ONLY | | | | |  | |
| --- | --- | --- | --- | --- | --- | --- | --- | --- |
|  |  | |  |  |  |  | |  |
|  |  | | A great deal | 1 |  |  | |  |
|  |  | | A fair amount | 2 |  |  | |  |
|  |  | | Not very much | 3 |  |  | |  |
|  |  | | Not at all | 4 |  |  | |  |
|  |  | | Prefer not to say | 97 |  |  | |  |
|  |  | | Don’t know | 98 |  |  | |  |

ASK ALL

| Q83. | | SHOWCARD 37 **In the last four weeks have you been in any pain or discomfort?**  SINGLE CODE ONLY | | | | |  | |
| --- | --- | --- | --- | --- | --- | --- | --- | --- |
|  |  | |  |  |  |  | |  |
|  |  | | No pain or discomfort | 1 |  |  | |  |
|  |  | | Slight pain or discomfort | 2 |  |  | |  |
|  |  | | Severe pain or discomfort | 3 |  |  | |  |
|  |  | | Extreme pain or discomfort | 4 |  |  | |  |
|  |  | | Prefer not to say | 97 |  |  | |  |
|  |  | | Don’t know | 98 |  |  | |  |

| Q84. | | SHOWCARD 38 **Did you consult any of the health professionals shown on this card in the last four weeks? Read out the category or categories that apply.** MULTICODE OKAY | | | | |  | |
| --- | --- | --- | --- | --- | --- | --- | --- | --- |
|  |  | |  |  |  |  | |  |
|  |  | | Doctor | 1 |  |  | |  |
|  |  | | Nurse | 2 |  |  | |  |
|  |  | | Child birth attendant | 3 |  |  | |  |
|  |  | | Health councillor | 4 |  |  | |  |
|  |  | | Pharmacist | 5 |  |  | |  |
|  |  | | Traditional health worker /Traditional Birth Attendant/Midwife | 6 |  |  | |  |
|  |  | | Traditional healer | 7 |  |  | |  |
|  |  | | Community Health Worker | 8 |  |  | |  |
|  |  | | Other health professionals - INTERVIEWER FIND OUT & WRITE IN | 9 |  |  | |  |
|  |  | | None of these | 10 |  |  | |  |
|  |  | | Prefer not to say | 97 |  |  | |  |
|  |  | | Don’t know | 98 |  |  | |  |

| Q85. | | **Which, if any, of the following health issues have you had in the last four weeks?**  READ OUT A TO M. SINGLE CODE FOR EACH | | | | | | |  | |
| --- | --- | --- | --- | --- | --- | --- | --- | --- | --- | --- |
|  |  | |  | Yes | No | Don’t know |  |  | |  |
|  | a) | | Headache | 1 | 2 | 98 |  |  | |  |
|  | b) | | Loss of appetite | 1 | 2 | 98 |  |  | |  |
|  | c) | | Slept badly | 1 | 2 | 98 |  |  | |  |
|  | d) | | Felt nervous or tense about something | 1 | 2 | 98 |  |  | |  |
|  | e) | | Had trouble thinking clearly | 1 | 2 | 98 |  |  | |  |
|  | f) | | Felt unhappy or sad | 1 | 2 | 98 |  |  | |  |
|  | g) | | Cried more than you would normally | 1 | 2 | 98 |  |  | |  |
|  | h) | | Found it difficult to enjoy daily activities | 1 | 2 | 98 |  |  | |  |
|  | i) | | Had difficulties making decisions | 1 | 2 | 98 |  |  | |  |
|  | j) | | Were less productive than usual | 1 | 2 | 98 |  |  | |  |
|  | k) | | Lost interest in things you would usually enjoy | 1 | 2 | 98 |  |  | |  |
|  | l) | | Felt worthless | 1 | 2 | 98 |  |  | |  |
|  | m) | | Felt tired | 1 | 2 | 98 |  |  | |  |

| Q86. | | **Have you ever thought about taking your own life?**  SINGLE CODE ONLY | | | | |  | |
| --- | --- | --- | --- | --- | --- | --- | --- | --- |
|  |  | |  |  |  |  | |  |
|  |  | | Yes | 1 |  |  | |  |
|  |  | | No | 2 |  |  | |  |
|  |  | | Prefer not to say | 97 |  |  | |  |
|  |  | | Don’t know | 98 |  |  | |  |

ASK Q87 IF YES AT Q86. OTHERS GO TO Q88.

| Q87. | | **And have you ever tried to take your own life?**  SINGLE CODE ONLY | | | | |  | |
| --- | --- | --- | --- | --- | --- | --- | --- | --- |
|  |  | |  |  |  |  | |  |
|  |  | | Yes | 1 |  |  | |  |
|  |  | | No | 2 |  |  | |  |
|  |  | | Prefer not to say | 97 |  |  | |  |
|  |  | | Don’t know | 98 |  |  | |  |

| Q88. | | **In the last 12 months, how much, if anything, have you spent on healthcare for…?**  READ OUT A-C. SINGLE CODE ONLY FOR EACH. IF NOTHING WRITE IN ZERO. | | | | |  | |
| --- | --- | --- | --- | --- | --- | --- | --- | --- |
|  |  | |  |  |  |  | |  |
| a) |  | | **Yourself** (CODE ‘1’ AND WRITE IN) | 1  ☐☐☐☐ |  |  | |  |
| b) |  | | **Your children** (CODE ‘2’ AND WRITE IN) | 2  ☐☐☐☐ |  |  | |  |
| c) |  | | **Other family members living with you** (CODE ‘3’ AND WRITE IN) | 3  ☐☐☐☐ |  |  | |  |

| Q89. | | **Do you use contraception?**  SINGLE CODE ONLY | | | | |  | |
| --- | --- | --- | --- | --- | --- | --- | --- | --- |
|  |  | |  |  |  |  | |  |
|  |  | | Yes | 1 | GO TO Q91 |  | |  |
|  |  | | No | 2 | ASK Q90 |  | |  |
|  |  | | Prefer not to say | 97 | GO TO Q92 |  | |  |
|  |  | | Don’t know | 98 |  |  | |  |

ASK Q90 IF CODE 2 AT Q89. OTHERS GO TO Q91.

| Q90. | | **Why not?**  OPEN QUESTION. SINGLE CODE ONLY | | | | |  | |
| --- | --- | --- | --- | --- | --- | --- | --- | --- |
|  |  | |  |  |  |  | |  |
|  |  | | I don’t want to use it | 1 |  |  | |  |
|  |  | | It is not available in my community | 2 |  |  | |  |
|  |  | | I am prevented from using contraception by my religious beliefs | 3 |  |  | |  |
|  |  | | I am prevented from using contraception by my husband or partner | 4 |  |  | |  |
|  |  | | I am prevented from using contraception by someone else | 5 |  |  | |  |
|  |  | | I cannot afford contraception | 6 |  |  | |  |
|  |  | | I do not know how to access contraception | 7 |  |  | |  |
|  |  | | Other reason - INTERVIEWER FIND OUT & WRITE IN | 8 |  |  | |  |
|  |  | | Prefer not to say | 97 |  |  | |  |

ASK Q91 IF CODE 1 AT Q89. OTHERS GO TO Q92.

| Q91. | | **Whose decision is it to use contraception?**  OPEN QUESTION. SINGLE CODE ONLY | | | | |  | |
| --- | --- | --- | --- | --- | --- | --- | --- | --- |
|  |  | |  |  |  |  | |  |
|  |  | | My decision | 1 |  |  | |  |
|  |  | | Joint decision between myself and my husband or partner | 2 |  |  | |  |
|  |  | | My husband or partner decides | 3 |  |  | |  |
|  |  | | Other people’s decision - INTERVIEWER FIND OUT & WRITE IN | 4 |  |  | |  |
|  |  | | Prefer not to say | 97 |  |  | |  |
|  |  | | Don’t know | 98 |  |  | |  |

ASK ALL

| Q92. | | **Have you ever been pregnant?**  SINGLE CODE ONLY | | | | |  | |
| --- | --- | --- | --- | --- | --- | --- | --- | --- |
|  |  | |  |  |  |  | |  |
|  |  | | Yes | 1 | ASK Q93 |  | |  |
|  |  | | No | 2 | GO TO Q95 |  | |  |
|  |  | | Prefer not to say | 97 |  |  | |  |
|  |  | | Don’t know | 98 |  |  | |  |

ASK Q93 AND Q94 IF YES AT Q92. OTHERS GO TO Q95.

| Q93. | | **How many pregnancies have you had?**  SINGLE CODE ONLY | | | | |  | |
| --- | --- | --- | --- | --- | --- | --- | --- | --- |
|  |  | |  |  |  |  | |  |
|  |  | | CODE ‘1’ AND WRITE IN THE NUMBER | 1  ☐☐ |  |  | |  |
|  |  | | Prefer not to say | 97 |  |  | |  |
|  |  | | Don’t know | 98 |  |  | |  |

| Q94. | | **Have you ever had a pregnancy that miscarried, was aborted or ended in stillbirth?** IF YES ASK **How many miscarriages / abortions / pregnancies that resulted in stillbirth have you experienced.**  INTERVIEWER, ASK AS APPROPRIATE FOR EACH QUESTION A TO C AND WRITE IN THE NUMBER FOR EACH. | | | | |  | |
| --- | --- | --- | --- | --- | --- | --- | --- | --- |
|  |  | |  |  |  |  | |  |
|  | a) | | Yes - miscarried (CODE ‘1’ AND WRITE IN THE NUMBER) | 1  ☐☐ |  |  | |  |
|  | b) | | Yes - was aborted (CODE ‘2’ AND WRITE IN THE NUMBER) | 2  ☐☐ |  |  | |  |
|  | c) | | Yes - resulted in stillbirth (CODE ‘3’ AND WRITE IN THE NUMBER) | 3  ☐☐ |  |  | |  |
|  |  | | I have never miscarried | 4 |  |  | |  |
|  |  | | No pregnancies aborted | 5 |  |  | |  |
|  |  | | No stillbirths | 6 |  |  | |  |
|  |  | | Prefer not to say | 97 |  |  | |  |
|  |  | | Don’t know | 98 |  |  | |  |

ASK ALL

| Q95. | | **Do you have school-age children living with you?**  SINGLE CODE ONLY | | | | |  | |
| --- | --- | --- | --- | --- | --- | --- | --- | --- |
|  |  | |  |  |  |  | |  |
|  |  | | Yes | 1 | ASK Q96 |  | |  |
|  |  | | No | 2 | GO TO Q101 |  | |  |
|  |  | | Prefer not to say | 97 |  |  | |  |
|  |  | | Don’t know | 98 |  |  | |  |

ASK Q96 IF CODE 1 AT Q95. OTHERS GO TO Q101 IN THE NEXT SECTION.

| Q96. | | **Are all your school-aged children currently attending school?**  SINGLE CODE ONLY | | | | |  | |
| --- | --- | --- | --- | --- | --- | --- | --- | --- |
|  |  | |  |  |  |  | |  |
|  |  | | Yes | 1 | GO TO Q99 |  | |  |
|  |  | | No | 2 | ASK Q97 |  | |  |
|  |  | | Don’t know | 3 | GO TO Q99 |  | |  |
|  |  | | Prefer not to say | 97 |  |  | |  |

ASK Q97 IF CODE ‘2’ AT Q96. OTHERS GO TO Q99

| Q97. | | **Why do some or all of your school-aged children not attend school?**  MULTICODE OKAY | | | | |
| --- | --- | --- | --- | --- | --- | --- |
|  |  | | Distance to school to great to attend | 1 |  |  |
|  |  | | They do not want to attend | 2 |  |  |
|  |  | | Cannot afford to attend | 3 |  |  |
|  |  | | Too busy working in the household | 4 |  |  |
|  |  | | Too busy working outside the household | 5 |  |  |
|  |  | | We do not see the point in our children attending school | 6 |  |  |
|  |  | | Too dangerous to attend | 7 |  |  |
|  |  | | Prevented from attending | 8 |  |  |
|  |  | | Prefer not to say | 97 |  |  |

ASK Q98 IF CODE ‘8’ AT Q97. OTHERS GO TO Q99.

| Q98. | | **Who prevented your school-aged child or children from attending school?**  MULTICODE OKAY | | | | |
| --- | --- | --- | --- | --- | --- | --- |
|  |  | |  |  |  |  |
|  |  | | Husband or partner | 1 |  |  |
|  |  | | Other family member | 2 |  |  |
|  |  | | Members of the community | 3 |  |  |
|  |  | | Someone else (SPECIFY) | 4 |  |  |
|  |  | | Prefer not to say | 97 |  |  |

ASK Q99 IF WOMAN HAS SCHOOL-AGED CHILDREN LIVING WITH HER (CODE ‘1’ AT Q95). OTHERS GO TO Q101 IN THE NEXT SECTION.

| Q99. | | | **Starting with the oldest child of school-age, tell me:**  A: **Their gender?**  SELECT M FOR MALE OR F FOR FEMALE  B: **What is their grade at school?**  WRITE IN GRADE IN THE SECOND COLUMN  C: **How much, if anything, did you pay in school fees for this child in the last academic year?**  WRITE IN LOCAL CURRENCY IN THE THIRD COLUMN  D: **How much, if anything, did you pay for school transport for this child in the last 12 months?**  WRITE IN LOCAL CURRENCY IN THE FOURTH COLUMN  E: **How much, if anything, did you pay for other school expenses such as school uniforms, books and additional fees in the last 12 months?**  WRITE IN LOCAL CURRENCY IN THE LAST COLUMN  ENUMERATOR: REPEAT THE QUESTIONS FOR THE NEXT CHILD AND SO ON UNTIL THE QUESTIONS HAVE BEEN ASKED FOR ALL THE CHILDREN OF SCHOOL AGE IN THE HOUSEHOLD | | | | | | |  | | |
| --- | --- | --- | --- | --- | --- | --- | --- | --- | --- | --- | --- | --- |
|  |  |  | |  | A.  Gender | B.  GRADE | C.  School Fees | D.  TransPORT | E. Books/ Uniforms | |  |  |
|  |  | Child One | | 1 | M f | ☐☐ | ☐☐☐ | ☐☐☐ | ☐☐☐ | |  |  |
|  |  | Child Two | | 2 | M f | ☐☐ | ☐☐☐ | ☐☐☐ | ☐☐☐ | |  |  |
|  |  | Child Three | | 3 | M f | ☐☐ | ☐☐☐ | ☐☐☐ | ☐☐☐ | |  |  |
|  |  | Child Four | | 4 | M f | ☐☐ | ☐☐☐ | ☐☐☐ | ☐☐☐ | |  |  |
|  |  | Child Five | | 5 | M f | ☐☐ | ☐☐☐ | ☐☐☐ | ☐☐☐ | |  |  |
|  |  | Child Six | | 6 | M f | ☐☐ | ☐☐☐ | ☐☐☐ | ☐☐☐ | |  |  |
|  |  | Child Seven | | 7 | M f | ☐☐ | ☐☐☐ | ☐☐☐ | ☐☐☐ | |  |  |
|  |  | Child Eight | | 8 | M f | ☐☐ | ☐☐☐ | ☐☐☐ | ☐☐☐ | |  |  |
|  |  | Child Nine | | 9 | M f | ☐☐ | ☐☐☐ | ☐☐☐ | ☐☐☐ | |  |  |
|  |  | Child Ten | | 10 | M f | ☐☐ | ☐☐☐ | ☐☐☐ | ☐☐☐ | |  |  |
|  |  | Child Eleven | | 11 | M f | ☐☐ | ☐☐☐ | ☐☐☐ | ☐☐☐ | |  |  |
|  |  | Child Twelve | | 12 | M f | ☐☐ | ☐☐☐ | ☐☐☐ | ☐☐☐ | |  |  |
|  |  | Prefer not to say | | 97 |  | | | | | |  |  |

| Q100. | | **Did any of your children fail or receive poor grades or marks in the last academic year? Base your answer on the last full academic year.**  CODE ONE RESPONSE FOR EACH CHILD. SINGLE CODE FOR EACH | | | | | | |  | |
| --- | --- | --- | --- | --- | --- | --- | --- | --- | --- | --- |
|  |  | |  | Yes | No | Prefer not to say | Don’t know |  | |  |
|  | a) | | Child One | 1 | 2 | 97 | 98 |  | |  |
|  | b) | | Child Two | 1 | 2 | 97 | 98 |  | |  |
|  | c) | | Child Three | 1 | 2 | 97 | 98 |  | |  |
|  | d) | | Child Four | 1 | 2 | 97 | 98 |  | |  |
|  | e) | | Child Five | 1 | 2 | 97 | 98 |  | |  |
|  | f) | | Child Six | 1 | 2 | 97 | 98 |  | |  |
|  | g) | | Child Seven | 1 | 2 | 97 | 98 |  | |  |
|  | h) | | Child Eight | 1 | 2 | 97 | 98 |  | |  |
|  | i) | | Child Nine | 1 | 2 | 97 | 98 |  | |  |
|  | j) | | Child Ten | 1 | 2 | 97 | 98 |  | |  |
|  | k) | | Child Eleven | 1 | 2 | 97 | 98 |  | |  |
|  | l) | | Child Twelve | 1 | 2 | 97 | 98 |  | |  |

ASK ALL

| HISTORY OF VIOLENCE IN THE HOME |
| --- |

| Q101a. | | **Please remind me - do you have a current husband or partner, or have had a husband or partner in the last 12 months?**  SINGLE CODE ONLY | | | | |  | |
| --- | --- | --- | --- | --- | --- | --- | --- | --- |
|  |  | |  |  |  |  | |  |
|  |  | | Yes | 1 | ASK Q101b |  | |  |
|  |  | | No | 2 | GO TO Q141 |  | |  |
|  |  | | Prefer not to say | 97 |  |  | |  |
|  |  | | Don’t know | 98 |  |  | |  |

CONTINUE TO Q101B IF THE WOMAN OR GIRL HAS A HUSBAND OR PARTNER OR HAS HAD A HUSBAND OR PARTNER IN THE LAST 12 MONTHS. THIS IS CODE ‘1’ AT Q101a.

GO TO Q141 IF THE WOMAN OR GIRL DOES NOT HAVE A HUSBAND OR PARTNER OR HAS NOT HAD A HUSBAND OR PARTNER IN THE LAST 12 MONTHS.

**In relationships with other people we often have disagreements, fights or quarrels. We might also experience behaviours that can hurt us physically or emotionally. I want to ask you about any such behaviours your current or previous husband or partner may have done to you.**

| Q101b | | | SHOWCARD 39 **In the last 12 months, how many times, if any, did your current or previous husband or partner do any of the following things?**  READ OUT A TO G. SINGLE CODE FOR EACH | | | | | | |  | |  | |  | | |
| --- | --- | --- | --- | --- | --- | --- | --- | --- | --- | --- | --- | --- | --- | --- | --- | --- |
|  |  |  | | Never | Once or twice | Between 3 and 5 times | Between 6 and 10 times | Between 11 and 20 times | More than 20 times | | Prefer not to say | | Don’t know | | Not applic-able |  |
|  | a) | Prevent you from getting a job, going to work, trading or earning money | | 1 | 2 | 3 | 4 | 5 | 6 | | 97 | | 98 | | 99 |  |
|  | b) | Take your earnings against your will | | 1 | 2 | 3 | 4 | 5 | 6 | | 97 | | 98 | | 99 |  |
|  | c) | Spend money on alcohol, tobacco or other things for himself when he knew you did not have enough for essential household expenses | | 1 | 2 | 3 | 4 | 5 | 6 | | 97 | | 98 | | 99 |  |
|  | d) | Give you less money than you needed to pay a household bill | | 1 | 2 | 3 | 4 | 5 | 6 | | 97 | | 98 | | 99 |  |
|  | e) | Withdraw money from your personal account without your permission | | 1 | 2 | 3 | 4 | 5 | 6 | | 97 | | 98 | | 99 |  |
|  | f) | Take goods or food from home and sell without your permission | | 1 | 2 | 3 | 4 | 5 | 6 | | 97 | | 98 | | 99 |  |
|  | g) | Force you to work against your will | | 1 | 2 | 3 | 4 | 5 | 6 | | 97 | | 98 | | 99 |  |

| Q102 | | SHOWCARD 40 **In the last 12 months, how many times, if any, did your current or previous husband or partner ever do any of the following things?**  READ OUT A TO I. SINGLE CODE FOR EACH | | | | | | | |  | |  | |  | |
| --- | --- | --- | --- | --- | --- | --- | --- | --- | --- | --- | --- | --- | --- | --- | --- |
|  |  |  | Never | Once or twice | Between 3 and 5 times | Between 6 and 10 times | Between 11 and 20 times | More than 20 times | Prefer not to say | | Don’t know | | Not applicable | |  |
|  | a) | Insult you or make you feel bad? | 1 | 2 | 3 | 4 | 5 | 6 | 97 | | 98 | | 99 | |  |
|  | b) | Belittle or humiliate you in front of other people? | 1 | 2 | 3 | 4 | 5 | 6 | 97 | | 98 | | 99 | |  |
|  | c) | Do things to scare or frighten you? | 1 | 2 | 3 | 4 | 5 | 6 | 97 | | 98 | | 99 | |  |
|  | d) | Threaten to hurt you or someone you care about? | 1 | 2 | 3 | 4 | 5 | 6 | 97 | | 98 | | 99 | |  |

| Q103. | | SHOWCARD 41 **And still thinking about** **the last 12 months, how many times, if any, did your current or previous husband or partner ever do any of these things?** READ OUT A TO I. SINGLE CODE FOR EACH | | | | | | | |  | |  | |  | |
| --- | --- | --- | --- | --- | --- | --- | --- | --- | --- | --- | --- | --- | --- | --- | --- |
|  |  |  | Never | Once or twice | Between 3 and 5 times | Between 6 and 10 times | Between 11 and 20 times | More than 20 times | Prefer not to say | | Don’t know | | Not applicable | |  |
|  | a) | Slap you or throw something at you which could hurt you | 1 | 2 | 3 | 4 | 5 | 6 | 97 | | 98 | | 99 | |  |
|  | b) | Push or shove you | 1 | 2 | 3 | 4 | 5 | 6 | 97 | | 98 | | 99 | |  |
|  | c) | Hit you with a fist or with something else which could hurt you | 1 | 2 | 3 | 4 | 5 | 6 | 97 | | 98 | | 99 | |  |
|  | d) | Kick, drag, beat, choke or burn you | 1 | 2 | 3 | 4 | 5 | 6 | 97 | | 98 | | 99 | |  |
|  | e) | Threaten to use or actually use a gun, knife or other weapon against you | 1 | 2 | 3 | 4 | 5 | 6 | 97 | | 98 | | 99 | |  |
|  | f) | Throw you out of the house | 1 | 2 | 3 | 4 | 5 | 6 | 97 | | 98 | | 99 | |  |
|  | g) | Physically force you to have sex when you did not want to | 1 | 2 | 3 | 4 | 5 | 6 | 97 | | 98 | | 99 | |  |
|  | h) | Used threats or intimidation to get you to have sex when you did not want to | 1 | 2 | 3 | 4 | 5 | 6 | 97 | | 98 | | 99 | |  |
|  | i) | Force you to do something else sexual that you did not want to | 1 | 2 | 3 | 4 | 5 | 6 | 97 | | 98 | | 99 | |  |

| Q104. | | SHOWCARD 42 **And before the last 12 months, how many times, if any, did your current or previous husband or partner ever do any of these things?**  READ OUT A TO I. SINGLE CODE FOR EACH | | | | | | |  |  |
| --- | --- | --- | --- | --- | --- | --- | --- | --- | --- | --- |
|  |  |  | Never | Once | A few times | Many times | Prefer not to say | Don’t know | | Not applicable |
|  | a) | Slap you or throw something at you which could hurt you | 1 | 2 | 3 | 4 | 97 | 98 | | 99 |
|  | b) | Push or shove you | 1 | 2 | 3 | 4 | 97 | 98 | | 99 |
|  | c) | Hit you with a fist or with something else which could hurt you | 1 | 2 | 3 | 4 | 97 | 98 | | 99 |
|  | d) | Kick, drag, beat, choke or burn you | 1 | 2 | 3 | 4 | 97 | 98 | | 99 |
|  | e) | Threaten to use or actually use a gun, knife or other weapon against you | 1 | 2 | 3 | 4 | 97 | 98 | | 99 |
|  | f) | Throw you out of the house | 1 | 2 | 3 | 4 | 97 | 98 | | 99 |
|  | g) | Physically force you to have sex when you did not want to | 1 | 2 | 3 | 4 | 97 | 98 | | 99 |
|  | h) | Used threats or intimidation to get you to have sex when you did not want to | 1 | 2 | 3 | 4 | 97 | 98 | | 99 |
|  | i) | Force you to do something else sexual that you did not want to | 1 | 2 | 3 | 4 | 97 | 98 | | 99 |

ASK Q105 IF ONE OR MORE INCIDENTS CODED AS 2-6 AT Q101b, AND/OR Q102, AND/OR Q103.

IF THE WOMAN OR GIRL HAS ANSWERED ‘NEVER’ (CODE 1), ‘DON’T KNOW’ (CODE 98), PREFER NOT TO SAY (CODE 97) OR ‘NOT APPLICABLE’ (CODE 99) TO ALL OF QUESTIONS Q101b TO Q103 THEN GO TO Q141 IN THE NEXT SECTION

**Now I wish to ask more details about your experiences of behaviours by your current or previous husband or partner in the last 12 months**.

| Q105. | | **Thinking about the different behaviours you have reported that your current or previous husband or partner has done in the last 12 months, how many incidents of this nature do you remember? This may be a single behaviour or combination of behaviours reported above.** SINGLE CODE ONLY | | | | |
| --- | --- | --- | --- | --- | --- | --- |
|  |  | |  |  |  |  |
|  |  | | CODE ‘1’ AND WRITE IN THE NUMBER OF INCIDENTS | 1  ☐☐☐ |  |  |
|  |  | | None | 2 |  |  |
|  |  | | Prefer not to say | 97 |  |  |
|  |  | | Don’t know | 98 |  |  |

| Q106. | | SHOWCARD 43 **As a result of any of these incidences, did you sustain any injuries? Read out the categories that apply.**  MULTICODE OKAY | | | | | | |  | |
| --- | --- | --- | --- | --- | --- | --- | --- | --- | --- | --- |
|  |  | |  |  |  |  |  |  | |  |
|  |  | | Puncture wounds or bite marks | 1 |  |  |  |  | |  |
|  |  | | Scratches or bruises | 2 |  |  |  |  | |  |
|  |  | | Sprains or dislocations | 3 |  |  |  |  | |  |
|  |  | | Burns | 4 |  |  |  |  | |  |
|  |  | | Cuts, gashes or bleeding | 5 |  |  |  |  | |  |
|  |  | | Eye injury | 6 |  |  |  |  | |  |
|  |  | | Fractures of broken bones | 7 |  |  |  |  | |  |
|  |  | | Broken eardrum | 8 |  |  |  |  | |  |
|  |  | | Other injuries - INTERVIEWER FIND OUT & WRITE IN | 9 |  |  |  |  | |  |
|  |  | | None of these | 11 |  |  |  |  | |  |
|  |  | | Prefer not to say | 97 |  |  |  |  | |  |
|  |  | | Don’t know | 98 |  |  |  |  | |  |

| Q107. | | **Did you require or seek any healthcare as a result of any injuries caused by your husband or partner in the last 12 months?**  SINGLE CODE ONLY | | | | |  | |
| --- | --- | --- | --- | --- | --- | --- | --- | --- |
|  |  | |  |  |  |  | |  |
|  |  | | Yes | 1 | ASK Q108 |  | |  |
|  |  | | No | 2 | GO TO Q109 |  | |  |
|  |  | | Prefer not to say | 97 |  |  | |  |
|  |  | | Don’t know | 98 |  |  | |  |

ASK Q108 IF YES AT Q107. OTHERS GO TO Q109.

| Q108. | | **How much, if any, money did you have to spend on healthcare as a result of injuries caused by your partner or husband in the last 12 months? This includes paying the doctor or paying for medicine, lab tests.**  SINGLE CODE ONLY | | | | |  | |
| --- | --- | --- | --- | --- | --- | --- | --- | --- |
|  |  | |  |  |  |  | |  |
|  |  | | CODE ‘1’ AND WRITE IN THE AMOUNT IN LOCAL CURRENCY for Doctor fees | 1  ☐☐☐☐ |  |  | |  |
|  |  | | CODE ‘2’ AND WRITE AMOUNT IN LOCAL CURRENCY for medicines | 2  ☐☐☐☐ |  |  | |  |
|  |  | | CODE ‘3’ AND WRITE AMOUNT IN LOCAL CURRENCY for lab tests including x-rays, ultrasound | 3  ☐☐☐☐ |  |  | |  |
|  |  | | None | 4 |  |  | |  |
|  |  | | Prefer not to say | 97 |  |  | |  |
|  |  | | Don’t know | 98 |  |  | |  |

ASK Q109 IF THE WOMAN OR GIRL HAS EXPERIENCED ANY VIOLENCE FROM CURRENT OR PREVIOUS HUSBAND OR PARTNER (THIS IS CODE ‘1’ AT Q105). OTHERS GO TO Q110.

| Q109. | | **And how much, if anything, did you spend on transport to and from the hospital or health clinic as a result of injuries caused by your partner or husband in the last 12 months?**  SINGLE CODE ONLY | | | | |  | |
| --- | --- | --- | --- | --- | --- | --- | --- | --- |
|  |  | |  |  |  |  | |  |
|  |  | | CODE ‘1’ AND WRITE IN THE AMOUNT IN LOCAL CURRENCY | 1  ☐☐ |  |  | |  |
|  |  | | None | 2 |  |  | |  |
|  |  | | Prefer not to say | 97 |  |  | |  |
|  |  | | Don’t know | 98 |  |  | |  |

ASK Q110 IF THE WOMAN OR GIRL WORKS (CODE 1 AT Q57A). OTHERS GO TO Q113.

| Q110. | | **Were you required to take any time off work as a result of these incidents**  **in the last 12 months?**  SINGLE CODE ONLY | | | | |  | |
| --- | --- | --- | --- | --- | --- | --- | --- | --- |
|  |  | |  |  |  |  | |  |
|  |  | | Yes | 1 | ASK Q111 |  | |  |
|  |  | | No | 2 | GO TO Q113 |  | |  |
|  |  | | Prefer not to say | 97 |  |  | |  |
|  |  | | Don’t know | 98 |  |  | |  |

ASK Q111 IF YES AT Q110. OTHERS GO TO Q113.

| Q111. | | **How many days of work did you miss as a result of these incidents in the last 12 months?**  SINGLE CODE ONLY | | | | |  | |
| --- | --- | --- | --- | --- | --- | --- | --- | --- |
|  |  | |  |  |  |  | |  |
|  |  | | WRITE IN NUMBER OF DAYS | 1  ☐☐☐ |  |  | |  |
|  |  | | Prefer not to say | 97 |  |  | |  |
|  |  | | Don’t know | 98 |  |  | |  |

ASK Q112 IF CODE 1 AT Q111. OTHERS GO TO Q113.

| Q112. | | **And of the days you missed work because of these incidents in the last 12 months, did you get paid for those days that you missed?**  IF YES ASK **Was that for all of the days or some of the days?**  SINGLE CODE ONLY | | | | |  | |
| --- | --- | --- | --- | --- | --- | --- | --- | --- |
|  |  | |  |  |  |  | |  |
|  |  | | Yes – all of these days | 1 |  |  | |  |
|  |  | | Yes – some of these days if so how many CODE ‘2’ AND WRITE IN NUMBER OF DAYS | 2  ☐☐☐ |  |  | |  |
|  |  | | No – not paid for any of the days missed | 3 |  |  | |  |
|  |  | | Prefer not to say | 97 |  |  | |  |
|  |  | | Don’t know | 98 |  |  | |  |

ASK Q113 IF THE WOMAN OR GIRL HAS EXPERIENCED ANY VIOLENCE FROM CURRENT OR PREVIOUS HUSBAND OR PARTNER (THIS IS CODE ‘1’ AT Q105).

| Q113. | | **Did you fully or partially stop any of the following tasks as a result of these incidents in the last 12 months?**  READ OUT A TO N. SINGLE CODE FOR EACH.  IF CODE 1 OR CODE 2 USED, ASK NUMBER OF DAYS AND WRITE IN. | | | | | | |  | |
| --- | --- | --- | --- | --- | --- | --- | --- | --- | --- | --- |
|  |  | |  | Fully Stopped | Days | Partially stopped (50%) | Days | No Effect | |  |
|  | a) | | Fetching water | 1 | ☐☐☐ | 2 | ☐☐☐ | 99 | |  |
|  | b) | | Fetching wood | 1 | ☐☐☐ | 2 | ☐☐☐ | 99 | |  |
|  | c) | | Caring for children | 1 | ☐☐☐ | 2 | ☐☐☐ | 99 | |  |
|  | d) | | Ironing | 1 | ☐☐☐ | 2 | ☐☐☐ | 99 | |  |
|  | e) | | Washing clothes | 1 | ☐☐☐ | 2 | ☐☐☐ | 99 | |  |
|  | f) | | Sweeping | 1 | ☐☐☐ | 2 | ☐☐☐ | 99 | |  |
|  | g) | | Washing dishes | 1 | ☐☐☐ | 2 | ☐☐☐ | 99 | |  |
|  | h) | | Washing vehicles | 1 | ☐☐☐ | 2 | ☐☐☐ | 99 | |  |
|  | i) | | Disposing garbage | 1 | ☐☐☐ | 2 | ☐☐☐ | 99 | |  |
|  | j) | | Cooking | 1 | ☐☐☐ | 2 | ☐☐☐ | 99 | |  |
|  | k) | | Shopping for household needs | 1 | ☐☐☐ | 2 | ☐☐☐ | 99 | |  |
|  | l) | | Running errands | 1 | ☐☐☐ | 2 | ☐☐☐ | 99 | |  |
|  | m) | | Taking care of livestock or poultry | 1 | ☐☐☐ | 2 | ☐☐☐ | 99 | |  |
|  | n) | | Making clothes for family | 1 | ☐☐☐ | 2 | ☐☐☐ | 99 | |  |

ASK Q114 AND Q115 IF THE WOMAN OR GIRL HAS SCHOOL-AGE CHILDREN (CODE 1 AT Q95). IF NOT, GO TO Q116.

| Q114. | | SHOWCARD 44 **Which, if any, of these things happened to your children as a result of these incidents in the last 12 months?**  MULTICODE OKAY | | | | |  | |
| --- | --- | --- | --- | --- | --- | --- | --- | --- |
|  |  | |  |  |  |  | |  |
|  |  | | Felt scared | 1 |  |  | |  |
|  |  | | Felt confused | 2 |  |  | |  |
|  |  | | Asked lots of questions | 3 |  |  | |  |
|  |  | | Wet the bed | 4 |  |  | |  |
|  |  | | Had nightmares | 5 |  |  | |  |
|  |  | | Physically shook | 6 |  |  | |  |
|  |  | | Did not want to play | 7 |  |  | |  |
|  |  | | Other - INTERVIEWER FIND OUT & WRITE IN) | 8 |  |  | |  |
|  |  | | None of these | 9 |  |  | |  |
|  |  | | Prefer not to say | 97 |  |  | |  |
|  |  | | Don’t know | 98 |  |  | |  |

| Q115. | | **How many days of school did your children miss as a result of these incidents in the last 12 months?**  SINGLE CODE ONLY | | | | |
| --- | --- | --- | --- | --- | --- | --- |
|  |  | |  |  |  |  |
|  |  | | CODE ‘1’ AND WRITE IN THE NUMBER OF DAYS | 1  ☐☐☐ |  |  |
|  |  | | Prefer not to say | 97 |  |  |
|  |  | | Don’t know | 98 |  |  |

ASK Q116 IF THE WOMAN OR GIRL HAS EXPERIENCED ANY VIOLENCE FROM CURRENT OR PREVIOUS HUSBAND OR PARTNER (THIS IS CODE ‘1’ AT Q105). OTHERS GO TO Q117.

| Q116. | | **Did you report any of these incidents to the police?**  SINGLE CODE ONLY | | | | |  | |
| --- | --- | --- | --- | --- | --- | --- | --- | --- |
|  |  | |  |  |  |  | |  |
|  |  | | Yes | 1 | ASK Q117 |  | |  |
|  |  | | No | 2 | GO TO Q127 |  | |  |
|  |  | | Prefer not to say | 97 |  |  | |  |
|  |  | | Don’t know | 98 |  |  | |  |

ASK Q117 IF YES (CODE ‘1’) AT Q116. OTHERS GO TO Q127

| Q117. | | **Did it cost any money to file a complaint?**  SINGLE CODE ONLY | | | | |
| --- | --- | --- | --- | --- | --- | --- |
|  |  | |  |  |  |  |
|  |  | | Yes | 1 | ASK Q118 |  |
|  |  | | No | 2 | GO TO Q119 |  |
|  |  | | Prefer not to say | 97 |  |  |
|  |  | | Don’t know | 98 |  |  |

ASK Q118 IF YES (CODE ‘1’ AT Q117). OTHERS GO TO Q119

| Q118. | | **And how much did it cost you to file a complaint?**  SINGLE CODE ONLY | | | | |
| --- | --- | --- | --- | --- | --- | --- |
|  |  | |  |  |  |  |
|  |  | | CODE ‘1’ AND WRITE IN THE AMOUNT IN LOCAL CURRENCY | 1  ☐☐☐☐ |  |  |
|  |  | | Prefer not to say | 97 |  |  |
|  |  | | Don’t know | 98 |  |  |

| Q119. | | **Did you have to pay the police any money apart from a filing fee?**  SINGLE CODE ONLY | | | | |  | |
| --- | --- | --- | --- | --- | --- | --- | --- | --- |
|  |  | |  |  |  |  | |  |
|  |  | | Yes | 1 | ASK Q120 |  | |  |
|  |  | | No | 2 | GO TO Q121 |  | |  |
|  |  | | Prefer not to say | 97 |  |  | |  |
|  |  | | Don’t know | 98 |  |  | |  |

ASK Q120 IF YES (CODE ‘1’) AT Q119. OTHERS GO TO Q121.

| Q120 | | **And how much did you have to pay the police?**  SINGLE CODE ONLY | | | | |  | |
| --- | --- | --- | --- | --- | --- | --- | --- | --- |
|  |  | |  |  |  |  | |  |
|  |  | | CODE ‘1’ AND WRITE IN AMOUNT IN LOCAL CURRENCY | 1  ☐☐☐☐ |  |  | |  |
|  |  | | Prefer not to say | 97 |  |  | |  |
|  |  | | Don’t know | 98 |  |  | |  |

| Q121. | | **Did you incur any transport costs going to the police?**  SINGLE CODE ONLY | | | | |
| --- | --- | --- | --- | --- | --- | --- |
|  |  | |  |  |  |  |
|  |  | | Yes | 1 | ASK Q122 |  |
|  |  | | No | 2 | GO TO Q123 |  |
|  |  | | Prefer not to say | 97 |  |  |
|  |  | | Don’t know | 98 |  |  |

ASK Q122 IF YES (CODE ‘1’ AT Q121). OTHERS GO TO Q123.

| Q122. | | **And how much did you spend on transport going to the police?**  SINGLE CODE ONLY | | | | |
| --- | --- | --- | --- | --- | --- | --- |
|  |  | |  |  |  |  |
|  |  | | CODE ‘1’ AND WRITE IN AMOUNT IN LOCAL CURRENCY | 1  ☐☐☐☐ |  |  |
|  |  | | Prefer not to say | 97 |  |  |
|  |  | | Don’t know | 98 |  |  |

| Q123. | | **Did the complaint go to court?**  SINGLE CODE ONLY | | | | |  | |
| --- | --- | --- | --- | --- | --- | --- | --- | --- |
|  |  | |  |  |  |  | |  |
|  |  | | Yes | 1 | ASK Q124 |  | |  |
|  |  | | No | 2 | GO TO Q127 |  | |  |
|  |  | | Prefer not to say | 97 |  |  | |  |
|  |  | | Don’t know | 98 |  |  | |  |

ASK Q124 IF YES AT Q123 (CODE ‘1’). OTHERS GO TO Q127.

| Q124. | | **Did it cost you any money to file the case in court?**  SINGLE CODE ONLY | | | | |  | |
| --- | --- | --- | --- | --- | --- | --- | --- | --- |
|  |  | |  |  |  |  | |  |
|  |  | | Yes | 1 | ASK Q125 |  | |  |
|  |  | | No | 2 | GO TO Q127 |  | |  |
|  |  | | Prefer not to say | 97 |  |  | |  |
|  |  | | Don’t know | 98 |  |  | |  |

ASK Q125 IF YES (CODE ‘1’) AT Q124. OTHERS GO TO Q127.

| Q125. | | **And how much did it cost you to go to court? This may include, for example, lawyers’ fees.**  SINGLE CODE ONLY | | | | |  | |
| --- | --- | --- | --- | --- | --- | --- | --- | --- |
|  |  | |  |  |  |  | |  |
|  |  | | CODE ‘1’ AND WRITE IN THE AMOUNT IN LOCAL CURRENCY | 1  ☐☐☐☐ |  |  | |  |
|  |  | | None | 2 |  |  | |  |
|  |  | | Prefer not to say | 97 |  |  | |  |
|  |  | | Don’t know | 98 |  |  | |  |

| Q126. | | **And how much did you spend on transport going to court?**  SINGLE CODE ONLY | | | |
| --- | --- | --- | --- | --- | --- |
|  |  | |  |  |  |
|  |  | | CODE ‘1’ AND WRITE IN AMOUNT IN LOCAL CURRENCY | 1  ☐☐☐ |  |
|  |  | | None | 2 |  |
|  |  | | Prefer not to say | 97 |  |
|  |  | | Don’t know | 98 |  |

| Q127. | | SHOWCARD 45 **Thinking about all of the financial costs we just discussed as a result of these incidents in the last 12 months. Who paid these costs? Read out the statement or statements that apply.**  MULTICODE OKAY | | | | |  | |
| --- | --- | --- | --- | --- | --- | --- | --- | --- |
|  |  | |  |  |  |  | |  |
|  |  | | I paid with my own money | 1 |  |  | |  |
|  |  | | My husband/partner paid with his own money | 2 |  |  | |  |
|  |  | | My husband/partner and I jointly paid the costs out of household money | 3 |  |  | |  |
|  |  | | Other people paid the financial costs - INTERVIEWER FIND OUT & WRITE IN | 4 |  |  | |  |
|  |  | | Prefer not to say | 97 |  |  | |  |
|  |  | | Don’t know | 98 |  |  | |  |

| Q128 | | **Thinking about these incidents of violence from your current or previous husband or partner in the last 12 months, did you leave home and seek shelter somewhere else?**  SINGLE CODE ONLY | | | | |  | |
| --- | --- | --- | --- | --- | --- | --- | --- | --- |
|  |  | |  |  |  |  | |  |
|  |  | | Yes – left home or sought shelter | 1 | ask q129 |  | |  |
|  |  | | No – did not leave home | 2 | go to q133 |  | |  |
|  |  | | Prefer not to say | 97 |  |  | |  |
|  |  | | Don’t know | 98 |  |  | |  |

ASK Q129 TO Q133 IF YES (CODE 1) AT Q128. OTHERS GO TO Q133.

| Q129. | | **Where did you go?**  MULTICODE OKAY | | | | |  | |
| --- | --- | --- | --- | --- | --- | --- | --- | --- |
|  |  | |  |  |  |  | |  |
|  |  | | Stayed with birth family | 1 |  |  | |  |
|  |  | | Stayed with friends | 2 |  |  | |  |
|  |  | | Stayed in a shelter | 3 |  |  | |  |
|  |  | | I stayed somewhere else - INTERVIEWER FIND OUT & WRITE IN) | 4 |  |  | |  |
|  |  | | Prefer not to say | 97 |  |  | |  |
|  |  | | Don’t know | 98 |  |  | |  |

| Q130. | | **How many days did you stay away?**  MULTICODE OKAY | | | | |  | |
| --- | --- | --- | --- | --- | --- | --- | --- | --- |
|  |  | |  |  |  |  | |  |
|  |  | | CODE ‘1’ AND WRITE IN THE NUMBER OF DAYS | 1  ☐☐ |  |  | |  |
|  |  | | I am still away | 2 |  |  | |  |
|  |  | | Prefer not to say | 97 |  |  | |  |
|  |  | | Don’t know | 98 |  |  | |  |

| Q131. | | **Did you have to pay any money to stay away, for example to pay for accommodation, food, etc.?**  SINGLE CODE ONLY | | | | |  | |
| --- | --- | --- | --- | --- | --- | --- | --- | --- |
|  |  | |  |  |  |  | |  |
|  |  | | Yes  CODE ‘1’ AND WRITE AMOUNT IN LOCAL CURRENCY | 1  ☐☐☐☐ |  |  | |  |
|  |  | | No | 2 |  |  | |  |
|  |  | | Prefer not to say | 97 |  |  | |  |
|  |  | | Don’t know | 98 |  |  | |  |

| Q132. | | SHOWCARD 46 **If you returned home, why did you do so?**  MULTICODE OKAY | | | | |
| --- | --- | --- | --- | --- | --- | --- |
|  |  | |  |  |  |  |
|  |  | | I wanted to do so | 1 |  |  |
|  |  | | I felt like I should do so | 2 |  |  |
|  |  | | Did not have any money | 3 |  |  |
|  |  | | My parents/birth family said that I should return | 4 |  |  |
|  |  | | Community leaders/religious leaders persuaded me to return | 5 |  |  |
|  |  | | My children need their mother | 6 |  |  |
|  |  | | My husband asked me to return | 7 |  |  |
|  |  | | My husband forced me to return | 8 |  |  |
|  |  | | My husband’s birth family forced me to return | 9 |  |  |
|  |  | | Other reason - INTERVIEWER FIND OUT & WRITE IN | 10 |  |  |
|  |  | | Prefer not to say | 97 |  |  |
|  |  | | Don’t know | 98 |  |  |

ASK Q133 IF THE WOMAN OR GIRL HAS EXPERIENCED ANY VIOLENCE FROM CURRENT OR PREVIOUS HUSBAND OR PARTNER (CODE ‘1’ AT Q105). OTHERS GO TO Q136.

| Q133. | | **As a result of violent incidents with your current or previous husband or partner, did you have to replace any property?**  SINGLE CODE ONLY | | | | |  | |
| --- | --- | --- | --- | --- | --- | --- | --- | --- |
|  |  | |  |  |  |  | |  |
|  |  | | Yes | 1 | ASK Q134 |  | |  |
|  |  | | No | 2 | GO TO Q136 |  | |  |
|  |  | | Prefer not to say | 97 |  |  | |  |
|  |  | | Don’t know | 98 |  |  | |  |

ASK Q134 AND Q135 IF YES (CODE ‘1’ AT Q133). OTHERS GO TO Q136.

| Q134. | | **What kind of property was it?**  MULTICODE OKAY | | | | |  | |
| --- | --- | --- | --- | --- | --- | --- | --- | --- |
|  |  | |  |  |  |  | |  |
|  |  | | Dishes, pots or utensils | 1 |  |  | |  |
|  |  | | Furniture | 2 |  |  | |  |
|  |  | | Television or radio | 3 |  |  | |  |
|  |  | | Repairs to the house | 4 |  |  | |  |
|  |  | | Other property - INTERVIEWER FIND OUT & WRITE IN | 5 |  |  | |  |
|  |  | | Prefer not to say | 97 |  |  | |  |
|  |  | | Don’t know | 98 |  |  | |  |

| Q135. | | **And how much did it cost you to replace the property?**  SINGLE CODE ONLY | | | | |  | |
| --- | --- | --- | --- | --- | --- | --- | --- | --- |
|  |  | |  |  |  |  | |  |
|  |  | | WRITE IN AMOUNT IN LOCAL CURRENCY AND CODE ‘1’ | 1  ☐☐☐ |  |  | |  |
|  |  | | Prefer not to say | 97 |  |  | |  |
|  |  | | Don’t know | 98 |  |  | |  |

| Q136. | | **Was your husband or partner required to take any time off work as a result of these incidents in the last 12 months?**  SINGLE CODE ONLY | | | | |  | |
| --- | --- | --- | --- | --- | --- | --- | --- | --- |
|  |  | |  |  |  |  | |  |
|  |  | | Yes | 1 | ask q137 |  | |  |
|  |  | | No | 2 | go to q139 |  | |  |
|  |  | | My husband / partner does not work | 3 |  |  | |  |
|  |  | | Prefer not to say | 97 |  |  | |  |
|  |  | | Don’t know | 98 |  |  | |  |

ASK Q137 IF YES AT Q136 (CODE 1). OTHERS GO TO Q139.

| Q137. | | **How many days did your husband or partner miss work as a result of these incidents in the last 12 months?**  SINGLE CODE ONLY | | | | |  | |
| --- | --- | --- | --- | --- | --- | --- | --- | --- |
|  |  | |  |  |  |  | |  |
|  |  | | WRITE IN NUMBER OF DAYS | 1  ☐☐☐ |  |  | |  |
|  |  | | Prefer not to say | 97 |  |  | |  |
|  |  | | Don’t know | 98 |  |  | |  |

| Q138. | | **And of the days your husband or partner missed work because of these incidents during the last 12 months, did he get paid for those days that he missed?** IF YES ASK **Was that for all of the days or some of the days?**  SINGLE CODE ONLY | | | | |  | |
| --- | --- | --- | --- | --- | --- | --- | --- | --- |
|  |  | |  |  |  |  | |  |
|  |  | | Yes – all of these days | 1 |  |  | |  |
|  |  | | Yes – some of these days, how many? CODE ‘2’ AND WRITE IN NUMBER OF DAYS | 2  ☐☐☐ |  |  | |  |
|  |  | | No – not paid for any of the days missed | 3  ☐☐☐ |  |  | |  |
|  |  | | Prefer not to say | 97 |  |  | |  |
|  |  | | Don’t know | 98 |  |  | |  |

ASK Q139 IF THE WOMAN OR GIRL HAS EXPERIENCED ANY VIOLENCE FROM CURRENT OR PREVIOUS HUSBAND OR PARTNER (CODE ‘1’ AT Q105).

| Q139. | | SHOWCARD 47 **Did your husband or partner have to stop doing any of these things as a result of these incidents during the last 12 months?**  SINGLE CODE FOR EACH | | | | | | |  | |
| --- | --- | --- | --- | --- | --- | --- | --- | --- | --- | --- |
|  |  | |  | Yes | No | Don’t know |  |  | |  |
|  | a) | | Fetching water | 1 | 2 | 98 |  |  | |  |
|  | b) | | Fetching wood | 1 | 2 | 98 |  |  | |  |
|  | c) | | Caring for children | 1 | 2 | 98 |  |  | |  |
|  | d) | | Ironing | 1 | 2 | 98 |  |  | |  |
|  | e) | | Washing clothes | 1 | 2 | 98 |  |  | |  |
|  | f) | | Sweeping | 1 | 2 | 98 |  |  | |  |
|  | g) | | Washing dishes | 1 | 2 | 98 |  |  | |  |
|  | h) | | Washing vehicles | 1 | 2 | 98 |  |  | |  |
|  | i) | | Disposing garbage | 1 | 2 | 98 |  |  | |  |
|  | j) | | Cooking | 1 | 2 | 98 |  |  | |  |
|  | k) | | Shopping for household needs | 1 | 2 | 98 |  |  | |  |
|  | l) | | Running errands | 1 | 2 | 98 |  |  | |  |
|  | m) | | Taking care of livestock/poultry | 1 | 2 | 98 |  |  | |  |
|  | n) | | Making clothes for family | 1 | 2 | 98 |  |  | |  |

| Q140. | | SHOWCARD 48 **Thinking about these incidents during the last 12 months. To what extent do you agree or disagree with each of the following statements?**  READ OUT A TO D. SINGLE CODE FOR EACH | | | | | | | |  |
| --- | --- | --- | --- | --- | --- | --- | --- | --- | --- | --- |
|  |  | |  | Strongly agree | Tend to agree | Neither agree nor disagree | Tend to disagree | Strongly disagree | Don’t know | |
|  | a) | | My daily life suffered | 1 | 2 | 3 | 4 | 5 | 98 | |
|  | b) | | I felt unable to play a useful part in life | 1 | 2 | 3 | 4 | 5 | 98 | |
|  | c) | | I found it difficult to enjoy daily activities | 1 | 2 | 3 | 4 | 5 | 98 | |
|  | d) | | I thought about ending my life | 1 | 2 | 3 | 4 | 5 | 98 | |

ASK ALL

**VIOLENCE BY OTHER FAMILY MEMBERS**

**Women and girls also face problems or conflicts with other family members who live with them. I would like to ask about some of your experiences in this regard.**

| Q141. | | **Do you live with other people who are not your husband or partner? This could be with your parents, or parent-in-law, brothers and sisters, and other family members.**  SINGLE CODE ONLY | | | | |  | |
| --- | --- | --- | --- | --- | --- | --- | --- | --- |
|  |  | |  |  |  |  | |  |
|  |  | | Yes | 1 |  |  | |  |
|  |  | | No | 2 |  |  | |  |
|  |  | | Prefer not to say | 97 |  |  | |  |
|  |  | | Don’t know | 98 |  |  | |  |

ASK Q142 IF CODE ‘1’ AT Q141. OTHERS GO TO Q179 IN THE NEXT SECTION.

| Q142 | | SHOWCARD 49 **In the last 12 months, how many times, if any, did a member of your family other than your husband or partner who is living with you do any of the following things?**  READ OUT A TO D. SINGLE CODE FOR EACH | | | | | | | |  | |  | |
| --- | --- | --- | --- | --- | --- | --- | --- | --- | --- | --- | --- | --- | --- |
|  |  |  | Never | Once or twice | Between 3 and 5 times | Between 6 and 10 times | Between 11 and 20 times | More than 20 times | Prefer not to say | | Don’t know | |  |
|  | a) | Insult you or make you feel bad | 1 | 2 | 3 | 4 | 5 | 6 | 97 | | 98 | |  |
|  | b) | Belittle or humiliate you in front of other people | 1 | 2 | 3 | 4 | 5 | 6 | 97 | | 98 | |  |
|  | c) | Do things to scare or frighten you | 1 | 2 | 3 | 4 | 5 | 6 | 97 | | 98 | |  |
|  | d) | Threaten to hurt you or someone you care about | 1 | 2 | 3 | 4 | 5 | 6 | 97 | | 98 | |  |

| Q143. | | | SHOWCARD 50 **And who** <<<aspect from Q142>>?  READ OUT FOR ALL BEHAVIOURS THAT HAPPENED ONCE OR MORE TIME AT Q142. CODE ALL THAT APPLY | | | | | | | |  | |  | |
| --- | --- | --- | --- | --- | --- | --- | --- | --- | --- | --- | --- | --- | --- | --- |
|  |  |  | | Father | Mother | Father-in-law | Mother-in-law | Siblings | Brother or Sister in-Law | Other relative (SPECIFY) | | Prefer not to say | |  |
|  | a) | Insulted you or made you feel bad? | | 1 | 2 | 3 | 4 | 5 | 6 | 7 | | 97 | |  |
|  | b) | Belittled or humiliated you in front of other people? | | 1 | 2 | 3 | 4 | 5 | 6 | 7 | | 97 | |  |
|  | c) | Did things to scare or frighten you? | | 1 | 2 | 3 | 4 | 5 | 6 | 7 | | 97 | |  |
|  | d) | Threatened to hurt you or someone you care about? | | 1 | 2 | 3 | 4 | 5 | 6 | 7 | | 97 | |  |

| Q144. | | SHOWCARD 51 **In the last 12 months,** **how many times, if any, did a member of your family other than your husband or partner who is living with you do the following things?**  READ OUT A TO I. SINGLE CODE FOR EACH | | | | | | |  | | |  | |
| --- | --- | --- | --- | --- | --- | --- | --- | --- | --- | --- | --- | --- | --- |
|  |  |  | Never | Once or twice | Between 3 and 5 times | Between 6 and 10 times | Between 11 and 20 times | More than 20 times | | Prefer not to say | Don’t know | |  |
|  | a) | Slap you or throw something at you which could hurt you | 1 | 2 | 3 | 4 | 5 | 6 | | 97 | 98 | |  |
|  | b) | Push or shove you | 1 | 2 | 3 | 4 | 5 | 6 | | 97 | 98 | |  |
|  | c) | Hit you with a fist or with something else which could hurt you | 1 | 2 | 3 | 4 | 5 | 6 | | 97 | 98 | |  |
|  | d) | Kick, drag, beat, choke or burn you | 1 | 2 | 3 | 4 | 5 | 6 | | 97 | 98 | |  |
|  | e) | Threaten to use or actually use a gun, knife or other weapon against you | 1 | 2 | 3 | 4 | 5 | 6 | | 97 | 98 | |  |
|  | f) | Throw you out of the house | 1 | 2 | 3 | 4 | 5 | 6 | | 97 | 98 | |  |
|  | g) | Physically force you to have sex when you did not want to | 1 | 2 | 3 | 4 | 5 | 6 | | 97 | 98 | |  |
|  | h) | Using threats or intimidation to get you to have sex when you did not want to | 1 | 2 | 3 | 4 | 5 | 6 | | 97 | 98 | |  |
|  | i) | Force you to do something else sexual that you did not want to | 1 | 2 | 3 | 4 | 5 | 6 | | 97 | 98 | |  |

ASK Q145 FOR EACH ASPECT THAT HAS HAPPENDED AT Q144 (CODES 2 TO 6). IF NEVER IS CODED AT ALL OF QUESTIONS Q144 A-I – THEN GO TO Q179 IN THE NEXT SECTION.

| Q145. | | | SHOWCARD 52 **And who** <<<aspect from Q144>>?  CODE ALL THAT APPLY | | | | | | | |  | |  | |
| --- | --- | --- | --- | --- | --- | --- | --- | --- | --- | --- | --- | --- | --- | --- |
|  |  |  | | Father | Mother | Father-in-law | Mother-in-law | Siblings | Brother or Sister in-Law | Other relative (SPECIFY) | | Prefer not to say | |  |
|  | a) | Slapped you or threw something at you which could hurt you | | 1 | 2 | 3 | 4 | 5 | 6 | 7 | | 97 | |  |
|  | b) | Pushed or shoved you | | 1 | 2 | 3 | 4 | 5 | 6 | 7 | | 97 | |  |
|  | c) | Hit you with a fist or with something else which could hurt you | | 1 | 2 | 3 | 4 | 5 | 6 | 7 | | 97 | |  |
|  | d) | Kicked, dragged, beat, choked or burnt you | | 1 | 2 | 3 | 4 | 5 | 6 | 7 | | 97 | |  |
|  | e) | Threatened to use or actually used a gun, knife or other weapon against you | | 1 | 2 | 3 | 4 | 5 | 6 | 7 | | 97 | |  |
|  | f) | Threw you out of the house | | 1 | 2 | 3 | 4 | 5 | 6 | 7 | | 97 | |  |
|  | g) | Physically forced you to have sex when you did not want to | | 1 | 2 | 3 | 4 | 5 | 6 | 7 | | 97 | |  |
|  | h) | Using threats or intimidation to get you to have sex when you did not want to | | 1 | 2 | 3 | 4 | 5 | 6 | 7 | | 97 | |  |
|  | i) | Forced you to do something else sexual that you did not want to | | 1 | 2 | 3 | 4 | 5 | 6 | 7 | | 97 | |  |

| Q146. | | **Thinking about the different behaviours you have reported that your family members have done in the last 12 months, how many incidents of this nature do you remember? This maybe a single behaviour or combination of behaviours as reported above** SINGLE CODE ONLY | | | | |
| --- | --- | --- | --- | --- | --- | --- |
|  |  | |  |  |  |  |
|  |  | | CODE ‘1’ AND WRITE IN THE NUMBER OF INCIDENTS | 1  ☐☐ |  |  |
|  |  | | None | 2 |  |  |
|  |  | | Prefer not to say | 97 |  |  |
|  |  | | Don’t know | 98 |  |  |

| Q147. | | SHOWCARD 53 **Which, if any, of these things did you experience as a result of all incidents from members of your family living with you in the last 12 months? Read out those that apply.**  MULTICODE OKAY. | | | | | | |  | |
| --- | --- | --- | --- | --- | --- | --- | --- | --- | --- | --- |
|  |  | |  |  |  |  |  |  | |  |
|  |  | | Puncture wounds or bite marks | 1 |  |  |  |  | |  |
|  |  | | Scratches or bruises | 2 |  |  |  |  | |  |
|  |  | | Sprains or dislocations | 3 |  |  |  |  | |  |
|  |  | | Burns | 4 |  |  |  |  | |  |
|  |  | | Cuts, gashes or bleeding | 5 |  |  |  |  | |  |
|  |  | | Eye injury | 6 |  |  |  |  | |  |
|  |  | | Fractures of broken bones | 7 |  |  |  |  | |  |
|  |  | | Broken eardrum | 8 |  |  |  |  | |  |
|  |  | | Other injury - INTERVIEWER FIND OUT & WRITE IN | 9 |  |  |  |  | |  |
|  |  | | None of these | 10 |  |  |  |  | |  |
|  |  | | Prefer not to say | 97 |  |  |  |  | |  |
|  |  | | Don’t know | 98 |  |  |  |  | |  |

| Q148. | | **Did you require any healthcare (including medical treatment or counselling) as a result of these incidents by members of your family who are living with you in the last 12 months?**  SINGLE CODE ONLY | | | | |  | |
| --- | --- | --- | --- | --- | --- | --- | --- | --- |
|  |  | |  |  |  |  | |  |
|  |  | | Yes | 1 | ask q149 |  | |  |
|  |  | | No | 2 | go to q151 |  | |  |
|  |  | | Prefer not to say | 97 |  |  | |  |
|  |  | | Don’t know | 98 |  |  | |  |

ASK Q149 AND Q150 IF YES (CODE ‘1’ AT Q148). OTHERS GO TO Q151

| Q149. | | **How much, if anything, money did you have to spend on healthcare as a result of these incidents? (doctors’ fees, medicines, lab tests)?**  SINGLE CODE ONLY | | | | |  | |
| --- | --- | --- | --- | --- | --- | --- | --- | --- |
|  |  | |  |  |  |  | |  |
|  |  | | CODE ‘1’ AND WRITE IN THE AMOUNT IN LOCAL CURRENCY | 1  ☐☐☐☐ |  |  | |  |
|  |  | | None | 2 |  |  | |  |
|  |  | | Prefer not to say | 97 |  |  | |  |
|  |  | | Don’t know | 98 |  |  | |  |

| Q150. | | **And how much, if anything, did you have to spend on transport to and from the hospital or health clinic as a result of injuries caused by members of your family who are living with you in the last 12 months?**  SINGLE CODE ONLY | | | | |  | |
| --- | --- | --- | --- | --- | --- | --- | --- | --- |
|  |  | |  |  |  |  | |  |
|  |  | | CODE ‘1’ AND WRITE IN AMOUNT IN LCOAL CURRENCY | 1  ☐☐☐☐ |  |  | |  |
|  |  | | None | 2 |  |  | |  |
|  |  | | Prefer not to say | 97 |  |  | |  |
|  |  | | Don’t know | 98 |  |  | |  |

ASK Q151 IF THE WOMAN OR GIRL HAS EXPERIENCED ANY VIOLENCE FROM A FAMILY MEMBER (CODE ‘1’’ AT Q146) OTHERS GO TO Q179 IN THE NEXT SECTION.

| Q151. | | **Please remind me - do you earn an income from any work or employment?**  SINGLE CODE ONLY | | | | |  | |
| --- | --- | --- | --- | --- | --- | --- | --- | --- |
|  |  | |  |  |  |  | |  |
|  |  | | Yes | 1 | ask q152 |  | |  |
|  |  | | No | 2 | go to q155 |  | |  |
|  |  | | Prefer not to say | 3 |  |  | |  |

ASK Q152 TO Q154 IF YES (CODE ‘1’) AT Q151. OTHERS GO TO Q155

| Q152. | | **Were you required to take any time off work as a result of these incidents by members of your family who are living with you in the last 12 months?**  SINGLE CODE ONLY | | | | |  | |
| --- | --- | --- | --- | --- | --- | --- | --- | --- |
|  |  | |  |  |  |  | |  |
|  |  | | Yes | 1 |  |  | |  |
|  |  | | No | 2 |  |  | |  |
|  |  | | Prefer not to say | 97 |  |  | |  |
|  |  | | Don’t know | 98 |  |  | |  |

| Q153. | | **How many days did you miss work as a result of these incidents by members of your family who are living with you in the last 12 months?**  SINGLE CODE ONLY | | | | |  | |
| --- | --- | --- | --- | --- | --- | --- | --- | --- |
|  |  | |  |  |  |  | |  |
|  |  | | CODE ‘1’ AND WRITE IN THE NUMBER OF DAYS | 1  ☐☐☐ |  |  | |  |
|  |  | | Prefer not to say | 97 |  |  | |  |
|  |  | | Don’t know | 98 |  |  | |  |

| Q154. | | **And of the days you missed work because these incidents by members of your family who are living with you in the last 12 months, did you get paid for the days you missed?** IF YES ASK **Was that for all of the days or some of the days?**  SINGLE CODE ONLY | | | | |  | |
| --- | --- | --- | --- | --- | --- | --- | --- | --- |
|  |  | |  |  |  |  | |  |
|  |  | | Yes – all of these days | 1 |  |  | |  |
|  |  | | Yes – some of these days, how many? CODE ‘2’ AND WRITE IN NUMBER OF DAYS | 2  ☐☐☐ |  |  | |  |
|  |  | | No – not paid for any of the days missed | 3 |  |  | |  |
|  |  | | Prefer not to say | 97 |  |  | |  |
|  |  | | Don’t know | 98 |  |  | |  |

ASK Q155 THE WOMAN OR GIRL HAS EXPERIENCED ANY VIOLENCE FROM A FAMILY MEMBER (CODE ‘1’’ AT Q146) OTHERS GO TO Q179 IN THE NEXT SECTION.

.

| Q155. | | **Did you fully or partially stop any of the following tasks as a result of these incidents by members of your family who are living with you in the last 12 months?**  READ OUT A TO N. SINGLE CODE FOR EACH  IF CODE 1 OR CODE 2 USED, ASK NUMBER OF DAYS AND WRITE IN. | | | | | | |  | |
| --- | --- | --- | --- | --- | --- | --- | --- | --- | --- | --- |
|  |  | |  | Fully Stopped | Days | Partially stopped (50%) | Days | No Effect | |  |
|  | a) | | Fetching water | 1 | ☐☐☐ | 2 | ☐☐☐ | 99 | |  |
|  | b) | | Fetching wood | 1 | ☐☐☐ | 2 | ☐☐☐ | 99 | |  |
|  | c) | | Caring for children | 1 | ☐☐☐ | 2 | ☐☐☐ | 99 | |  |
|  | d) | | Ironing | 1 | ☐☐☐ | 2 | ☐☐☐ | 99 | |  |
|  | e) | | Washing clothes | 1 | ☐☐☐ | 2 | ☐☐☐ | 99 | |  |
|  | f) | | Sweeping | 1 | ☐☐☐ | 2 | ☐☐☐ | 99 | |  |
|  | g) | | Washing dishes | 1 | ☐☐☐ | 2 | ☐☐☐ | 99 | |  |
|  | h) | | Washing vehicles | 1 | ☐☐☐ | 2 | ☐☐☐ | 99 | |  |
|  | i) | | Disposing garbage | 1 | ☐☐☐ | 2 | ☐☐☐ | 99 | |  |
|  | j) | | Cooking | 1 | ☐☐☐ | 2 | ☐☐☐ | 99 | |  |
|  | k) | | Shopping for household needs | 1 | ☐☐☐ | 2 | ☐☐☐ | 99 | |  |
|  | l) | | Running errands | 1 | ☐☐☐ | 2 | ☐☐☐ | 99 | |  |
|  | m) | | Taking care of livestock or poultry | 1 | ☐☐☐ | 2 | ☐☐☐ | 99 | |  |
|  | n) | | Making clothes for family | 1 | ☐☐☐ | 2 | ☐☐☐ | 99 | |  |

ASK Q156 AND Q157 IF YES (CODE ‘1’ OR CODE ‘2’ ) AT Q155C. OTHERS GO TO Q158

| Q156. | | **How many days did you have to stop caring for children as a result of these incidents by members of your family who are living with you in the last 12 months?**  SINGLE CODE ONLY | | | | |  | |
| --- | --- | --- | --- | --- | --- | --- | --- | --- |
|  |  | |  |  |  |  | |  |
|  |  | | CODE ‘1’ AND WRITE IN THE NUMBER OF DAYS | 1  ☐☐☐ |  |  | |  |
|  |  | | Prefer not to say | 97 |  |  | |  |
|  |  | | Don’t know | 98 |  |  | |  |

| Q157. | | SHOWCARD 54 **And thinking about when you had to stop caring for children because of these incidents caused by members of your family who are living with you, how were your children fed?** SINGLE CODE ONLY | | | | |  | |
| --- | --- | --- | --- | --- | --- | --- | --- | --- |
|  |  | |  |  |  |  | |  |
|  | A | | Children fed themselves | 1 |  |  | |  |
|  | B | | Children were fed by someone else | 2 |  |  | |  |
|  | C | | Children went hungry | 3 |  |  | |  |
|  |  | | Prefer not to say | 97 |  |  | |  |
|  |  | | Don’t know | 98 |  |  | |  |

ASK Q158 AND Q159 IF THE WOMAN OR GIRL HAS SCHOOL AGED CHILDREN (CODE 1 AT Q95). OTHERS GO TO Q160.

| Q158. | | SHOWCARD 55 **Which, if any, of these things happened to your children as a result of these incidents by members of your family living with you in the last 12 months?**  MULTICODE OKAY | | | | |  | |
| --- | --- | --- | --- | --- | --- | --- | --- | --- |
|  |  | |  |  |  |  | |  |
|  | A | | Felt scared | 1 |  |  | |  |
|  | B | | Felt confused | 2 |  |  | |  |
|  | C | | Asked lots of questions | 3 |  |  | |  |
|  | D | | Wet the bed | 4 |  |  | |  |
|  | E | | Had nightmares | 5 |  |  | |  |
|  | F | | Physically shook | 6 |  |  | |  |
|  | G | | Did not want to play | 7 |  |  | |  |
|  | H | | None of these | 8 |  |  | |  |
|  |  | | Prefer not to say | 97 |  |  | |  |
|  |  | | Don’t know | 98 |  |  | |  |

| Q159. | | **How many days did your children miss school as a result of these incidents in the last 12 months?** SINGLE CODE ONLY | | | | |
| --- | --- | --- | --- | --- | --- | --- |
|  |  | |  |  |  |  |
|  |  | | CODE ‘1’ AND WRITE IN THE NUMBER OF DAYS | 1  ☐☐☐ |  |  |
|  |  | | Prefer not to say | 97 |  |  |
|  |  | | Don’t know | 98 |  |  |

| Q160. | | **Did you report any of these incidents to the police?**  SINGLE CODE ONLY | | | | |  | |
| --- | --- | --- | --- | --- | --- | --- | --- | --- |
|  |  | |  |  |  |  | |  |
|  |  | | Yes | 1 | ask q161 |  | |  |
|  |  | | No | 2 | go to q170 |  | |  |
|  |  | | Prefer not to say | 97 |  |  | |  |
|  |  | | Don’t know | 98 |  |  | |  |

ASK Q161 TO Q170 IF CODE 1 AT Q160. OTHERS GO TO Q171.

| Q161. | | **Did it cost any money to file a complaint?**  SINGLE CODE ONLY | | | | |
| --- | --- | --- | --- | --- | --- | --- |
|  |  | |  |  |  |  |
|  |  | | Yes | 1 | ask q162 |  |
|  |  | | No | 2 | go to q163 |  |
|  |  | | Prefer not to say | 97 |  |  |
|  |  | | Don’t know | 98 |  |  |

ASK Q162 IF YES (CODE ‘1’) AT Q161

| Q162. | | **And how much did it cost you to file a complaint?**  SINGLE CODE ONLY | | | | |
| --- | --- | --- | --- | --- | --- | --- |
|  |  | |  |  |  |  |
|  |  | | CODE ‘1’ AND WRITE IN THE AMOUNT IN LOCAL CURRENCY | 1  ☐☐☐☐ |  |  |
|  |  | | Prefer not to say | 97 |  |  |
|  |  | | Don’t know | 98 |  |  |

| Q163. | | **Did you have to pay the police any money apart from a filing fee?**  SINGLE CODE ONLY | | | | |  | |
| --- | --- | --- | --- | --- | --- | --- | --- | --- |
|  |  | |  |  |  |  | |  |
|  |  | | Yes | 1 | ASK Q164 |  | |  |
|  |  | | No | 2 | GO TO Q165 |  | |  |
|  |  | | Prefer not to say | 97 |  |  | |  |
|  |  | | Don’t know | 98 |  |  | |  |

ASK Q164 IF CODE 1 AT Q163, OTHERS GO TO Q165.

| Q164. | | **And how much did you have to pay the police?**  SINGLE CODE ONLY | | | | |  | |
| --- | --- | --- | --- | --- | --- | --- | --- | --- |
|  |  | |  |  |  |  | |  |
|  |  | | CODE ‘1’ AND WRITE IN AMOUNT IN LOCAL CURRENCY | 1  ☐☐☐☐ |  |  | |  |
|  |  | | Prefer not to say | 97 |  |  | |  |
|  |  | | Don’t know | 98 |  |  | |  |

| Q165. | | **Did you incur any transport costs going to the police?**  SINGLE CODE ONLY | | | | |
| --- | --- | --- | --- | --- | --- | --- |
|  |  | |  |  |  |  |
|  |  | | Yes | 1 | ASK Q166 |  |
|  |  | | No | 2 | GO TO Q167 |  |
|  |  | | Prefer not to say | 97 |  |  |
|  |  | | Don’t know | 98 |  |  |

ASK Q166 IF YES (CODE ‘1’) AT Q165. OTHERS GO TO Q167.

| Q166. | | **And how much did you spend on transport going to the police?**  SINGLE CODE ONLY | | | | |
| --- | --- | --- | --- | --- | --- | --- |
|  |  | |  |  |  |  |
|  |  | | CODE ‘1’ AND WRITE IN AMOUNT IN LOCAL CURRENCY | 1  ☐☐☐ |  |  |
|  |  | | Prefer not to say | 97 |  |  |
|  |  | | Don’t know | 98 |  |  |

| Q167. | | **Did the complaint go to court?**  SINGLE CODE ONLY | | | | |  | |
| --- | --- | --- | --- | --- | --- | --- | --- | --- |
|  |  | |  |  |  |  | |  |
|  |  | | Yes | 1 |  |  | |  |
|  |  | | No | 2 |  |  | |  |
|  |  | | Prefer not to say | 97 |  |  | |  |
|  |  | | Don’t know | 98 |  |  | |  |

ASK Q168 IF YES (CODE ‘1’ AT Q167). OTHERS GO TO Q170

| Q168. | | **Did it cost you any money to go to court such as lawyers’ fees, and other filing costs?**  SINGLE CODE ONLY | | | | |  | |
| --- | --- | --- | --- | --- | --- | --- | --- | --- |
|  |  | |  |  |  |  | |  |
|  |  | | Yes | 1 | ASK Q169 |  | |  |
|  |  | | No | 2 | GO TO Q170 |  | |  |
|  |  | | Prefer not to say | 97 |  |  | |  |
|  |  | | Don’t know | 98 |  |  | |  |

ASK Q169 IF YES (CODE ‘1’) AT Q168. OTHERS GO TO Q170.

| Q169. | | **And how much did you spend on transport going to court?**  SINGLE CODE ONLY | | | | |  | |
| --- | --- | --- | --- | --- | --- | --- | --- | --- |
|  |  | |  |  |  |  | |  |
|  |  | | CODE ‘1’ AND WRITE IN THE AMOUNT IN LOCAL CURRENCY | 1  ☐☐☐☐ |  |  | |  |
|  |  | | Prefer not to say | 97 |  |  | |  |
|  |  | | Don’t know | 98 |  |  | |  |

| Q170. | | **Thinking about the incidents in the last 12 months by members of your family who are living with you, did you leave home and seek shelter somewhere else?**  SINGLE CODE ONLY | | | | |  | |
| --- | --- | --- | --- | --- | --- | --- | --- | --- |
|  |  | |  |  |  |  | |  |
|  |  | | Yes | 1 |  |  | |  |
|  |  | | No | 2 |  |  | |  |
|  |  | | Prefer not to say | 97 |  |  | |  |
|  |  | | Don’t know | 98 |  |  | |  |

ASK Q171 TO Q173 IF YES (CODE ‘1’) AT Q170. OTHERS GO TO Q174.

| Q171. | | **Where did you go?**  MULTICODE OKAY | | | | |  | |
| --- | --- | --- | --- | --- | --- | --- | --- | --- |
|  |  | |  |  |  |  | |  |
|  |  | | Stayed with other relatives | 1 |  |  | |  |
|  |  | | Stayed with friends | 2 |  |  | |  |
|  |  | | Stayed in a shelter | 3 |  |  | |  |
|  |  | | Stayed somewhere else - INTERVIEWER FIND OUT & WRITE IN) | 4 |  |  | |  |
|  |  | | Prefer not to say | 97 |  |  | |  |
|  |  | | Don’t know | 98 |  |  | |  |

| Q172. | | **How many days did you stay away?**  MULTICODE OKAY | | | | |  | |
| --- | --- | --- | --- | --- | --- | --- | --- | --- |
|  |  | |  |  |  |  | |  |
|  |  | | CODE ‘1’ AND WRITE IN THE NUMBER OF DAYS | 1  ☐☐☐ |  |  | |  |
|  |  | | I am still away | 2 |  |  | |  |
|  |  | | Prefer not to say | 97 |  |  | |  |
|  |  | | Don’t know | 98 |  |  | |  |

| Q173. | | **Did you have to pay any money to stay away – for example, pay for accommodation, food, etc.?**  SINGLE CODE ONLY | | | | |  | |
| --- | --- | --- | --- | --- | --- | --- | --- | --- |
|  |  | |  |  |  |  | |  |
|  |  | | Yes CODE ‘1’ AND WRITE AMOUNT IN LOCAL CURRENCY | 1  ☐☐☐ |  |  | |  |
|  |  | | No | 2 |  |  | |  |
|  |  | | Prefer not to say | 97 |  |  | |  |
|  |  | | Don’t know | 98 |  |  | |  |

| Q174. | | **Did you have to replace any property as a consequence of these incidents by family members who are living with you, apart from your husband or partner?**  SINGLE CODE ONLY | | | | |  | |
| --- | --- | --- | --- | --- | --- | --- | --- | --- |
|  |  | |  |  |  |  | |  |
|  |  | | Yes | 1 |  |  | |  |
|  |  | | No | 2 |  |  | |  |
|  |  | | Prefer not to say | 97 |  |  | |  |
|  |  | | Don’t know | 98 |  |  | |  |

ASK Q175 AND Q176 IF YES (CODE ‘1’) AT Q174. OTHERS GO TO Q177.

| Q175. | | **What kind of property was it?**  MULTICODE OKAY | | | | |  | |
| --- | --- | --- | --- | --- | --- | --- | --- | --- |
|  |  | |  |  |  |  | |  |
|  |  | | Dishes, pots or utensils | 1 |  |  | |  |
|  |  | | Furniture | 2 |  |  | |  |
|  |  | | Television or radio | 3 |  |  | |  |
|  |  | | Repairs to the house | 4 |  |  | |  |
|  |  | | Other property - INTERVIEWER FIND OUT & WRITE IN | 5 |  |  | |  |
|  |  | | Prefer not to say | 97 |  |  | |  |
|  |  | | Don’t know | 98 |  |  | |  |

| Q176. | | **And how much did it cost you to replace the property?**  SINGLE CODE ONLY | | | | |  | |
| --- | --- | --- | --- | --- | --- | --- | --- | --- |
|  |  | |  |  |  |  | |  |
|  |  | | CODE ‘1’ AND WRITE IN THE AMOUNT IN LOCAL CURRENCY | 1  ☐☐☐ |  |  | |  |
|  |  | | Prefer not to say | 97 |  |  | |  |
|  |  | | Don’t know | 98 |  |  | |  |

| Q177. | | SHOWCARD 56 **Thinking about all of the financial costs we discussed as a result of these incidents you have experienced in the last 12 months, who paid these costs?**  MULTICODE OKAY | | | | |  | |
| --- | --- | --- | --- | --- | --- | --- | --- | --- |
|  |  | |  |  |  |  | |  |
|  |  | | I paid the money out of my own money | 1 |  |  | |  |
|  |  | | My partner paid the money out of his own money | 2 |  |  | |  |
|  |  | | My partner and I jointly paid the costs out of household money | 3 |  |  | |  |
|  |  | | Her birth family | 4 |  |  | |  |
|  |  | | His birth family | 5 |  |  | |  |
|  |  | | Someone else paid the financial costs - INTERVIEWER FIND OUT & WRITE IN | 6 |  |  | |  |
|  |  | | Prefer not to say | 97 |  |  | |  |
|  |  | | Don’t know | 98 |  |  | |  |

| Q178. | | SHOWCARD 57 **Thinking about the incidents by your family who are living with you in the last 12 months, to what extent do you agree or disagree with each of the following statements…**  READ OUT A TO D. SINGLE CODE FOR EACH | | | | | | | |  |
| --- | --- | --- | --- | --- | --- | --- | --- | --- | --- | --- |
|  |  | |  | Strongly agree | Tend to agree | Neither agree nor disagree | Tend to disagree | Strongly disagree | Don’t know | |
|  | a) | | My daily life suffered | 1 | 2 | 3 | 4 | 5 | 98 | |
|  | b) | | I felt unable to play a useful part in life | 1 | 2 | 3 | 4 | 5 | 98 | |
|  | c) | | I found it difficult to enjoy daily activities | 1 | 2 | 3 | 4 | 5 | 98 | |
|  | d) | | I thought about ending my life | 1 | 2 | 3 | 4 | 5 | 98 | |

| VIOLENCE AT THE WORKPLACE |
| --- |

ASK IF WORKING IN PAID EMPLOYMENT (CODE 1 AT Q57A). OTHERS GO TO Q206 IN THE NEXT SECTION.

**Women and girls can encounter difficult situations in their place of work. We know that sometimes colleagues, people in authority and peers can behave inappropriately. I would now like to ask you some questions about your experiences at work. These are important to document so that we can develop stronger laws and appropriate services for women in the workplace**.

| Q179. | | SHOWCARD 58 **To what extent, do you agree or disagree with each of the following statements about your workplace?**  READ OUT A TO D. SINGLE CODE FOR EACH | | | | | | | |  |
| --- | --- | --- | --- | --- | --- | --- | --- | --- | --- | --- |
|  |  | |  | Strongly agree | Tend to agree | Neither agree nor disagree | Tend to disagree | Strongly disagree | Don’t know | |
|  | a) | | I am treated as an equal | 1 | 2 | 3 | 4 | 5 | 98 | |
|  | b) | | I am paid less than male colleagues for doing the same job | 1 | 2 | 3 | 4 | 5 | 98 | |
|  | c) | | Female staff have the same access to training opportunities as their male colleagues at my workplace | 1 | 2 | 3 | 4 | 5 | 98 | |
|  | d) | | Women are more likely than men to be overlooked for promotion at my workplace | 1 | 2 | 3 | 4 | 5 | 98 | |

| Q180. | | SHOWCARD 59 **Which, if any, of these behaviours have you experienced in your workplace in the last 12 months? Read out the category or categories from this card that apply.**  SINGLE CODE FOR EACH | | | | | | | |  | |
| --- | --- | --- | --- | --- | --- | --- | --- | --- | --- | --- | --- |
|  |  | |  | Yes | No | Prefer  not to say |  |  |  | |  |
|  | A | | **Verbal Violence:**  Someone verbally intimidated, humiliated or insulted you? | 1 | 2 | 97 |  |  |  | |  |
|  | B | | **Physical violence:**  Someone slapped, pushed, punched, kicked, tried to burn you, pointed a gun, knife or any other weapon at you? | 1 | 2 | 97 |  |  |  | |  |
|  | C | | **Sexual Harassment:**  Someone verbally harassed you in a sexual manner? Leered at you? Made sexual jokes? Belittling/humiliating sexual comments? | 1 | 2 | 97 |  |  |  | |  |
|  | D | | **Sexual Assault:**  Someone grabbed, groped or otherwise touched you sexually without your consent | 1 | 2 | 97 |  |  |  | |  |
|  | E | | **Sexual Assault**:  Someone forced you to touch them or forced you (made you drunk, drugged you, threatened you so you could not refuse, physically forced you) to engage in sexual acts without your consent? | 1 | 2 | 97 |  |  |  | |  |

ASK Q181 AND Q182 FOR EACH ASPECT (A TO E) CODED 1 (YES) AT Q180. IF ALL ASPECTS (A-E) CODED 2 [NO] GO TO Q206.

| Q181. | | SHOWCARD 60 **And how often in the last 12 months did you experience…**  READ OUT A TO E IF YES AT Q180. SINGLE CODE FOR EACH | | | | | | |  | |
| --- | --- | --- | --- | --- | --- | --- | --- | --- | --- | --- |
|  |  | |  | Once or twice | Between 3 and 5 times | Between 6 and 10 times | Between 11 and 20 times | More than 20 times | | Don’t know |
|  | a) | | …verbal violence in your workplace (code a at Q180)? | 1 | 2 | 3 | 4 | 5 | | 98 |
|  | b) | | …physical violence in your workplace (code b at Q180)? | 1 | 2 | 3 | 4 | 5 | | 98 |
|  | c) | | sexual harassment in your workplace(code c at Q180)? | 1 | 2 | 3 | 4 | 5 | | 98 |
|  | d) | | …sexual assault in your workplace (code d at Q180)? | 1 | 2 | 3 | 4 | 5 | | 98 |
|  | e) | | …sexual assault in your workplace (code e at Q180)? | 1 | 2 | 3 | 4 | 5 | | 98 |

| Q182. | |  | | SHOWCARD 61 **And thinking about the most recent incident of violence in your workplace. When did this occur?**  READ OUT A TO E IF ANSWERED AT Q181. SINGLE CODE FOR EACH. | | | | | | | |  |
| --- | --- | --- | --- | --- | --- | --- | --- | --- | --- | --- | --- | --- |
|  |  | |  | | Within the last week | Within the last month | Between 1 and 3 months ago | Between 3 and 6 months ago | In the last 12 months | Longer than 12 months ago | Don’t  know | |
|  | a) | | Verbal violence | | 1 | 2 | 3 | 4 | 5 | 6 | 98 | |
|  | b) | | Physical violence | | 1 | 2 | 3 | 4 | 5 | 6 | 98 | |
|  | c) | | Sexual harassment | | 1 | 2 | 3 | 4 | 5 | 6 | 98 | |
|  | d) | | Sexual assault | | 1 | 2 | 3 | 4 | 5 | 6 | 98 | |
|  | e) | | Sexual assault | | 1 | 2 | 3 | 4 | 5 | 6 | 98 | |

ASK Q183 TO Q185 IF WOMAN OR GIRL HAS EXPERIENCED SEXUAL HARRASSMENT OR ASSAULT IN THE WORKPLACE (ASPECTS C, D OR E CODED ‘1’ [YES] AT Q180). OTHERS GO TO Q191.

| Q183 | | SHOWCARD 62 **Thinking about the last time you experienced being tricked or forced into sexual behaviours against your will in your workplace, which, if any, of the things on the card happened? Read out the statement or statements that apply.**  MULTICODE OKAY | | | | |  |  |
| --- | --- | --- | --- | --- | --- | --- | --- | --- |
|  |  | |  |  |  |  | | |
|  |  | | Had derogatory nasty / humiliating/belittling sexual comments made about me, received repeated unwanted sexual advances or was threatened with sexual violence | 1 |  |  | | |
|  |  | | Touched me inappropriately without my permission | 2 |  |  | | |
|  |  | | Physically forced me to have sex against my will | 3 |  |  | | |
|  |  | | Forced me to do something sexual that I found to be humiliating or degrading | 4 |  |  | | |
|  |  | | Had sex because of being afraid of what might happen if I did not consent | 5 |  |  | | |
|  |  | | Had sex because I was too drunk or drugged to refuse | 6 |  |  | | |
|  |  | | Other - INTERVIEWER FIND OUT & WRITE IN | 7 |  |  | | |
|  |  | | None of these | 8 |  |  | | |
|  |  | | Prefer not to say | 97 |  |  | | |
|  |  | | Don’t know | 98 |  |  | | |

| Q184 | | SHOWCARD 63 **Thinking about the last time you experienced being tricked or forced into sexual behaviours against your will in your workplace, who did this to you? Read out the category or categories that apply.**  MULTICODE OKAY | | | | |  | |
| --- | --- | --- | --- | --- | --- | --- | --- | --- |
|  |  | |  |  |  |  | |  |
|  |  | | A supervisor or manager | 1 |  |  | |  |
|  |  | | A work colleague at the same level as me | 2 |  |  | |  |
|  |  | | A work colleague in a less senior level than me | 3 |  |  | |  |
|  |  | | A client or customer | 4 |  |  | |  |
|  |  | | Someone else did this to me - INTERVIEWER FIND OUT & WRITE IN | 5 |  |  | |  |
|  |  | | None of these | 6 |  |  | |  |
|  |  | | Prefer not to say | 97 |  |  | |  |
|  |  | | Don’t know | 98 |  |  | |  |

| Q185. | | SHOWCARD 64 **Thinking about the last time you experienced being tricked or forced into sexual behaviours against your will in your workplace, which, of any of the things on the card did you do about it? Read out the statement or statements that apply.**  MULTICODE OKAY | | | | |  | |
| --- | --- | --- | --- | --- | --- | --- | --- | --- |
|  |  | |  |  |  |  | |  |
|  |  | | I requested help from my family | 1 | GO TO Q187 |  | |  |
|  |  | | I requested help from my friends | 2 |  |  | |  |
|  |  | | Reported to my boss/supervisor/manager | 3 |  |  | |  |
|  |  | | Reported the incident to the police | 4 |  |  | |  |
|  |  | | I went to a hospital or health clinic | 5 |  |  | |  |
|  |  | | I went to a support organisation | 6 |  |  | |  |
|  |  | | Visited a mosque/religious person | 7 |  |  | |  |
|  |  | | Other - INTERVIEWER FIND OUT & WRITE IN | 8 |  |  | |  |
|  |  | | I did not seek any help | 9 | ASK Q186 |  | |  |
|  |  | | Prefer not to say | 97 | GO TO Q187 |  | |  |
|  |  | | Don’t know | 98 |  |  | |  |

ASK Q186 IF THE WOMAN OR GIRL DID NOT SEEK HELP ON THE LAST TIME SHE EXPERIENCED SEXUAL VIOLENCE IN THE WORKPLACE (CODE 9 AT Q185). OTHERS GO TO Q187.

| Q186. | | SHOWCARD 65 **Why did you not seek help on the last time that you experienced being tricked or forced into sexual behaviours against your will in the workplace? Read out the statement or statements that apply.**  MULTICODE OK | | | | |  | |
| --- | --- | --- | --- | --- | --- | --- | --- | --- |
|  |  | |  |  |  |  | |  |
|  |  | | It was my fault | 1 |  |  | |  |
|  |  | | Shame | 2 |  |  | |  |
|  |  | | Fear of being excluded or shunned by colleagues | 3 |  |  | |  |
|  |  | | To maintain family honour | 4 |  |  | |  |
|  |  | | Fear that husband or partner would make me stop work | 5 |  |  | |  |
|  |  | | Fear of violence from husband or partner | 6 |  |  | |  |
|  |  | | It would negatively affect my chances of promotion | 7 |  |  | |  |
|  |  | | I did not think anyone would believe me | 8 |  |  | |  |
|  |  | | I did not see the point | 9 |  |  | |  |
|  |  | | Other reason - INTERVIEWER FIND OUT & WRITE IN | 10 |  |  | |  |
|  |  | | None of these | 11 |  |  | |  |
|  |  | | Prefer not to say | 97 |  |  | |  |
|  |  | | Don’t know | 98 |  |  | |  |

ASK Q187 IF THE WOMAN OR GIRL EXPERIENCED PHYSICAL VIOLENCE IN THE WORKPLACE (ASPECT B CODED ‘1’ [YES] AT Q180. OTHERS GO TO Q191.

| Q187. | | SHOWCARD 66 **Thinking about the last time you experienced physical violence in your workplace, which, if any, of these things happened? Read out the statement or statements that apply.**  MULTICODE OKAY | | | | |  | |
| --- | --- | --- | --- | --- | --- | --- | --- | --- |
|  |  | |  |  |  |  | |  |
|  |  | | I was threatened that I or someone I care about would be physically hurt | 1 |  |  | |  |
|  |  | | I was slapped | 2 |  |  | |  |
|  |  | | I had something thrown at me | 3 |  |  | |  |
|  |  | | I was pushed or shoved | 4 |  |  | |  |
|  |  | | I was hit with a fist | 5 |  |  | |  |
|  |  | | I was kicked, dragged or beaten up | 6 |  |  | |  |
|  |  | | I was choked or burned on purpose | 7 |  |  | |  |
|  |  | | I was threatened with a weapon such as a stick or knife | 8 |  |  | |  |
|  |  | | Something else happened - INTERVIEWER FIND OUT & WRITE IN) | 9 |  |  | |  |
|  |  | | None of these | 10 |  |  | |  |
|  |  | | Prefer not to say | 97 |  |  | |  |
|  |  | | Don’t know | 98 |  |  | |  |

| Q188. | | SHOWCARD 67 **Thinking about the last time you experienced physical violence in your workplace, who did this to you? Read out the category or categories on the card that apply.**  MULTICODE OKAY | | | | |  | |
| --- | --- | --- | --- | --- | --- | --- | --- | --- |
|  |  | |  |  |  |  | |  |
|  |  | | A supervisor or manager | 1 |  |  | |  |
|  |  | | A work colleague at the same level as me | 2 |  |  | |  |
|  |  | | A work colleague in a less senior level than me | 3 |  |  | |  |
|  |  | | A client or customer | 4 |  |  | |  |
|  |  | | Someone else did this to me - INTERVIEWER FIND OUT & WRITE IN | 5 |  |  | |  |
|  |  | | None of these | 6 |  |  | |  |
|  |  | | Prefer not to say | 97 |  |  | |  |
|  |  | | Don’t know | 98 |  |  | |  |

| Q189. | | SHOWCARD 68 **Thinking about the last time you experienced physical violence in your workplace, which, of any of these things did you do about it? Read out the statement or statements that apply.**  MULTICODE OKAY | | | | |  | |
| --- | --- | --- | --- | --- | --- | --- | --- | --- |
|  |  | |  |  |  |  | |  |
|  |  | | I requested help from my family | 1 | GO TO Q191 |  | |  |
|  |  | | I requested help from my friends | 2 |  |  | |  |
|  |  | | Reported to my boss/supervisor/manager | 3 |  |  | |  |
|  |  | | Reported the incident to the police | 4 |  |  | |  |
|  |  | | I went to a hospital or health clinic | 5 |  |  | |  |
|  |  | | I went to a support organisation | 6 |  |  | |  |
|  |  | | Visited a mosque/religious person | 7 |  |  | |  |
|  |  | | Something else - INTERVIEWER FIND OUT & WRITE IN | 8 |  |  | |  |
|  |  | | I did not seek any help | 9 | ASK Q190 |  | |  |
|  |  | | Prefer not to say | 97 | GO TO Q191 |  | |  |
|  |  | | Don’t know | 98 |  |  | |  |

ASK Q190 IF THE WOMAN OR GIRL DID NOT SEEK HELP ON THE LAST TIME SHE EXPERIENCED PHYSICAL VIOLENCE IN THE WORKPLACE (CODE ‘9’ AT Q189). OTHERS GO TO Q191.

| Q190. | | SHOWCARD 69 **Why did you not seek help on the last time that you experienced physical violence in the workplace. Read out the statement or statements that apply.**  MULTICODE OK | | | | |  | |
| --- | --- | --- | --- | --- | --- | --- | --- | --- |
|  |  | |  |  |  |  | |  |
|  |  | | It was my fault | 1 |  |  | |  |
|  |  | | Shame | 2 |  |  | |  |
|  |  | | Fear of being excluded or shunned by colleagues | 3 |  |  | |  |
|  |  | | To maintain family honour | 4 |  |  | |  |
|  |  | | Fear that husband or partner would make me stop work | 5 |  |  | |  |
|  |  | | Fear of violence from husband or partner | 6 |  |  | |  |
|  |  | | It would negatively affect my chances of promotion | 7 |  |  | |  |
|  |  | | I did not think anyone would believe me | 8 |  |  | |  |
|  |  | | I did not see the point | 9 |  |  | |  |
|  |  | | Other reason - INTERVIEWER FIND OUT & WRITE IN | 10 |  |  | |  |
|  |  | | None of these | 11 |  |  | |  |
|  |  | | Prefer not to say | 97 |  |  | |  |
|  |  | | Don’t know | 98 |  |  | |  |

ASK Q191 IF THE WOMAN OR GIRL EXPERIENCED VERBAL VIOLENCE IN THE WORKPLACE (ASPECT A CODED ‘1’ [YES] AT Q180. OTHERS GO TO Q195.

| Q191. | | SHOWCARD 70 **Thinking about the verbal violence you experienced in your workplace, which, if any, of these things happened? Read out the statement or statements that apply.**  MULTICODE OKAY | | | | |  | |
| --- | --- | --- | --- | --- | --- | --- | --- | --- |
|  |  | |  |  |  |  | |  |
|  |  | | You were insulted or made to feel bad | 1 |  |  | |  |
|  |  | | You were belittled or humiliated in front of other people | 2 |  |  | |  |
|  |  | | The person who did this did things to frighten or intimidate me | 3 |  |  | |  |
|  |  | | My work was undervalued or dismissed | 4 |  |  | |  |
|  |  | | I was told if I got pregnant I would be fired | 5 |  |  | |  |
|  |  | | Other FIND OUT & WRITE IN | 6 |  |  | |  |
|  |  | | None of these | 7 |  |  | |  |
|  |  | | Prefer not to say | 97 |  |  | |  |
|  |  | | Don’t know | 98 |  |  | |  |

| Q192. | | SHOWCARD 71 **Thinking about the last time you experienced verbal violence in your workplace, who did this to you? Read out the category or categories that apply.**  MULTICODE OKAY | | | | |  | |
| --- | --- | --- | --- | --- | --- | --- | --- | --- |
|  |  | |  |  |  |  | |  |
|  |  | | A supervisor or manager | 1 |  |  | |  |
|  |  | | A work colleague at the same level as me | 2 |  |  | |  |
|  |  | | A work colleague in a less senior level than me | 3 |  |  | |  |
|  |  | | A client or customer | 4 |  |  | |  |
|  |  | | Someone else - INTERVIEWER FIND OUT & WRITE IN | 5 |  |  | |  |
|  |  | | None of these | 6 |  |  | |  |
|  |  | | Prefer not to say | 97 |  |  | |  |
|  |  | | Don’t know | 98 |  |  | |  |

| Q193. | | SHOWCARD 72 **Thinking about the last time you experienced verbal violence in your workplace, which, of any of these things did you do about it? Read out the statement or statements that apply.**  MULTICODE OKAY | | | | |  | |
| --- | --- | --- | --- | --- | --- | --- | --- | --- |
|  |  | |  |  |  |  | |  |
|  |  | | I requested help from my family | 1 | GO TO Q195 |  | |  |
|  |  | | I requested help from my friends | 2 |  |  | |  |
|  |  | | I reported the incident to my boss/supervisor/manager | 3 |  |  | |  |
|  |  | | Reported the incident to the police | 4 |  |  | |  |
|  |  | | I went to a hospital or health clinic | 5 |  |  | |  |
|  |  | | I went to a support organisation | 6 |  |  | |  |
|  |  | | Visited a mosque/religious leader | 7 |  |  | |  |
|  |  | | Something else - INTERVIEWER FIND OUT & WRITE IN | 8 |  |  | |  |
|  |  | | I did not seek any help | 9 | ASK Q194 |  | |  |
|  |  | | Prefer not to say | 97 | GO TO Q195 |  | |  |
|  |  | | Don’t know | 98 |  |  | |  |

ASK Q194 IF THE WOMAN OR GIRL DID NOT SEEK HELP ON THE LAST TIME SHE EXPERIENCED VERBAL VIOLENCE IN THE WORKPLACE (CODE ‘9’ AT Q194). OTHERS GO TO Q195.

| Q194. | | SHOWCARD 73 **Why did you not seek help on the last time that you experienced verbal violence in the workplace? Read out the statement or statements from this card that apply.**  MULTICODE OK | | | | |  | |
| --- | --- | --- | --- | --- | --- | --- | --- | --- |
|  |  | |  |  |  |  | |  |
|  |  | | It was my fault | 1 |  |  | |  |
|  |  | | Shame | 2 |  |  | |  |
|  |  | | Fear of being excluded or shunned by colleagues | 3 |  |  | |  |
|  |  | | To maintain family honour | 4 |  |  | |  |
|  |  | | Fear that husband or partner would make me stop work | 5 |  |  | |  |
|  |  | | Fear of violence from husband or partner | 6 |  |  | |  |
|  |  | | It would negatively affect my chances of promotion | 7 |  |  | |  |
|  |  | | I did not think anyone would believe me | 8 |  |  | |  |
|  |  | | I did not see the point | 9 |  |  | |  |
|  |  | | Other reason - INTERVIEWER FIND OUT & WRITE IN | 10 |  |  | |  |
|  |  | | None of these | 11 |  |  | |  |
|  |  | | Prefer not to say | 97 |  |  | |  |
|  |  | | Don’t know | 98 |  |  | |  |

ASK Q195 IF ANY ASPECT (A TO E) CODED ‘1’ [YES] AT Q180. IF ALL ASPECTS (A-E) CODED 2 [NO] GO TO Q206.

**Now think about all of the incidents of violence that you experienced in the workplace in the last 12 months.**

| Q195. | | **How many incidents did you experience?**  WRITE NUMBER | | | | |  | |
| --- | --- | --- | --- | --- | --- | --- | --- | --- |
|  |  | |  | ☐☐☐ |  |  | |  |
|  |  | |  |  |  |  | |  |

| Q196. | | **Were you required to take any time off work as a result of any of the incidences you experienced in the last 12 months?**  SINGLE CODE ONLY | | | | |  | |
| --- | --- | --- | --- | --- | --- | --- | --- | --- |
|  |  | |  |  |  |  | |  |
|  |  | | Yes | 1 | ASK Q197 |  | |  |
|  |  | | No | 2 | GO TO Q199 |  | |  |
|  |  | | Prefer not to say | 97 |  |  | |  |
|  |  | | Don’t know | 98 |  |  | |  |

ASK Q197 IF YES (CODE ‘1’) AT Q196. OTHERS GO TO Q199.

| Q197. | | **How many days did you miss work?**  SINGLE CODE ONLY | | | | |  | |
| --- | --- | --- | --- | --- | --- | --- | --- | --- |
|  |  | |  |  |  |  | |  |
|  |  | | CODE ‘1’ AND WRITE IN THE NUMBER OF DAYS | 1  ☐☐☐ | ask q198 |  | |  |
|  |  | | Prefer not to say | 97 | go to q199 |  | |  |
|  |  | | Don’t know | 98 |  |  | |  |

ASK Q198 IF CODE ‘1’ AT Q197. OTHERS GO TO Q199

| Q198. | | **And of the days you missed work because of the violence incidents in the last 12 months, did you get paid for those days that you missed?** IF YES ASK **Was that for all of the days or some of the days?**  SINGLE CODE ONLY | | | | |  | |
| --- | --- | --- | --- | --- | --- | --- | --- | --- |
|  |  | |  |  |  |  | |  |
|  |  | | Yes – all of these days | 1 |  |  | |  |
|  |  | | Yes – some of these days, if so how many days? CODE ‘2’ AND WRITE IN NUMBER OF DAYS | 2  ☐☐ |  |  | |  |
|  |  | | No – not paid for any of the days missed | 3 |  |  | |  |
|  |  | | Prefer not to say | 97 |  |  | |  |
|  |  | | Don’t know | 98 |  |  | |  |

ASK Q199 IF THE WOMAN OR GIRL HAS EXPERIENCED ANY VIOLENCE IN THE WORKPLACE (ANY ASPECT CODED ‘1’ [YES] AT Q180. IF ALL ASPECTS CODED ‘2’ [NO] GO TO Q206.

| Q199. | | **Did you have to stop doing household tasks because of any of these violent incidents in the workplace in the last 12 months?**  SINGLE CODE ONLY | | | | |  | |
| --- | --- | --- | --- | --- | --- | --- | --- | --- |
|  |  | |  |  |  |  | |  |
|  |  | | Yes | 1 | ASK Q200 |  | |  |
|  |  | | No | 2 | GO TO Q201 |  | |  |
|  |  | | Prefer not to say | 97 |  |  | |  |
|  |  | | Don’t know | 98 |  |  | |  |

ASK Q200 IF YES (CODE ‘1’) AT Q199, OTHERS GO TO Q201

| Q200. | | **Please tell me how many days of housework you missed?**  SINGLE CODE ONLY | | | | |  | |
| --- | --- | --- | --- | --- | --- | --- | --- | --- |
|  |  | |  |  |  |  | |  |
|  |  | | CODE 1’ AND WRITE IN THE NUMBER OF DAYS | 1  ☐☐☐ |  |  | |  |
|  |  | | Prefer not to say | 97 |  |  | |  |
|  |  | | Don’t know | 98 |  |  | |  |

ASK Q201 IF THE WOMAN OR GIRL HAS SCHOOL-AGE CHILDREN LIVING WITH THEM (CODE ‘1’ AT Q95) OTHERS GO TO Q203.

| Q201. | | **Did any of your children miss school as a result of these incidents in the workplace…?**  SINGLE CODE ONLY | | | | |  | |
| --- | --- | --- | --- | --- | --- | --- | --- | --- |
|  |  | |  |  |  |  | |  |
|  |  | | Yes | 1 | ASK Q202 |  | |  |
|  |  | | No | 2 | GO TO Q203 |  | |  |
|  |  | | Prefer not to say | 97 |  |  | |  |
|  |  | | Don’t know | 98 |  |  | |  |

ASK Q202 IF CODE 1 AT Q201. OTHERS GO TO Q203.

| Q202. | | **How many days of school did your children miss?**  SINGLE CODE ONLY | | | | |  | |
| --- | --- | --- | --- | --- | --- | --- | --- | --- |
|  |  | |  |  |  |  | |  |
|  |  | | CODE ‘1’ AND WRITE IN THE NUMBER OF DAYS | 1  ☐☐☐ |  |  | |  |
|  |  | | Prefer not to say | 97 |  |  | |  |
|  |  | | Don’t know | 98 |  |  | |  |

ASK Q203 IF THE WOMAN OR GIRL HAS EXPERIENCED ANY VIOLENCE IN THE WORKPLACE (ANY ASPECT CODED ‘1’ [YES] AT Q180. IF ALL ASPECTS CODED ‘2’ [NO] GO TO Q206.

| Q203. | | SHOWCARD 74 **Did you require any of the following services? Read out the category or categories that apply.**  MULTICODE OKAY. | | | | |  | |
| --- | --- | --- | --- | --- | --- | --- | --- | --- |
|  |  | |  |  |  |  | |  |
|  |  | | Health services / medical treatment | 1 |  |  | |  |
|  |  | | Dentist | 2 |  |  | |  |
|  |  | | Safe transportation | 3 |  |  | |  |
|  |  | | Counselling | 4 |  |  | |  |
|  |  | | Police intervention | 5 |  |  | |  |
|  |  | | Legal aid / lawyer | 6 |  |  | |  |
|  |  | | Other services - INTERVIEWER FIND OUT & WRITE IN | 7 |  |  | |  |
|  |  | | None of these | 8 |  |  | |  |
|  |  | | Prefer not to say | 97 |  |  | |  |
|  |  | | Don’t know | 98 |  |  | |  |

ASK Q204 FOR EACH SERVICE USED AT Q203. OTHERS GO TO Q206.

| Q204. | | **How much did you have to pay for…?**  WRITE IN THE AMOUNT IN LOCAL CURRENCY FOR EACH SERVICE USED AT Q203. | | | | |  | |
| --- | --- | --- | --- | --- | --- | --- | --- | --- |
|  |  | |  |  |  |  | |  |
|  |  | | Health services or medical treatment | 1  ☐☐☐☐ |  |  | |  |
|  |  | | Dentist | 2  ☐☐☐☐ |  |  | |  |
|  |  | | Safe transportation | 3  ☐☐☐☐ |  |  | |  |
|  |  | | Counselling | 4  ☐☐☐☐ |  |  | |  |
|  |  | | Police intervention | 5  ☐☐☐☐ |  |  | |  |
|  |  | | Legal aid or a lawyer | 6  ☐☐☐☐ |  |  | |  |
|  |  | | Other services (SPECIFY) | 7  ☐☐☐☐ |  |  | |  |

ASK Q205 IF THE WOMAN OR GIRL HAS EXPERIENCED ANY VIOLENCE IN THE WORKPLACE (ANY ASPECT CODED 1 [YES] AT Q180. IF ALL ASPECTS CODED 2 [NO] GO TO Q206.

| Q205. | | **Thinking about the incidents of violence that you experienced in the workplace, which if any of these things did you do?**  READ OUT EACH STATEMENT. MULTICODE OKAY | | | | |  | |
| --- | --- | --- | --- | --- | --- | --- | --- | --- |
|  |  | |  |  |  |  | |  |
|  |  | | Changed job | 1 |  |  | |  |
|  |  | | Cut all ties with the person who was violent to you | 2 |  |  | |  |
|  |  | | Move to another to another village/town | 3 |  |  | |  |
|  |  | | OTHER (SPECIFY) | 4 |  |  | |  |
|  |  | | Prefer not to say | 97 |  |  | |  |
|  |  | | Don’t know | 98 |  |  | |  |

ASK ALL

| VIOLENCE IN EDUCATIONAL INSTITUTIONS |
| --- |

INTERVIEWER READ OUT:

**Women and girls may experience violence within education institutes.  I would like to ask you a few questions about such experiences. It is important to document these so that we can develop stronger laws, policies and appropriate services for women and girls in education.**

| Q206. | | **Can I just check - are you attending school, college or university now OR have done so in the last 12 months?**  SINGLE CODE ONLY | | | | |  | |
| --- | --- | --- | --- | --- | --- | --- | --- | --- |
|  |  | |  |  |  |  | |  |
|  |  | | Yes | 1 | ask Q207 |  | |  |
|  |  | | No | 2 |  |  | |  |
|  |  | | Prefer not to say | 97 | GO TO Q220 |  | |  |
|  |  | | Don’t know | 98 |  |  | |  |

ASK Q207 IF YES AT Q206. OTHERS GO TO Q220 IN THE NEXT SECTION

| Q207. | | SHOWCARD 75 **Did you experience any of the things shown on this card in your school, college or university in the last 12 months? Read out the category or categories from this card that apply.**  SINGLE CODE FOR EACH | | | | | | | |  | |
| --- | --- | --- | --- | --- | --- | --- | --- | --- | --- | --- | --- |
|  |  | |  | Yes | No | Prefer not to say |  |  |  | |  |
|  | A | | **Verbal Violence:**  Someone verbally intimidated, humiliated or insulted you? | 1 | 2 | 97 |  |  |  | |  |
|  | B | | **Physical violence:**  Someone slapped, pushed, punched, kicked, tried to burn you, pointed a gun, knife or any other weapon at you? | 1 | 2 | 97 |  |  |  | |  |
|  | C | | **Sexual Harassment:**  Someone verbally harassed you in a sexual manner? Leered at you? Made sexual jokes? Belittling/humiliating sexual comments? | 1 | 2 | 97 |  |  |  | |  |
|  | D | | **Sexual Assault**:  Someone grabbed, groped or otherwise touched you sexually without your consent | 1 | 2 | 97 |  |  |  | |  |
|  | E | | **Sexual Assault**:  Someone forced you to touch them or forced you (made you drunk, drugged you, threatened you so you could not refuse, physically forced you) to engage in sexual acts without your consent? | 1 | 2 | 97 |  |  |  | |  |

ASK Q208 IF ANY ASPECT OF VIOLENCE CODED YES (CODE 1) AT Q207. IF NONE OF THE ASPECTS ARE CODED YES, GO TO Q220 IN THE NEXT SECTION

| Q208. | | SHOWCARD 76 **And how often in the last 12 months did you experience…?**  READ OUT A TO E IF YES AT Q206. SINGLE CODE FOR EACH | | | | | | |  | |
| --- | --- | --- | --- | --- | --- | --- | --- | --- | --- | --- |
|  |  | |  | Once or twice | Between 3 and 5 times | Between 6 and 10 times | Between 11 and 20 times | More than 20 times | | Don’t know |
|  | a) | | Verbal violence (code a at Q207) in your educational institution? | 1 | 2 | 3 | 4 | 5 | | 98 |
|  | b) | | Physical violence (code b at Q207) in your educational institution? | 1 | 2 | 3 | 4 | 5 | | 98 |
|  | c) | | Sexual harassment (code c at Q207) in your educational institution? | 1 | 2 | 3 | 4 | 5 | | 98 |
|  | d) | | Sexual assault (code d at Q207) in your educational institution? | 1 | 2 | 3 | 4 | 5 | | 98 |
|  | e) | | Sexual assault (code e at Q207) in your educational institution? | 1 | 2 | 3 | 4 | 5 | | 98 |

ASK Q209 IF ASPECTS C, D OR E CODED AS ‘1’ AT Q207. OTHERS GO TO Q220.

| Q209. | | SHOWCARD 77 **Thinking about the most recent time you had unwanted sexual behaviour, which, if any, of the behaviours on the card happened?**  MULTICODE OKAY | | | |  | |
| --- | --- | --- | --- | --- | --- | --- | --- |
|  | A | Had derogatory nasty / humiliating or belittling sexual comments made about me, received repeated unwanted sexual advances or was threatened with sexual violence | 1 |  |  | |  |
|  | B | Touched inappropriately without my permission | 2 |  |  | |  |
|  | C | Physically forced to have sex against my will | 3 |  |  | |  |
|  | D | Forced to do something sexual that I found to be humiliating or degrading | 4 |  |  | |  |
|  | E | Had sex because of being afraid of what might happen if I did not consent | 5 |  |  | |  |
|  | F | Had sex because I was too drunk or drugged to refuse | 6 |  |  | |  |
|  |  | None of these | 7 |  |  | |  |
|  |  | Prefer not to say | 97 |  |  | |  |
|  |  | Don’t know | 98 |  |  | |  |

| Q210. | | **Thinking about the most recent time that you experienced such behaviours, when was this?**  SINGLE CODE ONLY | | | | |
| --- | --- | --- | --- | --- | --- | --- |
|  |  | |  |  |  |  |
|  |  | | Within the last week | 1 |  |  |
|  |  | | Within the last month | 2 |  |  |
|  |  | | Between 1 and 3 months ago | 3 |  |  |
|  |  | | Between 3 and 6 months ago | 4 |  |  |
|  |  | | In the last 12 months | 5 |  |  |
|  |  | | Longer than 12 months ago | 6 |  |  |
|  |  | | Prefer not to say | 97 |  |  |
|  |  | | Don’t know | 98 |  |  |

| Q211. | | SHOWCARD 78 **Thinking about the most recent time…who did it? Read out the category or categories that apply.**  MULTICODE OKAY | | | | |  | |
| --- | --- | --- | --- | --- | --- | --- | --- | --- |
|  |  | |  |  |  |  | |  |
|  |  | | A teacher | 1 |  |  | |  |
|  |  | | Another member of staff | 2 |  |  | |  |
|  |  | | Fellow student | 3 |  |  | |  |
|  |  | | Stranger | 4 |  |  | |  |
|  |  | | Someone else did it - INTERVIEWER FIND OUT & WRITE IN | 5 |  |  | |  |
|  |  | | Prefer not to say | 97 |  |  | |  |
|  |  | | Don’t know | 98 |  |  | |  |

| Q212. | | SHOWCARD 79 **Thinking about the last time you experienced such behaviours, which, of any of these things on this card did you do about it? Read out the statement or statements that apply.**  MULTICODE OKAY | | | | |  | |
| --- | --- | --- | --- | --- | --- | --- | --- | --- |
|  |  | |  |  |  |  | |  |
|  |  | | I requested help from my family | 1 | GO TO Q214 |  | |  |
|  |  | | I requested help from my friends | 2 |  |  | |  |
|  |  | | Reported to head teacher/supervisor/principal | 3 |  |  | |  |
|  |  | | Reported the incident to the police | 4 |  |  | |  |
|  |  | | I went to a hospital or health clinic | 5 |  |  | |  |
|  |  | | I went to a support organisation | 6 |  |  | |  |
|  |  | | I visited a mosque/religious person | 7 |  |  | |  |
|  |  | | I dropped out of the educational institute | 8 |  |  | |  |
|  |  | | Other - INTERVIEWER FIND OUT & WRITE IN | 9 |  |  | |  |
|  |  | | I did not seek any help | 10 | ASK Q213 |  | |  |
|  |  | | Prefer not to say | 97 | GO TO Q214 |  | |  |
|  |  | | Don’t know | 98 |  |  | |  |

aSK Q213 IF THE WOMAN OR GIRL DID NOT SEEK HELP AT Q212 (CODE ‘10’). OTHERS GO TO q214.

| Q213. | | SHOWCARD 80 **Why did you not seek help?**  OPEN QUESTION. MULTICODE OK | | | | |  | |
| --- | --- | --- | --- | --- | --- | --- | --- | --- |
|  |  | |  |  |  |  | |  |
|  |  | | It was my fault | 1 |  |  | |  |
|  |  | | Shame | 2 |  |  | |  |
|  |  | | Fear of being excluded or shunned by other students | 3 |  |  | |  |
|  |  | | Fear of being excluded by teachers | 4 |  |  | |  |
|  |  | | Fear that it would negatively affect my studies/results | 5 |  |  | |  |
|  |  | | To maintain family honour | 6 |  |  | |  |
|  |  | | Fear that husband or partner would make me stop school | 7 |  |  | |  |
|  |  | | Fear of violence from husband or partner | 8 |  |  | |  |
|  |  | | I did not think anyone would believe me | 9 |  |  | |  |
|  |  | | I did not see the point | 10 |  |  | |  |
|  |  | | Other reason - INTERVIEWER FIND OUT & WRITE IN | 11 |  |  | |  |
|  |  | | None of these | 12 |  |  | |  |
|  |  | | Prefer not to say | 97 |  |  | |  |
|  |  | | Don’t know | 98 |  |  | |  |

ASK IF THE WOMAN OR GIRL EXPERIENCED SEXUAL VIOLENCE (CODE ‘1’ AT C D OR E AT Q207). OTHERS GO TO Q220 IN THE NEXT SECTION.

| Q214. | | **Were you required to take any time off your studies as a result of unwanted behaviours of a sexual nature in the last 12 months?**  SINGLE CODE ONLY | | | | |  | |
| --- | --- | --- | --- | --- | --- | --- | --- | --- |
|  |  | |  |  |  |  | |  |
|  |  | | Yes | 1 | ASK Q215 |  | |  |
|  |  | | No | 2 | GO TO q216 |  | |  |
|  |  | | Prefer not to say | 97 |  |  | |  |
|  |  | | Don’t know | 98 |  |  | |  |

ASK Q215 IF YES (CODE 1) AT Q214. OTHERS GO TO Q216.

| Q215. | | **How many days did you have to take time off your studies as a result of unwanted behaviours of a sexual nature you experienced in the last 12 months?**  SINGLE CODE ONLY | | | | |  | |
| --- | --- | --- | --- | --- | --- | --- | --- | --- |
|  |  | |  |  |  |  | |  |
|  |  | | CODE ‘1’ AND WRITE IN THE NUMBER OF DAYS | 1  ☐☐☐ |  |  | |  |
|  |  | | Prefer not to say | 97 |  |  | |  |
|  |  | | Don’t know | 98 |  |  | |  |

ASK Q216 IF THE WOMAN OR GIRL EXPERIENCED SEXUAL VIOLENCE (CODES C, D OR E AT Q207). OTHERS GO TO Q220 IN THE NEXT SECTION.

| Q216. | | **Did you have to stop doing any of these things because of the unwanted behaviours of sexual nature you experienced in the last 12 months?**  READ OUT A TO N. SINGLE CODE FOR EACH.  IF CODE 1 OR CODE 2 USED ASK NUMBER OF DAYS AND WRITE IN. | | | | | | |  | |
| --- | --- | --- | --- | --- | --- | --- | --- | --- | --- | --- |
|  |  | |  | Fully Stopped | Days | Partially stopped (50%) | Days | No Effect | |  |
|  | a) | | Fetching water | 1 | ☐☐☐ | 2 | ☐☐☐ | 99 | |  |
|  | b) | | Fetching wood | 1 | ☐☐☐ | 2 | ☐☐☐ | 99 | |  |
|  | c) | | Caring for children | 1 | ☐☐☐ | 2 | ☐☐☐ | 99 | |  |
|  | d) | | Ironing | 1 | ☐☐☐ | 2 | ☐☐☐ | 99 | |  |
|  | e) | | Washing clothes | 1 | ☐☐☐ | 2 | ☐☐☐ | 99 | |  |
|  | f) | | Sweeping | 1 | ☐☐☐ | 2 | ☐☐☐ | 99 | |  |
|  | g) | | Washing dishes | 1 | ☐☐☐ | 2 | ☐☐☐ | 99 | |  |
|  | h) | | Washing vehicles | 1 | ☐☐☐ | 2 | ☐☐☐ | 99 | |  |
|  | i) | | Dispose garbage | 1 | ☐☐☐ | 2 | ☐☐☐ | 99 | |  |
|  | j) | | Cooking | 1 | ☐☐☐ | 2 | ☐☐☐ | 99 | |  |
|  | k) | | Shopping for household needs | 1 | ☐☐☐ | 2 | ☐☐☐ | 99 | |  |
|  | l) | | Running errands | 1 | ☐☐☐ | 2 | ☐☐☐ | 99 | |  |
|  | m) | | Taking care of livestock or poultry | 1 | ☐☐☐ | 2 | ☐☐☐ | 99 | |  |
|  | n) | | Making clothes for family | 1 | ☐☐☐ | 2 | ☐☐☐ | 99 | |  |

| Q217. | | **Did you require any of the services as a result of the unwanted behaviours of sexual nature you experienced in the last 12 months?**  READ OUT A to G. SINGLE CODE ONLY | | | | |  | |
| --- | --- | --- | --- | --- | --- | --- | --- | --- |
|  |  | |  |  |  |  | |  |
|  | a) | | Health services or medical treatment | 1 | ASK Q218 |  | |  |
|  | b) | | Dentist | 2 |  |  | |  |
|  | c) | | Safe transportation | 3 |  |  | |  |
|  | d) | | Counselling | 4 |  |  | |  |
|  | e) | | Police intervention | 5 |  |  | |  |
|  | f) | | Legal aid or a lawyer | 6 |  |  | |  |
|  | g) | | Other services - INTERVIEWER FIND OUT & WRITE IN | 7 |  |  | |  |
|  |  | | None of these | 8 | GO TO Q220 |  | |  |
|  |  | | Prefer not to say | 97 | ASK Q218 |  | |  |
|  |  | | Don’t know | 98 |  |  | |  |

ASK Q218 IF ANY SERVICE USED AT Q217 (CODES ‘1’ TO ‘7’ OR ‘97’ OR ‘98’). OTHERS GO TO Q220 IN THE NEXT SECTION.

| Q218. | | **Did you have to pay for any of these services as a result of unwanted behaviours of sexual nature you experienced at an educational institution in the last 12 months?**  READ OUT A TO H. SINGLE CODE FOR EACH | | | | | | |  | |
| --- | --- | --- | --- | --- | --- | --- | --- | --- | --- | --- |
|  |  | |  | Yes | No | Don’t know |  |  | |  |
|  | a) | | Health services or medical treatment | 1 | 2 | 98 |  |  | |  |
|  | b) | | Dentist | 1 | 2 | 98 |  |  | |  |
|  | c) | | Safe transportation | 1 | 2 | 98 |  |  | |  |
|  | d) | | Counselling | 1 | 2 | 98 |  |  | |  |
|  | e) | | Police intervention | 1 | 2 | 98 |  |  | |  |
|  | f) | | Legal aid or a lawyer | 1 | 2 | 98 |  |  | |  |
|  | g) | | Private tutoring or education | 1 | 2 | 98 |  |  | |  |
|  | h) | | Other services - INTERVIEWER FIND OUT & WRITE IN | 1 | 2 | 98 |  |  | |  |

ASK FOR EACH SERVICE RESPONDENT HAD TO PAY FOR AT Q218 (CODED AS ‘1’). IF NO SERVICES PAID FOR (ALL SERVICES CODED AS ‘2’ OR ‘98’), GO TO Q220 IN THE NEXT SECTION.

| Q219. | | **How much did you have to pay for…?**  WRITE IN THE AMOUNT IN LOCAL CURRENCY FOR EACH SERVICE PAID FOR AT Q218. | | | | |  | |
| --- | --- | --- | --- | --- | --- | --- | --- | --- |
|  |  | |  |  |  |  | |  |
|  |  | | Health services or medical treatment | 1  ☐☐☐☐ |  |  | |  |
|  |  | | Dentist | 2  ☐☐☐☐ |  |  | |  |
|  |  | | Safe transportation | 3  ☐☐☐☐ |  |  | |  |
|  |  | | Counselling | 4  ☐☐☐☐ |  |  | |  |
|  |  | | Police intervention | 5  ☐☐☐☐ |  |  | |  |
|  |  | | Legal aid or a lawyer | 6  ☐☐☐☐ |  |  | |  |
|  |  | | Other services - INTERVIEWER FIND OUT & WRITE IN | 7  ☐☐☐☐ |  |  | |  |

ASK ALL

| VIOLENCE IN PUBLIC SPACES |
| --- |

**Women and girls often report experiencing different violent behaviours in public spaces, like market streets, and on public transportation. I would like to ask you a few questions about such experiences. These are important to document so that we can develop stronger laws to protect women in public spaces.**

| Q220. | | **Have you ever experienced any of these negative behaviours in your lifetime in a public space? For example, on a bus, train, metro, market, street, public building, on your way to the toilet or to collect firewood.**  SINGLE CODE ONLY | | | | |  | |
| --- | --- | --- | --- | --- | --- | --- | --- | --- |
|  |  | |  |  |  |  | |  |
|  |  | | Yes | 1 | ask q221 |  | |  |
|  |  | | No | 2 | go to q247 |  | |  |
|  |  | | Prefer not to say | 97 |  |  | |  |
|  |  | | Don’t know | 98 |  |  | |  |

ask q221 if yes (CODE ‘1’ at q220). others go to Q247.

| Q221. | | SHOWCARD 81 **Which, if any, of these behaviours have you experienced in public spaces in the last 12 months? Read out the category or categories from this card that apply.**  SINGLE CODE FOR EACH | | | | | | |  | |  |  |
| --- | --- | --- | --- | --- | --- | --- | --- | --- | --- | --- | --- | --- |
|  |  | |  | Yes | No | Prefer not to say |  |  | |  | |  |
|  | A | | **Verbal Violence:**  Someone verbally intimidated, humiliated or insulted you? | 1 | 2 | 97 |  |  | |  | |  |
|  | B | | **Physical violence:**  Someone slapped, pushed, punched, kicked, tried to burn you, pointed a gun, knife or any other weapon at you? | 1 | 2 | 97 |  |  | |  | |  |
|  | C | | **Sexual Harassment:**  Someone verbally harassed you in a sexual manner? Leered at you? Made sexual jokes? Belittling/humiliating sexual comments? | 1 | 2 | 97 |  |  | |  | |  |
|  | D | | **Sexual Assault:**  Someone grabbed, groped or otherwise touched you sexually without your consent | 1 | 2 | 97 |  |  | |  | |  |
|  | E | | **Sexual Assault:**  Someone forced you to touch them or forced you (made you drunk, drugged you, threatened you so you could not refuse, physically forced you) to engage in sexual acts without your consent? | 1 | 2 | 97 |  |  | |  | |  |

ASK FOR EACH ASPECT CODED ‘1’ YES AT Q221. IF ALL CODE ‘2’ AT Q221 GO TO Q247

| Q222. | | SHOWCARD 82 **And how often in the last 12 months did you experience the following things?**  READ OUT A TO E IF YES AT Q221. SINGLE CODE FOR EACH | | | | | |  | |
| --- | --- | --- | --- | --- | --- | --- | --- | --- | --- |
|  |  |  | Once or twice | Between 3 and 5 times | Between 6 and 10 times | Between 11 and 20 times | More than 20 times | | Don’t know |
|  | a) | Verbal violence in public spaces? | 1 | 2 | 3 | 4 | 5 | | 98 |
|  | b) | Physical violence in public spaces? | 1 | 2 | 3 | 4 | 5 | | 98 |
|  | c) | Sexual harassment in public spaces? | 1 | 2 | 3 | 4 | 5 | | 98 |
|  | d) | Sexual assault in public spaces (D in Q221)? | 1 | 2 | 3 | 4 | 5 | | 98 |
|  | e) | Sexual assault in public spaces (E in Q221? | 1 | 2 | 3 | 4 | 5 | | 98 |

ASK Q223 IF C, D OR E CODED AS ‘1’ AT Q221. OTHERS GO TO Q229.

| Q223. | | SHOWCARD 83 **Thinking about the most recent incident** **in which you experienced unwanted sexual behaviour which, if any, of the following behaviours happened?**  MULTICODE OKAY | | | |  | |
| --- | --- | --- | --- | --- | --- | --- | --- |
|  |  | Had derogatory nasty / humiliating/belittling sexual comments made about me, received repeated unwanted sexual advances or was threatened with sexual violence | 1 | ASK Q224 |  | |  |
|  |  | Touched inappropriately without my permission | 2 |  |  | |  |
|  |  | Physically forced to have sex against my will | 3 |  |  | |  |
|  |  | Forced to do something sexual that I found to be humiliating or degrading | 4 |  |  | |  |
|  |  | Had sex because of being afraid of what might happen if I did not consent | 5 |  |  | |  |
|  |  | Had sex because I was too drunk or drugged to refuse | 6 |  |  | |  |
|  |  | None of these | 7 | GO TO Q229 |  | |  |
|  |  | Prefer not to say | 97 |  |  | |  |
|  |  | Don’t know | 98 |  |  | |  |

ASK Q224 IF ANSWER GIVEN AT Q223 (CODES ‘1’ TO ‘6’). OTHERS GO TO Q229.

| Q224. | | SHOWCARD 84 **And when was this?**  SINGLE CODE ONLY | | | | |  | |
| --- | --- | --- | --- | --- | --- | --- | --- | --- |
|  |  | |  |  |  |  | |  |
|  |  | | Within the last week | 1 |  |  | |  |
|  |  | | Within the last month | 2 |  |  | |  |
|  |  | | Between 1 and 3 months ago | 3 |  |  | |  |
|  |  | | Between 3 and 6 months ago | 4 |  |  | |  |
|  |  | | In the last 12 months | 5 |  |  | |  |
|  |  | | More than 12 months ago | 6 |  |  | |  |
|  |  | | Prefer not to say | 97 |  |  | |  |
|  |  | | Don’t know | 98 |  |  | |  |

| Q225. | | SHOWCARD 85 **Where was this?**  MULTICODE OKAY | | | | |  | |
| --- | --- | --- | --- | --- | --- | --- | --- | --- |
|  |  | |  |  |  |  | |  |
|  |  | | On a bus | 1 |  |  | |  |
|  |  | | On a microbus | 2 |  |  | |  |
|  |  | | On the metro | 3 |  |  | |  |
|  |  | | On a train | 4 |  |  | |  |
|  |  | | In a taxi | 5 |  |  | |  |
|  |  | | Outdoors in your local community | 6 |  |  | |  |
|  |  | | Outdoors in another community | 7 |  |  | |  |
|  |  | | In a market | 8 |  |  | |  |
|  |  | | In a street | 9 |  |  | |  |
|  |  | | In a public building | 10 |  |  | |  |
|  |  | | Other location - INTERVIEWER FIND OUT & WRITE IN | 11 |  |  | |  |
|  |  | | Prefer not to say | 97 |  |  | |  |
|  |  | | Don’t know | 98 |  |  | |  |

| Q226. | | **Did you know the person who did this to you?**  SINGLE CODE ONLY | | | | |  | |
| --- | --- | --- | --- | --- | --- | --- | --- | --- |
|  |  | |  |  |  |  | |  |
|  |  | | Yes | 1 |  |  | |  |
|  |  | | No | 2 |  |  | |  |
|  |  | | Prefer not to say | 97 |  |  | |  |
|  |  | | Don’t know | 98 |  |  | |  |

| Q227. | | SHOWCARD 86 **Who did this to you?**  MULTICODE OKAY | | | | |  | |
| --- | --- | --- | --- | --- | --- | --- | --- | --- |
|  |  | |  |  |  |  | |  |
|  |  | | A market seller | 1 |  |  | |  |
|  |  | | Shopkeeper | 2 |  |  | |  |
|  |  | | Security guard/officer | 3 |  |  | |  |
|  |  | | Military, paramilitary or rebel force member | 4 |  |  | |  |
|  |  | | Passenger on public transport | 5 |  |  | |  |
|  |  | | Bus driver or conductor | 6 |  |  | |  |
|  |  | | Taxi driver | 7 |  |  | |  |
|  |  | | Police officer | 8 |  |  | |  |
|  |  | | Prefer not to say | 97 |  |  | |  |
|  |  | | Don’t know | 98 |  |  | |  |

| Q228. | | **Was this person alone or with other men?**  SINGLE CODE ONLY | | | | |  | |
| --- | --- | --- | --- | --- | --- | --- | --- | --- |
|  |  | |  |  |  |  | |  |
|  |  | | Alone | 1 |  |  | |  |
|  |  | | With other men | 2 |  |  | |  |
|  |  | | Prefer not to say | 97 |  |  | |  |
|  |  | | Don’t know | 98 |  |  | |  |

ASK q229 IF the WOMAN OR GIRL EXPERIENCED sexual VIOLENCE IN PUBLIC (CODE ‘1’ at c, d or e at q221). OTHERS GO TO Q247.

| Q229. | | SHOWCARD 87 **Thinking about the most recent time that you experienced unwanted sexual behaviours in a public place, which, if any of the following things did you do about it?**  MULTICODE OKAY | | | | |  | |
| --- | --- | --- | --- | --- | --- | --- | --- | --- |
|  |  | |  |  |  |  | |  |
|  |  | | I requested help from my family | 1 | go to q231 |  | |  |
|  |  | | I requested help from my friends | 2 |  |  | |  |
|  |  | | Reported the incident to the police | 3 |  |  | |  |
|  |  | | I went to a hospital or health clinic | 4 |  |  | |  |
|  |  | | I went to a support organisation | 5 |  |  | |  |
|  |  | | Visited a mosque/religious person | 6 |  |  | |  |
|  |  | | Other - INTERVIEWER FIND OUT & WRITE IN | 7 |  |  | |  |
|  |  | | I did not seek any help | 8 | ask q230 |  | |  |
|  |  | | Prefer not to say | 97 | go to q231 |  | |  |
|  |  | | Don’t know | 98 |  |  | |  |

ASK Q230 IF CODE ‘8’ used AT Q229. OTHERS GO TO Q231.

| Q230. | | SHOWCARD 88 **Why did you not seek help? Read out the statement or statements from this card that apply.**  MULTICODE OK | | | | |  | |
| --- | --- | --- | --- | --- | --- | --- | --- | --- |
|  |  | |  |  |  |  | |  |
|  |  | | It was my fault | 1 |  |  | |  |
|  |  | | Shame | 2 |  |  | |  |
|  |  | | Fear of being excluded or shunned by community | 3 |  |  | |  |
|  |  | | To maintain family honour | 4 |  |  | |  |
|  |  | | Fear of my husband’s or partner’s reaction | 5 |  |  | |  |
|  |  | | Fear of violence from other family members | 6 |  |  | |  |
|  |  | | I did not think anyone would believe me | 7 |  |  | |  |
|  |  | | I did not see the point | 8 |  |  | |  |
|  |  | | I did not know where to get help | 9 |  |  | |  |
|  |  | | Other reason - INTERVIEWER FIND OUT & WRITE IN | 10 |  |  | |  |
|  |  | | None of these | 11 |  |  | |  |
|  |  | | Prefer not to say | 97 |  |  | |  |
|  |  | | Don’t know | 98 |  |  | |  |

ASK q231 IF the woman OR GIRL WORKs (code ‘1’ at q57A). OTHERS GO TO Q234.

| Q231. | | **Were you required to take any time off work as a result of these unwanted sexual behaviours in the last 12 months?**  SINGLE CODE ONLY | | | | |  | |
| --- | --- | --- | --- | --- | --- | --- | --- | --- |
|  |  | |  |  |  |  | |  |
|  |  | | Yes | 1 | ask q232 |  | |  |
|  |  | | No | 2 | go to q234 |  | |  |
|  |  | | Prefer not to say | 97 |  |  | |  |
|  |  | | Don’t know | 98 |  |  | |  |

ASK Q232 IF THE WOMAN OR GIRL TOOK TIME OFF WORK (CODE ‘1’ AT Q231). OTHERS GO TO Q234.

| Q232. | | **How many days did you miss work due to these violent incidents in the last 12 months?**  SINGLE CODE ONLY | | | | |  | |
| --- | --- | --- | --- | --- | --- | --- | --- | --- |
|  |  | |  |  |  |  | |  |
|  |  | | WRITE IN NUMBER OF DAYS | 1  ☐☐☐ |  |  | |  |
|  |  | | Prefer not to say | 97 |  |  | |  |
|  |  | | Don’t know | 98 |  |  | |  |

| Q233. | | **And of the days you missed work because of these incidents, did you get paid for those days that you missed?**  IF YES ASK **And was that for all of the days or some of the days?**  SINGLE CODE ONLY | | | | |  | |
| --- | --- | --- | --- | --- | --- | --- | --- | --- |
|  |  | |  |  |  |  | |  |
|  |  | | Yes – all of these days | 1 |  |  | |  |
|  |  | | Yes – some of these days, if so how many? CODE ‘2’ AND WRITE IN NUMBER OF DAYS | 2  ☐☐☐ |  |  | |  |
|  |  | | No – not paid for any of the days missed | 3 |  |  | |  |
|  |  | | Prefer not to say | 97 |  |  | |  |
|  |  | | Don’t know | 98 |  |  | |  |

ASK Q234 IF the woman OR GIRL HAS SCHOOL-AGE CHILDREN (CODE ‘1’ AT Q95). OTHERS GO TO Q236.

| Q234. | | **Did your children miss school because of any incident of violence you experienced in a public space in the last 12 months?**  SINGLE CODE ONLY | | | | |  | |
| --- | --- | --- | --- | --- | --- | --- | --- | --- |
|  |  | |  |  |  |  | |  |
|  |  | | Yes | 1 | ASK Q235 |  | |  |
|  |  | | No | 2 | GO TO Q236 |  | |  |
|  |  | | Don’t know | 98 |  |  | |  |
|  |  | | Prefer not to say | 97 |  |  | |  |

ASK Q235 IF YES (CODE ‘1’) AT Q234. OTHERS GO TO Q236.

| Q235. | | **How many days of school did your children miss?**  SINGLE CODE ONLY | | | | |  | |
| --- | --- | --- | --- | --- | --- | --- | --- | --- |
|  |  | |  |  |  |  | |  |
|  |  | | CODE ‘1’ AND WRITE IN THE NUMBER OF DAYS | 1  ☐☐☐ |  |  | |  |
|  |  | | Don’t know | 98 |  |  | |  |
|  |  | | Prefer not to say | 97 |  |  | |  |

ASK q236 IF the WOMAN OR GIRL EXPERIENCED SEXUAL VIOLENCE IN PUBLIC (ASPECT C, D, OR E CODED ‘1’ at q221). OTHERS GO TO Q247.

| Q236. | | **Did you have to stop doing any of these things as a result of unwanted sexual behaviour you experienced in public spaces in the last 12 months?**  READ OUT A TO N. SINGLE CODE FOR EACH  IF CODE 1 OR CODE 2 USED, ASK NUMBER OF DAYS AND WRITE IN. | | | | | | |  | |
| --- | --- | --- | --- | --- | --- | --- | --- | --- | --- | --- |
|  |  | |  | Fully Stopped | Days | Partially stopped (50%) | Days | No Effect | |  |
|  | a) | | Fetching water | 1 | ☐☐☐ | 2 | ☐☐☐ | 99 | |  |
|  | b) | | Fetching wood | 1 | ☐☐☐ | 2 | ☐☐☐ | 99 | |  |
|  | c) | | Caring for children | 1 | ☐☐☐ | 2 | ☐☐☐ | 99 | |  |
|  | d) | | Ironing | 1 | ☐☐☐ | 2 | ☐☐☐ | 99 | |  |
|  | e) | | Washing clothes | 1 | ☐☐☐ | 2 | ☐☐☐ | 99 | |  |
|  | f) | | Sweeping | 1 | ☐☐☐ | 2 | ☐☐☐ | 99 | |  |
|  | g) | | Washing dishes | 1 | ☐☐☐ | 2 | ☐☐☐ | 99 | |  |
|  | h) | | Washing vehicles | 1 | ☐☐☐ | 2 | ☐☐☐ | 99 | |  |
|  | i) | | Disposing garbage | 1 | ☐☐☐ | 2 | ☐☐☐ | 99 | |  |
|  | j) | | Cooking | 1 | ☐☐☐ | 2 | ☐☐☐ | 99 | |  |
|  | k) | | Shopping for household needs | 1 | ☐☐☐ | 2 | ☐☐☐ | 99 | |  |
|  | l) | | Running errands | 1 | ☐☐☐ | 2 | ☐☐☐ | 99 | |  |
|  | m) | | Taking care of livestock or poultry | 1 | ☐☐☐ | 2 | ☐☐☐ | 99 | |  |
|  | n) | | Making clothes for family | 1 | ☐☐☐ | 2 | ☐☐☐ | 99 | |  |

| Q237. | | SHOWCARD 89 **Did you require any of the following services as a result of the unwanted sexual behaviours you experienced in the last 12 months? Read out the category or categories from this card that apply.**  MULTICODE OKAY. | | | | |  | |
| --- | --- | --- | --- | --- | --- | --- | --- | --- |
|  |  | |  |  |  |  | |  |
|  |  | | Health services or medical treatment | 1 | ASK Q238 |  | |  |
|  |  | | Dentist | 2 |  |  | |  |
|  |  | | Safe transportation | 3 |  |  | |  |
|  |  | | Counselling | 4 |  |  | |  |
|  |  | | Police intervention | 5 |  |  | |  |
|  |  | | Legal aid or a lawyer | 6 |  |  | |  |
|  |  | | Other services - INTERVIEWER FIND OUT & WRITE IN | 7 |  |  | |  |
|  |  | | None of these | 8 |  |  | |  |
|  |  | | Prefer not to say | 97 | GO TO Q240 |  | |  |
|  |  | | Don’t know | 98 |  |  | |  |

ASK Q238 FOR EACH SERVICE CODED AT Q237 (CODES ‘1’ TO ‘7’). OTHERS GO TO Q240.

| Q238. | | **Did you have to pay for these services?**  READ OUT A TO G IF SERVICE USED AT Q237. SINGLE CODE FOR EACH. | | | | | | |  | |
| --- | --- | --- | --- | --- | --- | --- | --- | --- | --- | --- |
|  |  | |  | Yes | No | Don’t know |  |  | |  |
|  | a) | | Health services or medical treatment | 1 | 2 | 98 |  |  | |  |
|  | b) | | Dentist | 1 | 2 | 98 |  |  | |  |
|  | c) | | Safe transportation | 1 | 2 | 98 |  |  | |  |
|  | d) | | Counselling | 1 | 2 | 98 |  |  | |  |
|  | e) | | Police intervention | 1 | 2 | 98 |  |  | |  |
|  | f) | | Legal aid or a lawyer | 1 | 2 | 98 |  |  | |  |
|  | g) | | Other services - INTERVIEWER FIND OUT & WRITE IN | 1 | 2 | 98 |  |  | |  |

ASK Q239 FOR EACH SERVICE THE WOMAN OR GIRL HAD TO PAY FOR AT Q238. IF NO SERVICES PAID FOR GO TO Q240.

| Q239. | | **How much did you have to pay for…?**  WRITE IN THE AMOUNT IN LOCAL CURRENCY FOR EACH SERVICE PAID FOR AT Q238. | | | | |  | |
| --- | --- | --- | --- | --- | --- | --- | --- | --- |
|  |  | |  |  |  |  | |  |
|  |  | | Health services or medical treatment | 1  ☐☐☐☐ |  |  | |  |
|  |  | | Dentist | 2  ☐☐☐☐ |  |  | |  |
|  |  | | Safe transportation | 3  ☐☐☐☐ |  |  | |  |
|  |  | | Counselling | 4  ☐☐☐☐ |  |  | |  |
|  |  | | Police intervention | 5  ☐☐☐☐ |  |  | |  |
|  |  | | Legal aid or a lawyer | 6  ☐☐☐☐ |  |  | |  |
|  |  | | Other services - INTERVIEWER FIND OUT & WRITE IN | 7  ☐☐☐☐ |  |  | |  |

ASK q240 IF the WOMAN OR GIRL EXPERIENCED VIOLENCE oN PUBLIC transport (CODEs ‘1’ to ‘5’ at q225). OTHERS GO TO Q247.

| Q240. | | **As a result of any unwanted sexual behaviours you experienced on public transport, did you change the route you normally use for transportation?**  SINGLE CODE ONLY | | | | |  | |
| --- | --- | --- | --- | --- | --- | --- | --- | --- |
|  |  | |  |  |  |  | |  |
|  |  | | Yes | 1 | ask q241 |  | |  |
|  |  | | No | 2 | go to q243 |  | |  |
|  |  | | Prefer not to say | 97 |  |  | |  |
|  |  | | Don’t know | 98 |  |  | |  |

ASK Q241 IF YES (CODE ‘1’ AT Q240. OTHERS GO TO Q243.

| Q241. | | **Did changing the route take you longer to get to where you needed to go?**  SINGLE CODE ONLY | | | | |  | |
| --- | --- | --- | --- | --- | --- | --- | --- | --- |
|  |  | |  |  |  |  | |  |
|  |  | | Yes | 1 | ask q242 |  | |  |
|  |  | | No | 2 | go to q243 |  | |  |
|  |  | | Prefer not to say | 97 |  |  | |  |
|  |  | | Don’t know | 98 |  |  | |  |

ASK Q242 IF YES (CODE ‘1’ AT Q241). OTHERS GO TO Q243.

| Q242. | | **How much extra time in minutes does it take you on average per day?**  SINGLE CODE ONLY | | | | |  | |
| --- | --- | --- | --- | --- | --- | --- | --- | --- |
|  |  | |  |  |  |  | |  |
|  |  | | WRITE IN NUMBER OF MINUTES | 1  ☐☐☐ |  |  | |  |
|  |  | | Prefer not to say | 97 |  |  | |  |
|  |  | | Don’t know | 98 |  |  | |  |

ASK q243 IF the WOMAN EXPERIENCED VIOLENCE ON PUBLIC transport (CODEs ‘1’ to ‘5’ q225). OTHERS GO TO Q247.

| Q243. | | **Does anyone else now accompany you on public transport as a result of the unwanted sexual behaviours you experienced in the last 12 months?**  SINGLE CODE ONLY | | | | |  | |
| --- | --- | --- | --- | --- | --- | --- | --- | --- |
|  |  | |  |  |  |  | |  |
|  |  | | Yes | 1 | ask q244 |  | |  |
|  |  | | No | 2 | go to q247 |  | |  |
|  |  | | Prefer not to say | 97 |  |  | |  |
|  |  | | Don’t know | 98 |  |  | |  |

ASK Q244 IF YES (CODE ‘1’ AT Q243). others go to q247

| Q244. | | **Who accompanies you?**  SINGLE CODE ONLY | | | | |  | |
| --- | --- | --- | --- | --- | --- | --- | --- | --- |
|  |  | |  |  |  |  | |  |
|  |  | | CODE ‘1’ AND WRITE IN | 1 |  |  | |  |
|  |  | | Prefer not to say | 97 |  |  | |  |
|  |  | | Don’t know | 98 |  |  | |  |

| Q245. | | **How much extra time, on average, would you say it takes this person or people to accompany you on public transport on each time that they do this?**  SINGLE CODE ONLY | | | | |  | |
| --- | --- | --- | --- | --- | --- | --- | --- | --- |
|  |  | |  |  |  |  | |  |
|  |  | | CODE ‘1’ AND WRITE IN THE NUMBER OF MINUTES | 1  ☐☐☐ |  |  | |  |
|  |  | | Prefer not to say | 97 |  |  | |  |
|  |  | | Don’t know | 98 |  |  | |  |

| Q246. | | **What is this person’s job or profession?**  SINGLE CODE ONLY | | | | |  | |
| --- | --- | --- | --- | --- | --- | --- | --- | --- |
|  |  | |  |  |  |  | |  |
|  |  | | CODE ‘1’ AND WRITE IN THE PROFESSION | 1 |  |  | |  |
|  |  | | Prefer not to say | 97 |  |  | |  |
|  |  | | Don’t know | 98 |  |  | |  |

ask all

| IMPACT AND SEEKING HELP |
| --- |

ASK Q247 IF WOMAN OR GIRL HAS EVER EXPERIENCED ANY VIOLENCE (CODES ‘2-6’ AT ANY ASPECT OF Q102, Q103, Q142, Q144 OR CODE ‘1’ AT ANY ASPECT OF Q180, Q207, Q221). OTHERS GO TO Q252.

**I would like to ask about the impact of any of the violence you may have experienced in the last 12 months.**

| Q247. | | SHOWCARD 90 **Due to any of the violence you experienced in the last 12 months, did you suffer from any of the following? Read out the things from the card which apply.**  PROBE: **Anything else?**  MULTICODE OKAY | | | | |  | |
| --- | --- | --- | --- | --- | --- | --- | --- | --- |
|  |  | |  |  |  |  | |  |
|  |  | | Depression | 1 |  |  | |  |
|  |  | | Anxiety | 2 |  |  | |  |
|  |  | | Panic attacks | 3 |  |  | |  |
|  |  | | Loss of self-confidence | 4 |  |  | |  |
|  |  | | Feeling vulnerable | 5 |  |  | |  |
|  |  | | Difficulty in sleeping | 6 |  |  | |  |
|  |  | | Difficulty concentrating | 7 |  |  | |  |
|  |  | | Difficulties in relationships | 8 |  |  | |  |
|  |  | | Other INTERVIEWER FIND OUT & WRITE IN | 9 |  |  | |  |
|  |  | | None of these | 10 |  |  | |  |
|  |  | | Prefer not to say | 97 |  |  | |  |
|  |  | | Don’t know | 98 |  |  | |  |

| Q248. | | SHOWCARD 91 **Because of any violence you experienced in the last 12 months, did you reduce or stop doing any of the things shown on this card? Read out the things from this card which apply.** | | | | |  |  |
| --- | --- | --- | --- | --- | --- | --- | --- | --- |
|  | | |  |  | |  | | |
|  |  | | Attending clubs, groups or societies | 1 |  | | | |
|  |  | | Studying | 2 |  | | | |
|  |  | | Working | 3 |  | | | |
|  |  | | Visiting family | 4 |  | | | |
|  |  | | Visiting friends | 5 |  | | | |
|  |  | | Attending social events (e.g. cinema, dances, etc.) | 6 |  | | | |
|  |  | | Attending Religious Services | 7 |  | | | |
|  |  | | Walking at night time | 8 |  | | | |
|  |  | | Walking in the day time | 9 |  | | | |
|  |  | | Helping friends or neighbours | 10 |  | | | |
|  |  | | Playing with your children | 11 |  | | | |
|  |  | | Other - INTERVIEWER FIND OUT & WRITE IN) | 12 |  | | | |
|  | | | Prefer not to say | 97 |  | | | |
|  | | | Don’t know | 98 |  | | | |

| Q249. | | **Given your experiences of violence in the last 12 months, did you seek help from any services, family or friends?**  SINGLE CODE ONLY. | | | |  | |
| --- | --- | --- | --- | --- | --- | --- | --- |
|  |  |  |  |  | |  | |
|  |  | Yes | 1 | ASK Q250 | |  | |
|  |  | No | 2 | go to q251 | |  | |
|  |  | Prefer not to say | 97 | go to q252 | |  | |
|  |  | Don’t know | 98 |  |  |  | |

ASK Q250 IF CODE ‘1’ AT Q249. OTHERS GO TO Q251 OR Q252 AS APPROPRIATE FROM Q249.

| Q250. | | SHOWCARD 92 **Which sources of help did you access? Read out the category or categories from this card which apply to you.**  MULTICODE OK. | | | | | |  | |
| --- | --- | --- | --- | --- | --- | --- | --- | --- | --- |
|  |  | |  |  |  | |  | |  |
|  | A | | Police | 1 | |  |  | |  |
|  | B | | Court | 2 | |  |  | |  |
|  | C | | Counselling | 3 | |  |  | |  |
|  | D | | Health clinic, doctor or hospital | 4 | |  |  | |  |
|  | E | | Religious leaders or group | 5 | |  |  | |  |
|  | F | | Cultural leader | 6 | |  |  | |  |
|  | G | | Spouse or partner | 7 | |  |  | |  |
|  | H | | Birth family | 8 | |  |  | |  |
|  | I | | Other family | 9 | |  |  | |  |
|  | J | | Neighbours | 10 | |  |  | |  |
|  | K | | Friends | 11 | |  |  | |  |
|  | L | | Other sources of help - INTERVIEWER FIND OUT & WRITE IN | 12 | |  |  | |  |
|  |  | | Prefer not to say | 97 | |  |  | |  |
|  |  | | Don’t know | 98 | |  |  | |  |

ASK Q251 IF CODE ‘2’ AT Q249. OTHERS GO TO Q252.

| Q251. | | SHOWCARD 93 **Why did you not seek help or support? Read out the category or categories from this card which apply.** MULTICODE OK. | | | | | |  | |
| --- | --- | --- | --- | --- | --- | --- | --- | --- | --- |
|  |  | |  |  |  | |  | |  |
|  |  | | Guilt | 1 | |  |  | |  |
|  |  | | Shame | 2 | |  |  | |  |
|  |  | | Cost | 3 | |  |  | |  |
|  |  | | Prevented by spouse or partner | 4 | |  |  | |  |
|  |  | | Prevented by someone else | 5 | |  |  | |  |
|  |  | | Didn’t know of anyone who could help | 6 | |  |  | |  |
|  |  | | Didn’t want anyone to know | 7 | |  |  | |  |
|  |  | | Feared the perpetrator would seek revenge | 8 | |  |  | |  |
|  |  | | Feared additional violence by others | 9 | |  |  | |  |
|  |  | | Feared I would be excluded by family | 10 | |  |  | |  |
|  |  | | Feared I would be excluded by community | 11 | |  |  | |  |
|  |  | | Didn’t see the point | 12 | |  |  | |  |
|  |  | | Didn’t need any services or help | 13 | |  |  | |  |
|  |  | | Other reason - INTERVIEWER FIND OUT & WRITE IN | 14 | |  |  | |  |
|  |  | | Prefer not to say | 97 | |  |  | |  |
|  |  | | Don’t know | 98 | |  |  | |  |

ASK ALL

**We have many experiences that may make us unsure about our future but we also have a sense of how we can make changes in our lives. I would like to ask you a few questions about how you see your life.**

| Q252. | | **Who do you think will contribute most to any future change in your life?**  CODE A MAXIMUM OF TWO RESPONSES | | | | |  | |
| --- | --- | --- | --- | --- | --- | --- | --- | --- |
|  |  | |  |  |  |  | |  |
|  |  | | Myself | 1 |  |  | |  |
|  |  | | My family | 2 |  |  | |  |
|  |  | | Our community | 3 |  |  | |  |
|  |  | | The local government | 4 |  |  | |  |
|  |  | | The state government | 5 |  |  | |  |
|  |  | | Other (SPECIFY) | 6 |  |  | |  |
|  |  | | None of these | 7 |  |  | |  |
|  |  | | Prefer not to say | 97 |  |  | |  |
|  |  | | Don’t know | 98 |  |  | |  |

| Q253. | | SHOWCARD 94 **How easy or difficult is it for people like you to change things in their local community if they want to?** SINGLE CODE ONLY | | | | |  | |
| --- | --- | --- | --- | --- | --- | --- | --- | --- |
|  |  | |  |  |  |  | |  |
|  |  | | Very easy | 1 |  |  | |  |
|  |  | | Fairly easy | 2 |  |  | |  |
|  |  | | Fairly difficult | 3 |  |  | |  |
|  |  | | Very difficult | 4 |  |  | |  |
|  |  | | Impossible | 5 |  |  | |  |
|  |  | | Don’t know | 98 |  |  | |  |

| Q254. | | SHOWCARD 95 **And to what extent, if at all do you feel you have control in making your own personal decisions that affect everyday activities?** SINGLE CODE ONLY | | | | |  | |
| --- | --- | --- | --- | --- | --- | --- | --- | --- |
|  |  | |  |  |  |  | |  |
|  |  | | A great deal of control | 1 |  |  | |  |
|  |  | | A fair amount of control | 2 |  |  | |  |
|  |  | | Not very much control | 3 |  |  | |  |
|  |  | | No control at all | 4 |  |  | |  |
|  |  | | Don’t know | 98 |  |  | |  |

Q255 AND Q256 TO BE ASKED IN SOUTH SUDAN ONLY.

| Protection of Civilian / Internally Displaced Person Camps |
| --- |

| Q255. | | **In the last three years, have you lived in a Protection of Civilian (PoC) or Internally Displaced Person (IDP) camp?**  SINGLE CODE ONLY | | | | |  | |
| --- | --- | --- | --- | --- | --- | --- | --- | --- |
|  |  | |  |  |  |  | |  |
|  |  | | Yes | 1 |  |  | |  |
|  |  | | No | 2 |  |  | |  |
|  |  | | Prefer not to say | 99 |  |  | |  |

ASK Q256 IF YES AT Q255. OTHERS GO TO Q257.

| Q256. | | **On how many, if any occasions have you lived in a Protection of Civilian or Internally Displaced Person camp for a period of more than one week in the last three years?**  SINGLE CODE ONLY | | | | |  | |
| --- | --- | --- | --- | --- | --- | --- | --- | --- |
|  |  | |  |  |  |  | |  |
|  |  | | CODE ‘1’ AND WRITE IN THE NUMBER OF OCCASIONS | 1  ☐☐☐ |  |  | |  |
|  |  | | Prefer not to say | 97 |  |  | |  |
|  |  | | Don’t know | 98 |  |  | |  |

ASK ALL

| And finally |
| --- |

| Q257. | | **What brings you happiness in life?**  WRITE IN | | | | |  | |
| --- | --- | --- | --- | --- | --- | --- | --- | --- |
|  |  | |  |  |  |  | |  |
|  |  | |  | |  |  | |  |
|  |  | |  |  |  |  | |  |

| Q258. | | | **What are your hopes for the future?**  WRITE IN | | | | |  | |
| --- | --- | --- | --- | --- | --- | --- | --- | --- | --- |
|  |  | | |  |  |  |  | |  |
|  |  | | |  | |  |  | |  |
|  |  | | |  |  |  |  | |  |
| Q259. | | **In your opinion, what can the community do to reduce violence some women and girls face?**  WRITE IN | | | | | |  | |
|  |  | | |  |  |  |  | |  |
|  |  | | |  | |  |  | |  |

| RECONTACT |
| --- |

**PART A**

INTERVIEWER TO READ OUT: Thank you for taking part in this survey.  We would like to ask you if you would be willing to take part in follow-up research on this subject.  If you agree, we would pass on your name, address, and telephone number if you have one to the International Centre for Research on Women - ICRW.   We will not pass on any of the answers you have given in this survey to anyone.

ICRW have been commissioned to undertake focus groups and one-to-one interviews with men and women in <<country>>.  If you are agreeable, ICRW may contact you by July 2016.   They will explain they have been given your contact details by us from this survey and they will ask you if you would be willing to take part in their research. As with this survey, ICRW will treat your details, and anything you have to say, in the strictest of confidence.

If you agree that we can pass on your contact details to ICRW, we will ask you to sign to confirm you are happy to do so.  If you cannot write, an ‘X’ will be sufficient to act as a signature.  Please note that your contact details will be held securely and will not be passed on to anyone else, other than ICRW.  You will only be contacted by ICRW for the purposes of research, and for no other reason.

Can I now check if you would agree to be re-contacted for follow-up research purposes:

**Yes – I agree to be re-contacted by ICRW for follow-up research.** CONTINUE TO PART B

**No – I do not agree to being re-contacted by ICRW for follow-up research.** THANK AND CLOSE

**PART B**

IF THE RESPONDENT AGREES READ OUT THE FOLLOWING:

Thank you for agreeing to allow ICRW to contact you for this follow-up research. By agreeing to this, you consent to your contact details being passed on to ICRW for the purposes of inviting you take part in this further research only.  You understand that ICRW may contact you by July 2016 to ask you if you would like to part in a focus group or one-to-one interview.  You are under no obligation to take part.

SIGNED*: _______________________________________________________

Contact telephone number: _________________

Address line 1: __________________________

Address line 2: __________________________

Address line 3  __________________________

Address line 4: __________________________

*INTERVIEWER NOTE: If the respondent cannot write, ask them to place an ‘X’ in the signature section.  This will act as agreement that the respondent understands they are being asked to participant in follow-up research.  Please reiterate that there is no obligation to take part in the follow-up research.

**THANK AND CLOSE**

ALL INTERVIEWERS: PLEASE COMPLETE THIS SECTION IMMEDIATELY AFTER YOU HAVE FINISHED THE INTERVIEW

INTERVIEWER CHECKS

INTERVIEWER: RECORD WHO WAS PRESENT WHILE THE PARTICIPANT WAS BEING INTERVIEWED, DO NOT ASK. MULTICODE OKAY

| QC1 | | DURING THE INTERVIEW, WAS THE RESPONDENT…? | | | | |  | |
| --- | --- | --- | --- | --- | --- | --- | --- | --- |
|  |  | |  |  |  |  | |  |
|  |  | | ALONE | 1 |  |  | |  |
|  |  | | CHILDREN WERE PRESENT ALL OF THE TIME | 2 |  |  | |  |
|  |  | | CHILDREN WERE PRESENT SOME OF THE TIME | 3 |  |  | |  |
|  |  | | ADULTS WERE PRESENT ALL OF THE TIME | 4 |  |  | |  |
|  |  | | ADULTS WERE PRESENT SOME OF THE TIME | 5 |  |  | |  |

| INTERVIEWER RECORD END TIME |  |  |  |  |  |  |  |  |
| --- | --- | --- | --- | --- | --- | --- | --- | --- |
|  | Hours | | | | Mins | | | |
